# Supplementary material for: Defining the activity of pro-reparative extracellular vesicles in wound healing based on miRNA payloads and cell type-specific lineage mapping
Source: Mol Ther. 2024 Feb 19;32(9):3059–79. doi: 10.1016/j.ymthe.2024.02.019 (PMC11403212; doi:10.1016/j.ymthe.2024.02.019)
Supplement: Document S1. Figures S1–S10 and Tables S1–S8 [file mmc1.pdf]

YMTHE, Volume *32*

Supplemental Information

Defining the activity of pro-reparative extracellular vesicles in wound healing based on miRNA payloads and cell type-specific lineage mapping

Dong Jun Park, Wooil Choi, Sakeef Sayeed, Robert A. Dorschner, Joseph Rainaldi, Kayla Ho, Jenny Kezios, John P. Nolan, Prashant Mali, Todd Costantini, and Brian P. Eliceiri

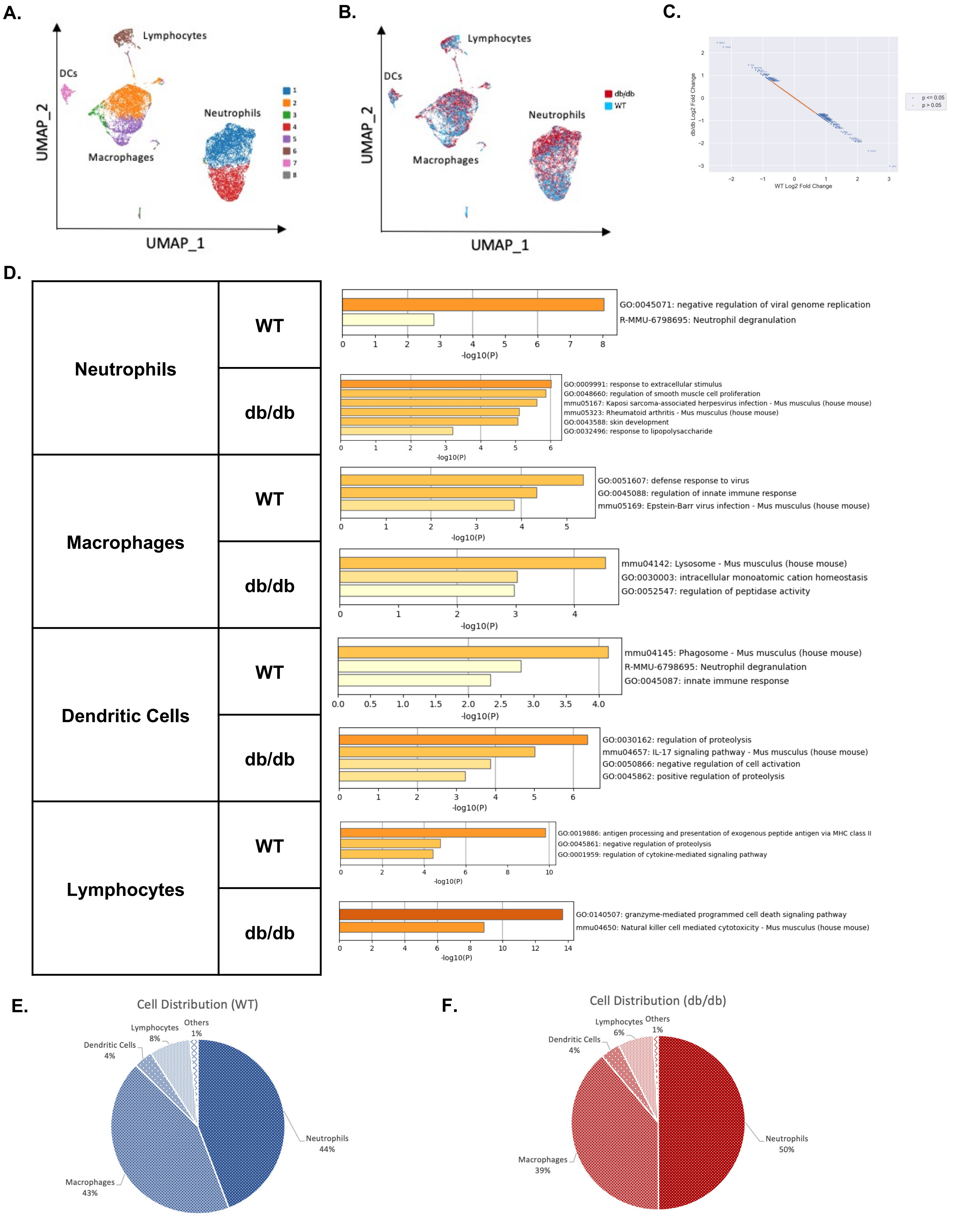

Figure S1. Supporting data for scRNAseq of cells isolated from WT vs db/db donor PVA sponges. (A) Distribution of cell types based on clustering of WT and db/db cells merged. (B) Comparison of cell type clusters based on WT (Blue) vs db/db (red). (C) Statistical analysis of changes in scRNAseq transcript levels in WT vs. db/db mice (n=3 pooled mice from each genotype). (D) GO term analysis comparing changes in gene expression focusing on neutrophils, macrophages, dendritic cells, and lymphocytes. (E and F) Distribution of cell types based on UMAP clustering of canonical genes for each cell type (See Supplementary Table 1 for gene list).

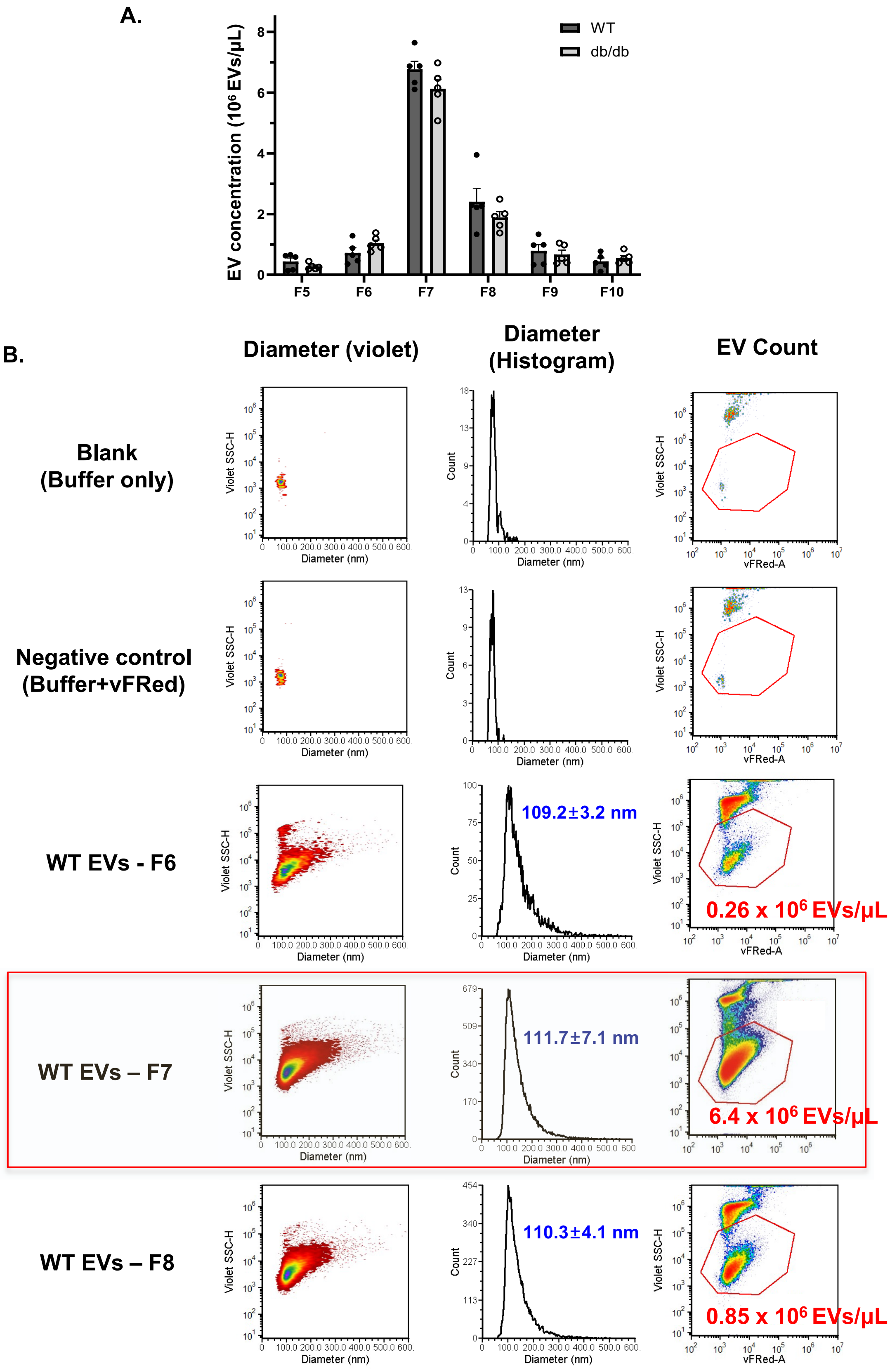

Figure S2. Controls for single vesicle flow cytometry (vFC) based on purification of samples by size exclusion chromatography (SEC). (A) Quantification of EV concentration in each fraction of the SEC from replicate WT vs db/db biological replicates (n=5 independent samples from each genotype) to demonstrate that similar concentrations of EVs recovered in each fraction. (B) Representative controls showing event distribution in a running buffer alone (Blank), Negative Control that has buffer with the fluorescent lipophilic membrane dye vFRed (Cellarcus, San Diego, CA) and no sample, and WT EVs which are samples from a SEC run where the three primary fractions that are recovered in F6-F8 are analyzed with the vFRed dye. In each case, the left column contains plots of size based on calibration with Nanorainbow beads (Cellarcus) vs. Violet SSC-H. The middle column shows diameter vs. count to obtain a size distribution and further supporting the ~110 nm population obtained from PVA sponges. The right column is a distribution of vFred^{+} events vs. Violet SSC-H with concentrated calculated based on events, rate, and volume.

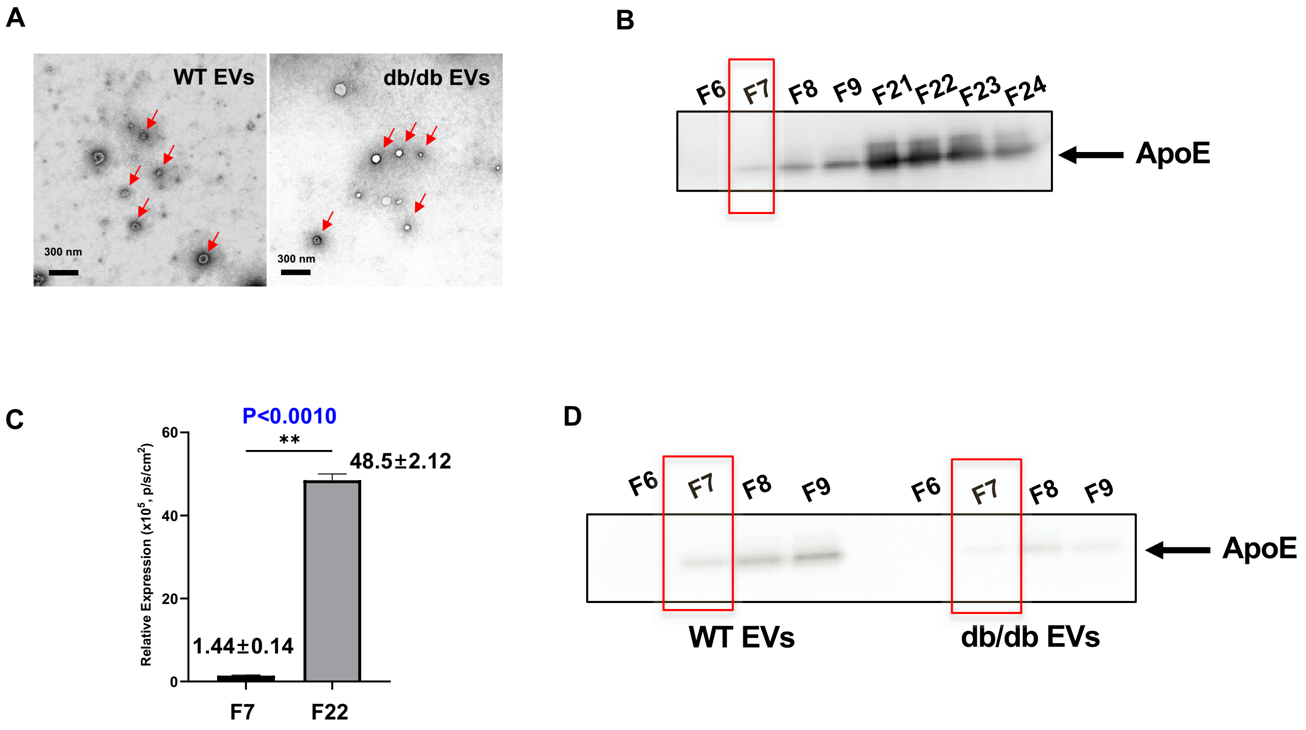

Figure S3. Characterization of EV fractions via electron microscopy (EM) and immunoblotting to monitor levels of ApoE lipoprotein in EVs. (A) Representative EM images of WT vs. db/db EVs. (B) Immunoblots to assess the extent to which size exclusion chromatography separates EVs from lipoproteins. Fractions F7 to F9, are the EV-enriched fractions while fractions and F21 to F23 are the ApoE-enriched fractions. Blots were normalized to have the same volume per fraction since the number of EVs was highest in F7 to F9 based on the data provided in Supplementary Figure 2. (C) Quantification of ApoE expression of a peak fraction from the EV-enriched range (F7) vs. the ApoE-enriched range (F22), showing there was 33.6 times lower ApoE in the EV fractions compared to F22 where ApoE containing lipoproteins would be expected to be detected in SEC. (D) For Panel D, the amount of protein added to each lane was normalized to have the same number of EVs for each fraction (i.e., WT vs db/db) based on the data provided in Supplementary Figure 2.

A.

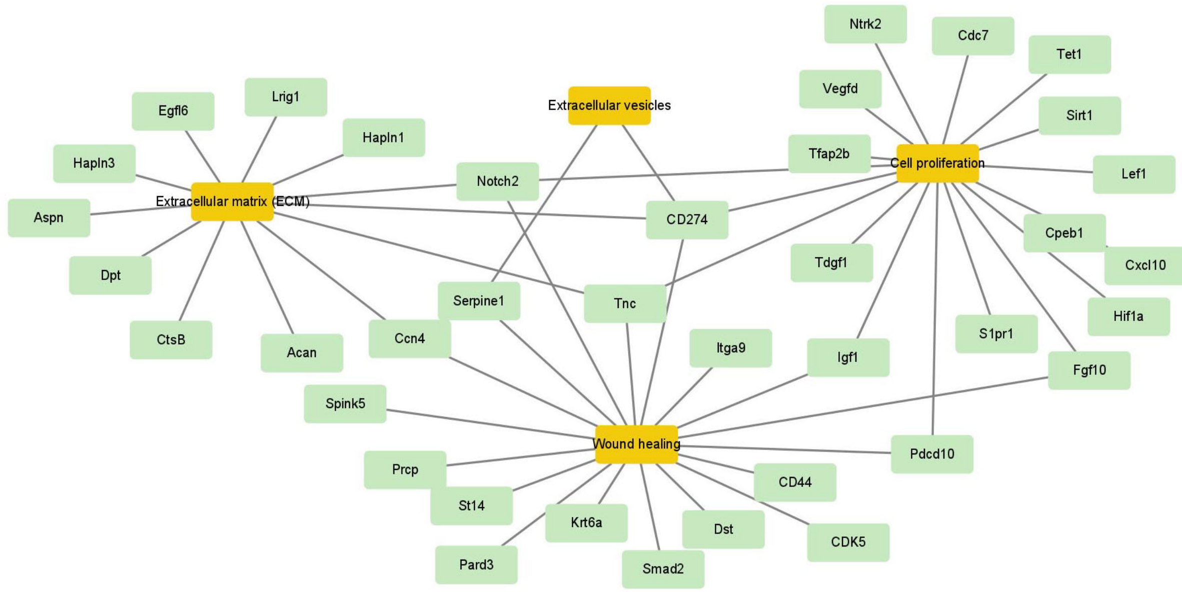

B.

GO ID	Transcript ID	Gene ID	Symbol	Gene_Annoation	GO Function	Target scan Score
GO:0016477	ENSMUST00000154617	ENSMUSG00000020516	Rps6kb1	ribosomal protein S6 kinase, polypeptide 1 [Source:MGI Symbol;Acc:MGI:1270849]	cell migration	99
GO:0010634	ENSMUST00000021530	ENSMUSG00000021109	Hif1a	hypoxia inducible factor 1, alpha subunit [Source:MGI Symbol;Acc:MGI:106918]	cell migration	98
GO:0030335	ENSMUST00000130310	ENSMUSG00000025586	Cpeb1	cytoplasmic polyadenylation element binding protein 1 [Source:MGI Symbol;Acc:MGI:108442]	cell migration	98
GO:0016477	ENSMUST00000030814	ENSMUSG00000028969	CDK5	cyclin-dependent kinase 5 [Source:MGI Symbol;Acc:MGI:101765]	cell migration	98
GO:0005518	ENSMUST00000021820	ENSMUSG00000021388	Aspn	asporin [Source:MGI Symbol;Acc:MGI:1913945]	Collagen binding	98
GO:0008284	ENSMUST00000146028	ENSMUSG00000020063	Sirt1	sirtuin 1 [Source:MGI Symbol;Acc:MGI:2135607]	Cell proliferation	97
GO:0044319	ENSMUST00000160272	ENSMUSG00000025812	Pard3	par-3 family cell polarity regulator [Source:MGI Symbol;Acc:MGI:2135608]	Wound healing	96
GO:0030335	ENSMUST00000029611	ENSMUSG00000027985	Lef1	lymphoid enhancer binding factor 1 [Source:MGI Symbol;Acc:MGI:96770]	cell migration	96
GO:0098633	ENSMUST00000173598	ENSMUSG00000063765	Chadl	chondroadherin-like [Source:MGI Symbol;Acc:MGI:3036284]	Collagen binding	95
GO:0016477	ENSMUST00000005218	ENSMUSG00000005087	CD44	CD44 antigen [Source:MGI Symbol;Acc:MGI:88338]	cell migration	94
GO:0031012	ENSMUST00000101126	ENSMUSG00000030029	Lrig1	leucine-rich repeats and immunoglobulin-like domains 1 [Source:MGI Symbol;Acc:MGI:107935]	Extracellular matrix (ECM)	93
GO:0030335	ENSMUST00000161137	ENSMUSG00000027835	Pdcd10	programmed cell death 10 [Source:MGI Symbol;Acc:MGI:1928396]	cell migration	92
GO:0008284	ENSMUST00000038816	ENSMUSG00000034855	Cxcl10	chemokine (C-X-C motif) ligand 10 [Source:MGI Symbol;Acc:MGI:1352450]	Cell proliferation	92
GO:0031012	ENSMUST00000005255	ENSMUSG00000005124	Ccn4	cellular communication network factor 4 [Source:MGI Symbol;Acc:MGI:1197008]	Extracellular matrix (ECM)	92
GO:0008284	ENSMUST00000079812	ENSMUSG00000027878	Notch2	notch 2 [Source:MGI Symbol;Acc:MGI:97364]	Cell proliferation	90
GO:0010634	ENSMUST00000022246	ENSMUSG00000021732	Fgf10	fibroblast growth factor 10 [Source:MGI Symbol;Acc:MGI:1099809]	cell migration	90
GO:0030335	ENSMUST00000038816	ENSMUSG00000034855	Cxcl10	chemokine (C-X-C motif) ligand 10 [Source:MGI Symbol;Acc:MGI:1352450]	cell migration	86
GO:0008284	ENSMUST00000129938	ENSMUSG00000029283	Cdc7	cell division cycle 7 (S. cerevisiae) [Source:MGI Symbol;Acc:MGI:1309511]	Cell proliferation	84
GO:0030335	ENSMUST00000205460	ENSMUSG00000030789	Itgax	integrin alpha X [Source:MGI Symbol;Acc:MGI:96609]	cell migration	83
GO:0070062	ENSMUST00000041388	ENSMUSG00000037411	Serpine1	serine (or cysteine) peptidase inhibitor, clade E, member 1 [Source:MGI Symbol;Acc:MGI:97608]	Extracellular vesicles	82
GO:0030335	ENSMUST00000197460	ENSMUSG00000032494	Tdgf1	teratocarcinoma-derived growth factor 1 [Source:MGI Symbol;Acc:MGI:98658]	cell migration	81
GO:0016477	ENSMUST00000146179	ENSMUSG00000020282	Rhbdf1	rhomboid 5 homolog 1 [Source:MGI Symbol;Acc:MGI:104328]	cell migration	78
GO:0008284	ENSMUST00000033751	ENSMUSG00000031380	Vegfd	vascular endothelial growth factor D [Source:MGI Symbol;Acc:MGI:108037]	Cell proliferation	76
GO:0008284	ENSMUST00000030056	ENSMUSG00000028364	Tnc	tenascin C [Source:MGI Symbol;Acc:MGI:101922]	Cell proliferation	76
GO:0030335	ENSMUST00000095360	ENSMUSG00000020053	Igf1	insulin-like growth factor 1 [Source:MGI Symbol;Acc:MGI:96432]	cell migration	73
GO:0008284	ENSMUST00000224259	ENSMUSG00000055254	Ntrk2	neurotrophic tyrosine kinase, receptor, type 2 [Source:MGI Symbol;Acc:MGI:97384]	Cell proliferation	71
GO:0005581	ENSMUST00000036737	ENSMUSG00000038591	Colec10	collectin sub-family member 10 [Source:MGI Symbol;Acc:MGI:3606482]	Collagen binding	71
GO:0008284	ENSMUST00000050826	ENSMUSG00000047146	Tet1	tet methylcytosine dioxygenase 1 [Source:MGI Symbol;Acc:MGI:1098693]	Cell proliferation	70
GO:0004867	ENSMUST00000069245	ENSMUSG00000055561	Spink5	serine peptidase inhibitor, Kazal type 5 [Source:MGI Symbol;Acc:MGI:1919682]	serine-type inhibitor activity	70
GO:0008284	ENSMUST00000027059	ENSMUSG00000025927	Tfap2b	transcription factor AP-2 beta [Source:MGI Symbol;Acc:MGI:104672]	Cell proliferation	69
GO:0042060	ENSMUST00000168423	ENSMUSG00000024563	Smad2	SMAD family member 2 [Source:MGI Symbol;Acc:MGI:108051]	Wound healing	69
GO:0030335	ENSMUST00000016640	ENSMUSG00000016496	CD274	CD274 antigen [Source:MGI Symbol;Acc:MGI:1926446]	cell migration	67
GO:0016477	ENSMUST00000055676	ENSMUSG00000045092	S1pr1	sphingosine-1-phosphate receptor 1 [Source:MGI Symbol;Acc:MGI:1096355]	cell migration	66
GO:0008236	ENSMUST00000076052	ENSMUSG00000061119	Prcp	prolylcarboxypeptidase (angiotensinase C) [Source:MGI Symbol;Acc:MGI:1919711]	serine-type peptidase	66
GO:0004252	ENSMUST00000234712	ENSMUSG00000033825	Tpsb2	tryptase beta 2 [Source:MGI Symbol;Acc:MGI:96942]	serine-type peptidase	63
GO:0016477	ENSMUST00000021028	ENSMUSG00000020689	Itgb3	integrin beta 3 [Source:MGI Symbol;Acc:MGI:96612]	cell migration	55
GO:0005581	ENSMUST00000170091	ENSMUSG00000025044	Msr1	macrophage scavenger receptor 1 [Source:MGI Symbol;Acc:MGI:98257]	Collagen binding	54
GO:0016477	ENSMUST00000123557	ENSMUSG00000031995	St14	suppression of tumorigenicity 14 (colon carcinoma) [Source:MGI Symbol;Acc:MGI:1338881]	cell migration	50
GO:0042060	ENSMUST00000183302	ENSMUSG00000026131	Dst	dystonin [Source:MGI Symbol;Acc:MGI:104627]	Wound healing	50

Figure S4. GO term analysis of candidate miR-425-5p targets.

(A) Interaction map highlighting potential relevance of miR-425-5p on cell proliferation, cell migration, and wound healing. (B) Table listing GO term-based target scan scores.

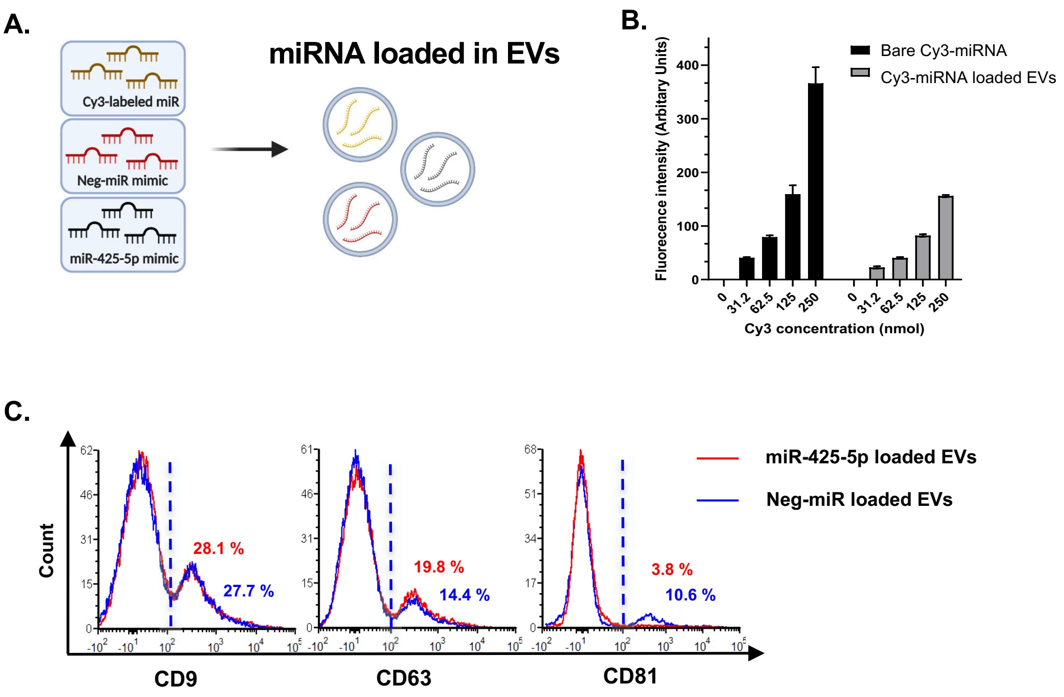

Figure S5. Optimization and encapsulation efficiency of miR-425-5p in EVs.

(A) Schematic of miRNA loading of EVs isolated from WT PVA sponge implants using Exofect per manufacturer's recommendation (Systems Biosciences, SBI). Neg-miR (cel-miR-67), miR-425-5p, and Cy3-labeled miRNA were compared, the Cy3-miRNA was provided as an internal control from SBI for titrating EV loading with respect to the amount of miRNA added. (B) Optimization of conditions for the encapsulation Cy-labeled miRNAs EVs using miRNAs in a range of 31-250 nmoles in a volume of 100 μL using a fluorescence plate reader. (C) Measurement of encapsulation efficiency based on Cy3-miRNA levels in EVs. (E) vFC using antibodies directed to the tetraspanins showing that the levels of CD9, CD63 and CD81 were unchanged between loading with miR-425-5p vs. Neg-miR loaded EVs before use for in vivo studies. Red line: miR-425-5p loaded EVs. Blue line: Neg-miR loaded EVs.

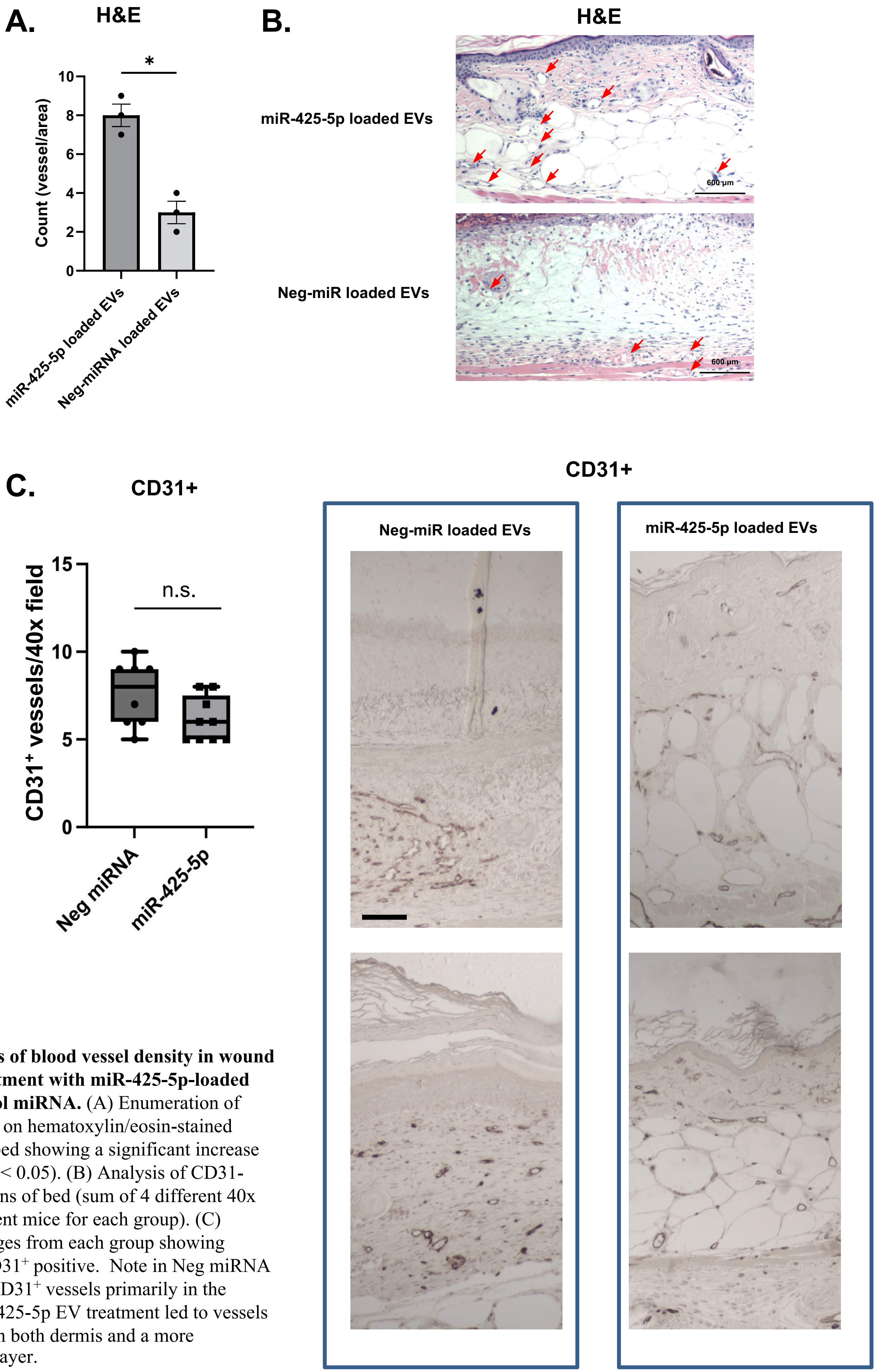

Figure S6. Analysis of blood vessel density in wound bed following treatment with miR-425-5p-loaded EVs vs. Neg control miRNA. (A) Enumeration of blood vessels based on hematoxylin/eosin-stained sections of wound bed showing a significant increase in vessel density ($P < 0.05$). (B) Analysis of CD31-stained tissue sections of bed (sum of 4 different 40x fields from 2 different mice for each group). (C) Representative images from each group showing similar levels of $CD31^{+}$ positive. Note in Neg miRNA EV treatment had $CD31^{+}$ vessels primarily in the dermis, while miR-425-5p EV treatment led to vessels widely distributed in both dermis and a more prominent adipose layer.

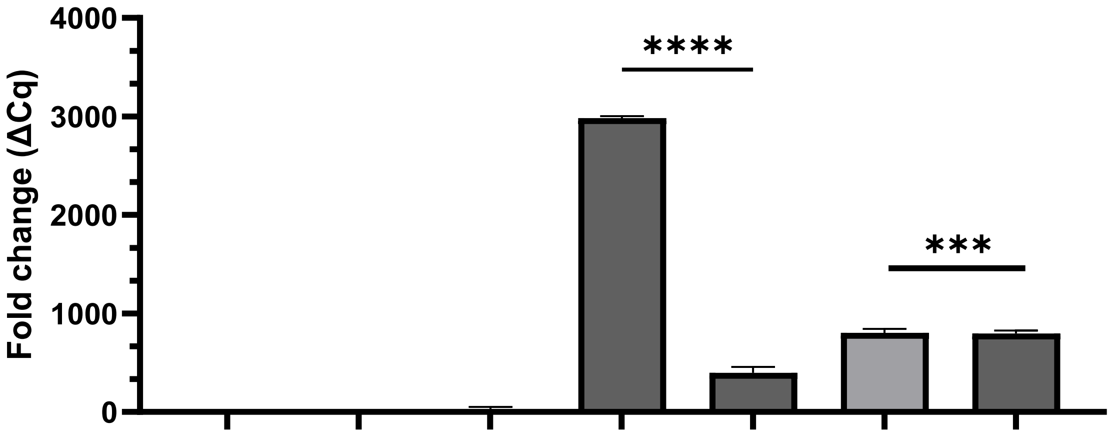

	1	2	3	4	5	6	7
EVs	-	+	+	-	-	+	+
miR-425-5p	-	-	-	+	+	+	+
Benzonase	+	-	+	-	+	-	+

Figure S7. Demonstration that EVs loaded with miR-425-5p were resistant to treatment with the nuclease benzonase. Incubation of nanoparticles such as EVs with benzonase can be used to determine whether nucleic acid payloads are on the surface or contained within a particle. Here we show that while treatment with bezonase degraded free miR-425-5p (Groups 4 and 5), treatment of EVs loaded with miR-425-5p did not show a significant reduction (Groups 6 and 7). We treated miR-loaded EVs, prepared using Exofect as described in the materials and methods (2.8 x 10^8 EVs), with benzonase nuclease (10 Unit/µL, 3 min). RNA was extracted with a mirVana™ miRNA Isolation Kit (Cat#AM1560, Thermo Fisher, Carlsbad, CA), and cDNA synthesized with the TaqMan™ Advanced miRNA cDNA Synthesis Kit (Cat# A28007, Thermo Fisher). qRT-PCR was performed on CFX96 (Bio-Rad) using the TaqMan™ Fast Advanced Master Mix for qPCR (Cat# 4444556, Thermo Fisher) and TaqMan™ Advanced miRNA Assay (mmu481161_mir) (Cat# A25576, Thermo Fisher). PCR reaction followed manufacturer's recommendations using the FAM fluorophore (Cycles: 95°C for 20 sec followed by 40 cycles of 95°C for 3 sec and 60°C 30 sec).

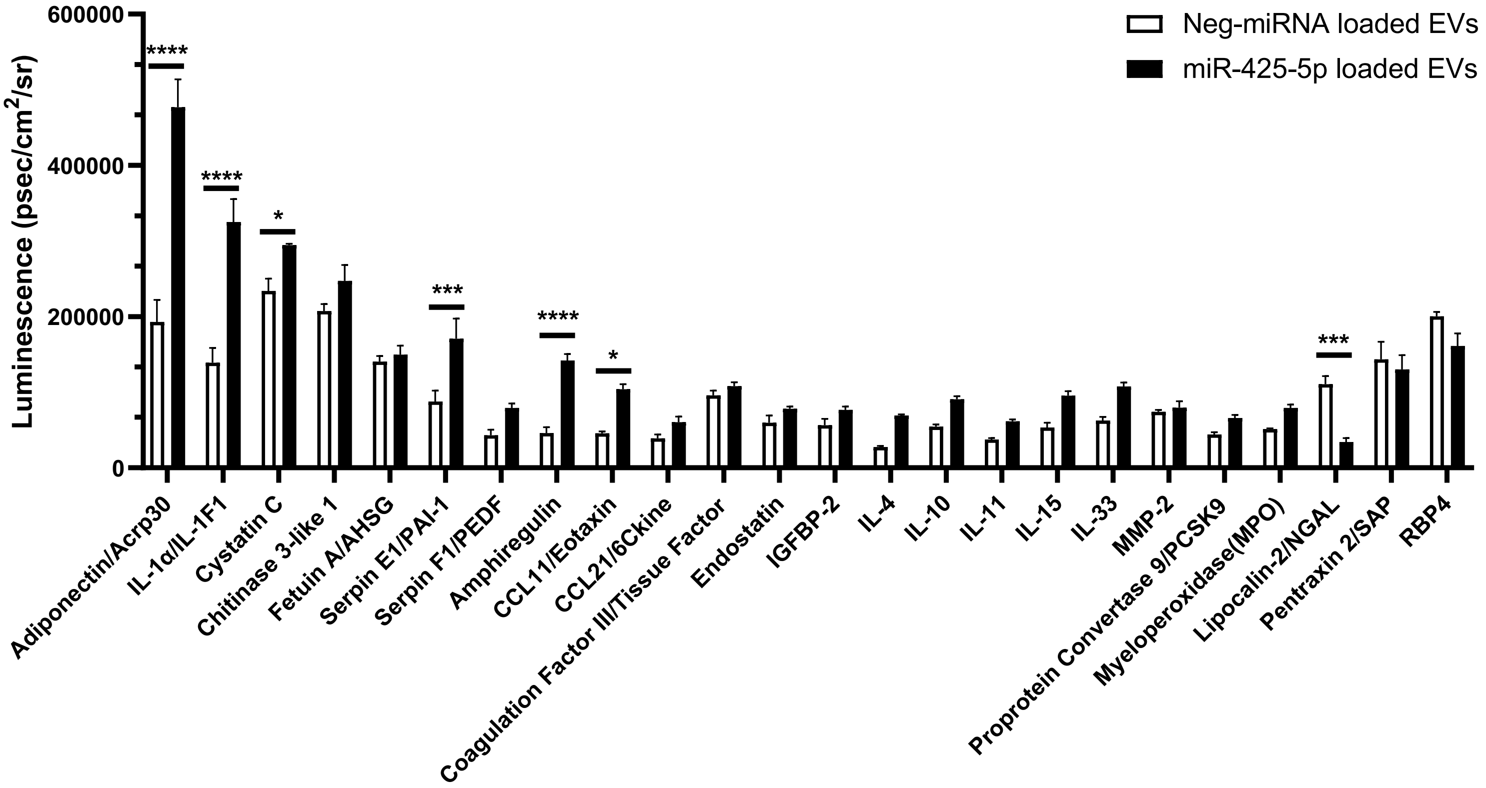

Figure S8. Bar graph of cytokine changes identified after treatment of miR-425-5p-loaded EVs vs. Neg-miRNA loaded EVs to the diabetic wounds. Adiponectin, IL-1α, Cystatin C, Serpin E1, Amphiregulin, and CCL11 were significantly increased when miR-425-5p-loaded EVs were treated to diabetic wounds. Conversely, Lipocalin-2 was significantly reduced when miR-425-5p-loaded EVs were treated to diabetic wounds. (p-value: ****<0.0001, ***<0.001, *<0.05 by Student t-test).

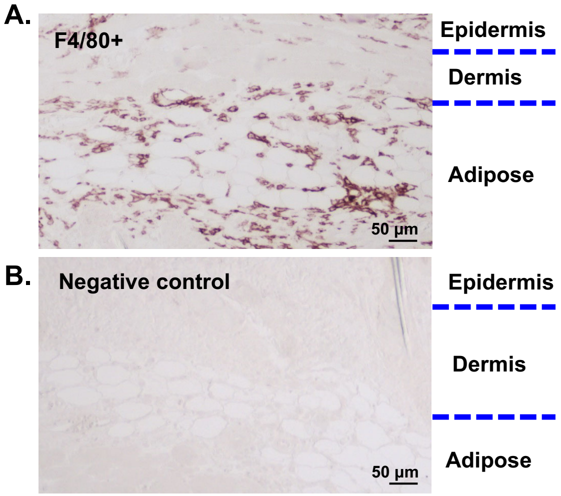

Figure S9. Expression of F4/80 positive macrophages generated in naïve adipose tissue.
(A) Localization of F4/80-positive cells in full thickness sections of mouse skin. (B) Negative control for the above using a secondary and detection reagent without the primary antibody.

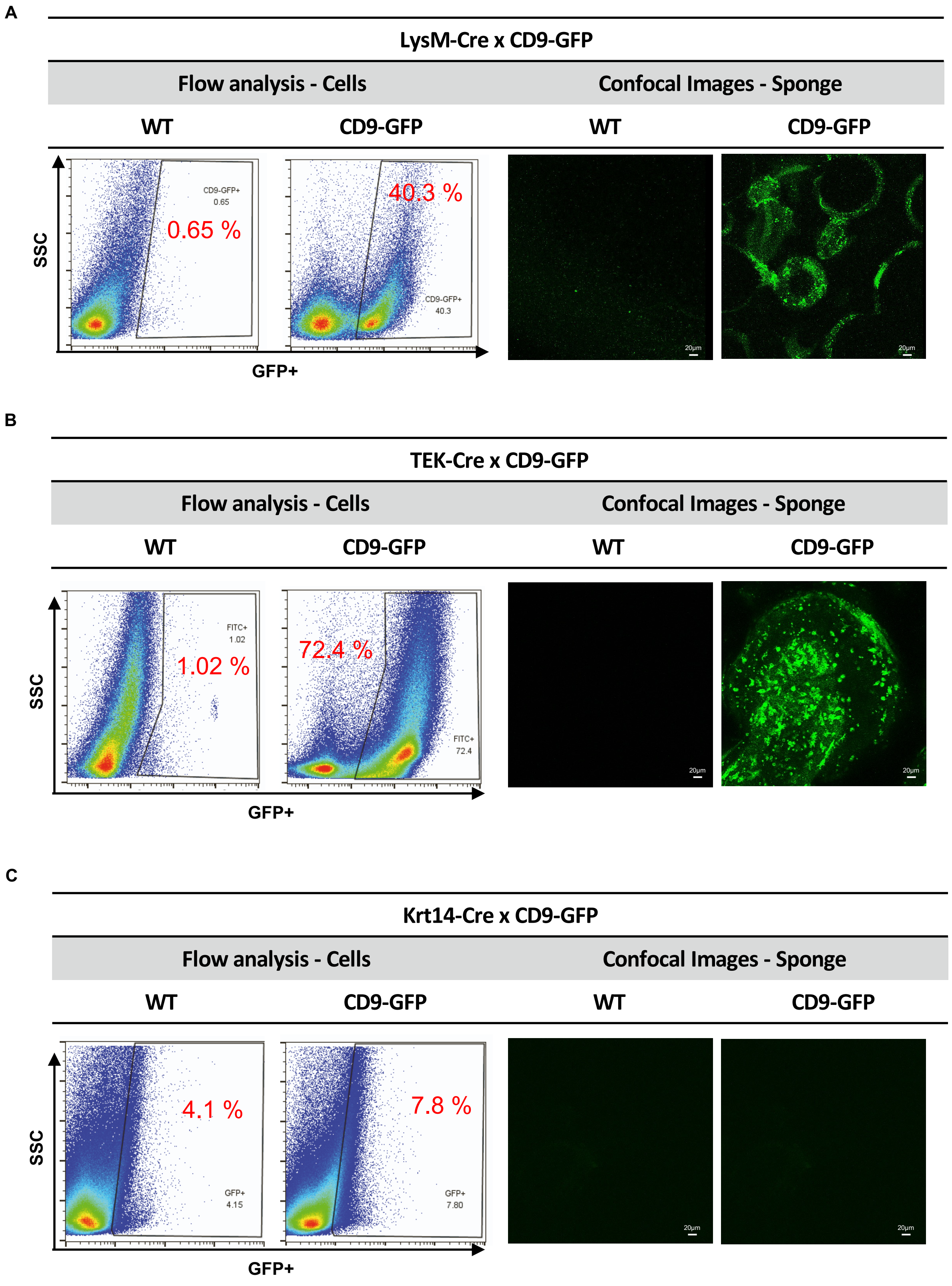

Figure S10. Analysis of GFP^{+} cells by flow cytometry and confocal microscopy to support EV analyses using cell-type specific promoter models. (A) Analysis of cells PVA sponge implants harvested from macrophage LysM-Cre x CD9-GFP vs. WT control mice. (B) Cells from endothelial TEK-Cre x CD9-GFP mice. (C) Cells from keratinocyte Krt14-Cre x CD9-GFP mice.

Table S1. The list of genes associated with each cell type identified by scRNAseq.

Cell Type	*Gene*	*References*
Macrophages	TREM2	Macrophage marker, inflammation and, atherosclerosis (PMID 35942822)
	IGF1	Adipocyte Macrophage crosstalk in obesity (PMID 28585206)
	H2-DMB1	Macrophages in a fatty liver disease (PMID 37474470)
	MS4A4C	Member of MS4A tetraspan family that modulates TREM2 (31413141)
Neutrophils	S100A8	S100A8/A9 in inflammation (PMID 29942307)
	S100A9	Oxidative stress in neutrophils in diabetes (PMID 34148367)
	CSF3	Neutrophil-mediated inflammation responses in infection (PMID 29883484)
Dendritic cells	CCR7	Regulation of DCs in migration and inflammation (PMID 35281921)
	ZBTB46	Transcriptional control of DCs (PMID 31759434)
Lymphocytes	CD3D	T lymphocytes regulating scarring in wound healing (PMID 31637099)
	CD4E	T cells in adverse cutaneous reactions (PMID 31395875)
	CD3G	T cell subsets in burn wounds (PMID 28575063)
	LTB	Leukotrienes in regulation of lymphoid cells (PMID 30477730)

Table S2. The list of up- and down- regulated miRNAs with target pathway and genes.

	miRNA	***Candidate Target pathway (miRPathDB v.2.0)***	***Gene Targets Observed in Proteome Profiler (Fig 6J) (TargetScanMouse V.7.1)***	***References***
Down-regulation	mmu-miR-425-5p	Dysregulated in Insulin/PI3K-AKT Signaling	SERPIN E1(ENSMUST00000041388.11)	Endothelial pro-survival, Angiogenesis, wound repair (PMID 35474734)<br>Adipose stem cell exosomes, regulate macrophage polarization, cardiac repair (PMID 35894060)
	mmu-miR-186-5p	Hippo signaling pathway	IL-15 (ENSMUST00000034148.7)	Wound healing, infection, immunoregulation (PMID 33017084)<br>Wound healing, macrophage migration, proliferation (PMID 37565786)
	mmu-miR-142a-5p	Focal adhesion	-	Promotes dendritic cell maturation (PMID 35668915)<br>Control profibrogenic macrophage (PMID 26436920)
	mmu-miR-361-3p	Proteoglycans in cancer	-	Wound healing, Migration, proliferation (PMID 34475974)<br>Wound healing, proliferation, inflammatory cell recruitment (PMID 30487794)
	mmu-miR-3068-3p	Metabolic pathways	-	Ischemic stroke, neuronal injury, excitotoxicity (PMID 31792968)
Up-regulation	mmu-let-7e-5p	Inflammatory response pathway	IL-10 (ENSMUST00000016673.6)	Inflammation, Oxidative stress, insulin signaling (PMID 33445738)<br>Inflammation, endotoxemia responses (PMID 33993048)
	mmu-miR-409-5p	Bacterial invasion of epithelial cells	-	Epithelial-mesenchymal transition, Tumorigenesis (PMID 25065597)<br>Regulates Wnt/beta catenin signaling (PMID 31165485)
	mmu-miR-181b-5p	MAPK signaling pathway	SERPIN E1(ENSMUST00000041388.11)	Wound healing, proliferation, inhibit apoptosis (PMID 30783419)<br>Diabetic wounds, senescence, angiogenesis, wound healing (PMID 35528840)
	mmu-miR-541-5p	ErbB signaling pathway	-	Proliferation, hyperglycemia-induced migration, angiogenesis dysfunction (PMID 36291965)<br>Cell differentiation, proliferation, innate immune response, anticoagulation mechanisms (PMID 37996554)

Table S3. The list of animals and cell line.

NAME	SOURCE	CATALOG #
C57B6/J	Jackson lab	JAX000664
$Lepr^{db/db}$	Jackson lab	JAX000697
B6.129P2-$Lyz2^{tm1(cre)Ifo}$/J	Jackson lab	JAX004781
B6.Cg-Tg(Tek-cre)12Flv/J	Jackson lab	JAX004128
B6N.Cg-Tg(KRT14-cre)1Amc/J	Jackson lab	JAX018964
B6;129S1-Gt(ROSA)$26Sort^{m1(CAG-CD9/GFP)Dmfel}$/J	Jackson lab	JAX033361
HEK293T	ATCC	CRL-1573
Mouse embryonic fibroblasts (MEFs)	ATCC	SCRC-1008

Table S4. The list of critical commercial assays kit.

NAME	SOURCE	CATALOG #
Chromium Next GEM Single Cell 3' Kit	10X Genomics	Cat# 1000269 (ver 3.1)
vFC analysis assay kit	Cellarcus Biosciences	Cat# CBS4HP-1PE
ExoQuick kit-TC	System Biosciences	Cat# EQULTRA-20A-1
MACSPlex Exosome Kit, mouse	Miltenyi Biotec	Cat# 130-122-211
BCA assay kit	Thermo Fisher	Cat# 23225
Exo-Fect™ Transfection kit	System Biosciences	Cat# EXFT20A-1
Mouse XL Cytokine Array kit	R & D Systems	Cat# ARY028

Table S5. The list of oligonucleotides for miRNA assay and Taqman qPCR.

NAME	SOURCE	CATALOG #
miR-425-5p mimic	Horizon	Cat# C-310988-01-0050
Negative-miR mimic (cel-miR-67; from C. elegans)	Horizon	Cat# CN-001000-01-50
Cy3-labeled miR (from SBI Kit)	System Biosciences	Cat# EXFT20A-1
mirVana™ miRNA Isolation Kit, with phenol	Thermo Fisher	Cat# AM1560
TaqMan™ Advanced miRNA cDNA Synthesis Kit	Thermo Fisher	Cat# A28007
TaqMan™ Fast Advanced Master Mix for qPCR	Thermo Fisher	Cat# 4444556
TaqMan™ Advanced miRNA Assay (mmu481161_mir)	Thermo Fisher	Cat# A25576

Table S6. Antibodies for immunoblotting and vFC.

Antibodies		
**NAME**	**SOURCE**	**CATALOG #**
CD9-PE (mouse)	Miltenyi Biotec	Cat# 130-123-052
CD81-PE (mouse)	Miltenyi Biotec	Cat# 130-102-632
CD63-PE (mouse)	Miltenyi Biotec	Cat# 130-123-289
MHC class I-PE (mouse)	Biolegend	Cat# 114607
CD29-PE (mouse)	Miltenyi Biotec	Cat# 130-119-165
CD274(PD-L1)-PE (mouse)	Biolegend	Cat# 124307
CD39-PE (mouse)	Miltenyi Biotec	Cat# 130-114-357
CD44-PE (mouse)	Miltenyi Biotec	Cat# 130-118-694
CD45-PE (mouse)	Miltenyi Biotec	Cat# 130-110-797
CD11b-PE (mouse)	Miltenyi Biotec	Cat# 130-113-806
CD54(ICAM-1)-PE (mouse)	Miltenyi Biotec	Cat# 130-104-214
CD49e-PE (mouse)	Miltenyi Biotec	Cat# 130-122-072
CD24-PE (mouse)	Miltenyi Biotec	Cat# 130-110-826
CD126(IL-6R)-PE (mouse)	Biolegend	Cat# 115806
CD66a-PE (mouse)	Miltenyi Biotec	Cat# 130-125-525
CD326(Ep-CAM)-PE (mouse)	Biolegend	Cat# 118205
Isotype-PE	Miltenyi Biotec	Cat# 130-113-450
Anti-Alix (E6P9B) mAb (mouse)	Cell signaling	Cat# 92880
Anti-CD9 (mouse)	Thermo Fisher	Cat# PA5-85955
Anti-CD81 (D5O2Q) (mouse)	Cell signaling	Cat# 10037
Anti-CD63 (mouse)	Thermo Fisher	Cat# PA5-92370
Anti-FLAG antibody	Sigma Aldrich	Cat# F-1804
Anti-mouse IgG, HRP-linked antibody	Cell signaling	Cat# 7076
Anti-rabbit IgG, HRP-linked antibody	Cell signaling	Cat# 7074
Anti-Rabbit HRP Polymer	Cell IDX	Cat# 2RH-100
Ki-67 Rabbit monoclonal Antibody (1:50)	GeneTex	Cat# 16667
Vimentin (D21H3) mAb	Cell signaling	Cat# 5741
Goat anti-Rabbit IgG-Alexa Fluor 546	Thermo Fisher	Cat# A11010
Goat anti-Mouse IgG-Alexa Fluor 546	Thermo Fisher	Cat# A11030
Anti-FLAG-vTag™ Antigody	Cellarcus Biosciences	Cat# CBS18-PE-100T
Goat anti-Rabbit IgG (H+L) Cross-Adsorbed Secondary Antibody, Alexa Fluor™ 488	Thermo Fisher	Cat# A-11008
ApoE	Cell signaling	Cat#49285

Table S7. The list of chemicals and sources.

Antibodies		
**NAME**	**SOURCE**	**CATALOG #**
DMEM-high glucose medium	Thermo Fisher	Cat# 12430054
Fetal Bovine Serum (FBS)	Sigma Aldrich	Cat# F0926
Antibiotic-Antimycotic (100X)	Thermo Fisher	Cat# 15240062
Lipofectamine 3000	Thermo Fisher	Cat# L3000150
RIPA Lysis and Extraction Buffer	Thermo Fisher	Cat# 89901
Tissue-Tek® O.C.T. Compound	Sakura	Cat# 4583
Trizol	Thermo Fisher	Cat# 15596018
RNA 6000 Nano	Agilent	Cat# 5067-1511
NEB Next® Magnesium RNA Fragmentation Module	NEB	Cat# e6150
Super Script™ II Reverse Transcriptase	Invitrogen	Cat# 1896649
DNA polymerase I (E.coli)	NEB	Cat# m0209
RNase H	NEB	Cat# m0297
dUTP Solution	Thermo Fisher	Cat# R0133
UDG enzyme	NEB	Cat# m0280
PVA sponge (7mm x 3mm Disk)	PVA Unlimited Inc.	Cat# SQ5000<br>PVA; PO#PR00440056
Oasis Nylon Suture	Thermo Fisher	Cat# MV-663-V-19mm
Betadine (10% povidone-iodine topical solution)	Thermo Fisher	Cat# NDC 67618-150-01
4 mm punch	Thermo Fisher	Cat# P450
Silicone ring (Thickness: 0.5 mm, outside diameter: 12 mm, inside diameter: 6 mm)	MCS	Cat# 33350174
Surgical glue	Thermo Fisher	Cat# 1469SB
Tegaderm	3M	Cat# 1622w
qEV1-column	IZON	Cat# ICI-70
MACSQuant Running Buffer	Miltenyi Biotec	Cat# 130-092-747
10% BSA stock solution	Miltenyi Biotec	Cat# 130-091-376
vCalTM nano rainbow beads	Cellarcus Biosciences	Cat# CBS6M
vFRed dye	Cellarcus Biosciences	Cat# CBS4A
vFC staining buffer	Cellarcus Biosciences	Cat# CBS0
Liposome control	Cellarcus Biosciences	Cat# CBS1
mouse platelet derived EVs	Cellarcus Biosciences	Cat# CBS2
96-well v bottom plate	Sarstedt	Cat# NC0068972
NuPAGE™ LDS Sample Buffer	Thermo Fisher	Cat# NP0008
1 mm pre-cast 12% Bis-Tris Mini Gel	Thermo Fisher	Cat# NP0342BOX
PVDF membrane	Thermo Fisher	Cat# LC2005
Memcode stain	Thermo Fisher	Cat# 24585
Pierce™ ECL Reagent	Thermo Fisher	Cat# 32209
1X Tris-buffered saline	CST	Cat# 9997
EM grids	Ted Pella, Inc.	Cat# 01754-F
Benzonase	Sigma Aldrich	Cat# E1014-5kU

Table S8. The list of analysis software and equipment.

NAME	SOURCE	CATALOG #
HiSeq sequencers	Illumina	https://www.illumina.com/systems/sequencing-platforms/hiseq-x.html
MACSQuant 10 (ver 2.13.2)	Miltenyi Biotec	https://www.miltenyibiotec.com/US-en/products/macsquant-analyzer-10.html
AXR confocal microscopy	Nikon	http://www.microscope.healthcare.nikon.com
Jeol 1400 plus TEM	Jeol USA	https://www.jeol.com/product/scientific/tem
Illumina Novaseq^{TM} 6000 platform	Illumina	https://www.illumina.com/systems/sequencing-platforms/
GraphPad Prism9 software	GraphPad Software	https://www.graphpad.com/
CytoFLEX S (Ver. 2.10)	Beckman Coulter	https://www.beckman.com/flow-cytometry/research-flow-cytometers/cytoflex
Xenogen IVIS-Lumina	Caliper life science Inc.	http://www.caliperls.com/assets/014/7156.pdf
Flowjo software (ver 10.7.1)	Flowjo, LLC	https://www.flowjo.com/
Cytoscape	NIGMS	https://cytoscape.org/
Image J	NIH	https://imagej.nih.gov/ij/download.html
OMERO	OME	https://www.openmicroscopy.org/omero/
FCS express	De Novo	https://denovosoftware.com/
R statistical language (ver. 3.3.3)	R foundation	https://www.r-project.org/
