# Supplementary material for: Defining the activity of pro-reparative extracellular vesicles in wound healing based on miRNA payloads and cell type-specific lineage mapping
Source: Mol Ther. 2024 Feb 19;32(9):3059–79. doi: 10.1016/j.ymthe.2024.02.019 (PMC11403212; doi:10.1016/j.ymthe.2024.02.019)
Supplement: Document S2. Article plus supplemental information [file mmc2.pdf]

Molecular Therapy
Original Article

Defining the activity of pro-reparative extracellular vesicles in wound healing based on miRNA payloads and cell type-specific lineage mapping

Dong Jun Park,[1] Wooil Choi,[1] Sakeef Sayeed,[1] Robert A. Dorschner,[2] Joseph Rainaldi,[3] Kayla Ho,[1] Jenny Kezios,[1] John P. Nolan,[4] Prashant Mali,[3] Todd Costantini,[1] and Brian P. Eliceiri[1,2]

[1]Department of Surgery, University of California San Diego, La Jolla, CA 92093, USA; [2]Department of Dermatology, University of California San Diego, La Jolla, CA 92093, USA; [3]Department of Bioengineering, University of California San Diego, La Jolla, CA 92093, USA; [4]Scintillon Institute, San Diego, CA 92121, USA

Small extracellular vesicles (EVs) are released by cells and deliver biologically active payloads to coordinate the response of multiple cell types in cutaneous wound healing. Here we used a cutaneous injury model as a donor of pro-reparative EVs to treat recipient diabetic obese mice, a model of impaired wound healing. We established a functional screen for micro-RNAs (miRNAs) that increased the pro-reparative activity of EVs and identified a down-regulation of miR-425-5p in EVs *in vivo* and *in vitro* associated with the regulation of adiponectin. We tested a cell type-specific reporter of a tetraspanin CD9 fusion with GFP to lineage map the release of EVs from macrophages in the wound bed, based on the expression of miR-425-5p in macrophage-derived EVs and the abundance of macrophages in EV donor sites. Analysis of different promoters demonstrated that EV release under the control of a macrophage-specific promoter was most abundant and that these EVs were internalized by dermal fibroblasts. These findings suggested that pro-reparative EVs deliver miRNAs, such as miR-425-5p, that stimulate the expression of adiponectin that has insulin-sensitizing properties. We propose that EVs promote intercellular signaling between cell layers in the skin to resolve inflammation, induce proliferation of basal keratinocytes, and accelerate wound closure.

INTRODUCTION

Healthy repair of cutaneous wounds is a coordinated response of hemostasis, immune cell recruitment, angiogenesis, and re-epithelialization[1,2]; however, dysregulation of these normal processes in diabetes, obesity, aging and infection presents a risk for chronic wounds.[3,4] Recent studies have identified extracellular vesicles (EVs), especially small EVs (50–120 nm), as the most abundant EV mediators of signaling crosstalk between mammalian cells.[5–7] In the context of wound healing, we have previously shown that small EVs comprise the vast majority of all EVs in the wound bed[8–10] and deliver biologically active nucleic acid and protein payloads, demonstrating their physiological relevance in intercellular signaling in skin injury.[8,11] Currently, many EV studies include the use of *in vitro*-cultured cells as EV donors that often test activity of human cell-derived EVs in mouse models.[12] The use of human-derived EVs in mouse models represents a significant limitation of the translational potential because of rejection considerations. An additional limitation is the overall lack of *in vivo* studies that address the heterogeneity of *in vivo* EV donors and the cell type of origin of EVs released into the wound microenvironment.[11,13,14]

Since the pro-reparative activity of EVs is generally associated with their ability to promote tissue repair by horizontal transfer of nucleic acids and proteins to recipient cells,[7,15] we developed an allograft model where EVs were harvested from subcutaneous implantations of sterile polyvinyl alcohol (PVA) sponges.[9,10] PVA sponge implants were originally developed as an animal model of foreign body response that we adapted for the efficient recovery of cells that release EVs relevant to the immune component of the injury response and for *in vivo* gene delivery to modify the activity of infiltrating cells.[16–18] The key advantages are that high concentrations of biologically active EVs are recovered using non-destructive approaches without the complications of blood products and culture media components used *in vitro*.[8] We previously used this model to identify the mobilization of human myeloid cells to PVA sponge implants in humanized mouse models, defined the activity of specific EV biogenesis genes that uncouple the production of pro-reparative EVs in wound healing, and used vesicle flow cytometry (vFC) to quantify EV heterogeneity *in vivo*.[9,10] Another key limitation of many wound healing studies of EVs as therapeutics is the lack of translationally relevant animal models of impaired tissue repair. We addressed this by focusing on testing of genetically defined mouse models of impaired wound healing, such as the leptin receptor knockout mouse, referred to as the db/db mouse, that has an onset of obesity and hyperglycemia at 12 weeks of age.[19–22] db/db mice are characterized by impaired wound healing kinetics and are an important genetic model for the study of injury-related complications in diabetes. Protease inhibitors were identified as down-regulated in EVs isolated

Received 12 September 2023; accepted 15 February 2024;
https://doi.org/10.1016/j.ymthe.2024.02.019.

Correspondence: Brian P. Eliceiri, Department of Surgery, University of California San Diego, La Jolla, CA 92093, USA.
E-mail: beliceiri@health.ucsd.edu

This is an open access article under the CC BY license (http://creativecommons.org/licenses/by/4.0/).

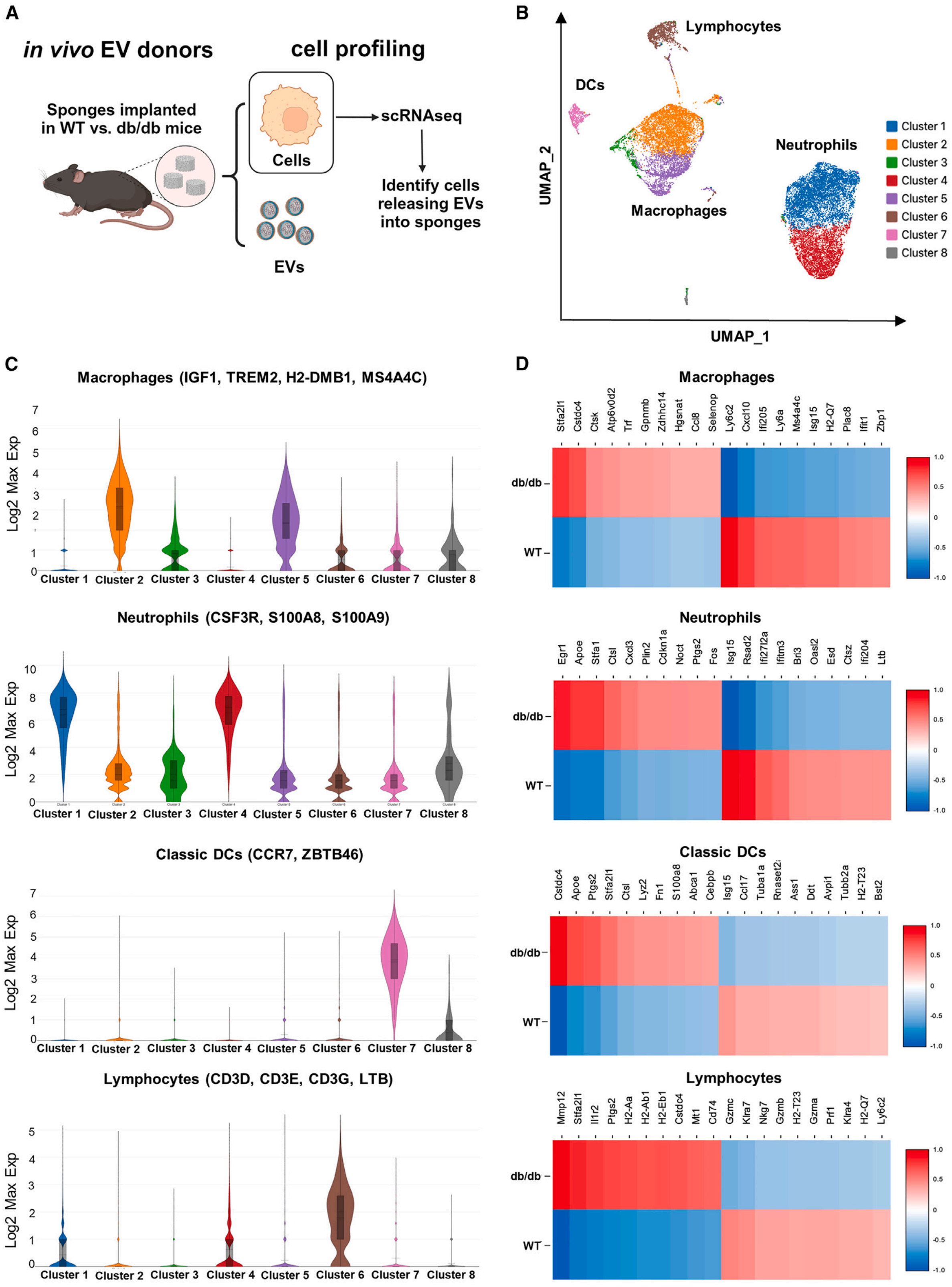

(legend on next page)

from db/db mice. In the context of functional testing of the biological activity of specific pro-reparative EV payloads, we have recently engineered the protein payload of the EVs. Specific protease inhibitors were over-expressed in engineered EVs and used to reverse the impaired wound healing phenotype.[8] Together, we identified the importance of using genetically defined donor mice for allograft studies of the biological activity of donor EVs to address a major challenge in the translational relevance of EVs as therapeutics and engineered EV payloads in cutaneous injury.

While the relative importance of EVs released from various cell types *in vivo* remains poorly understood, recent advances in the development of cell type-specific transgenic models have demonstrated the utility of tracking fluorescent EV reporters.[23–26] For example, expression of EV-associated proteins such as the tetraspanins CD9, CD63, and CD81 as fusion proteins with fluorescent reporter proteins can be used to identify EV distribution in the circulation and tumor microenvironment. Since these three tetraspanins are among the most highly enriched EV markers, they have been used to monitor EV trafficking *in vitro* in release and uptake studies.[25,26] In the context of cutaneous injury and our development of defined allograft models of EV release,[8–10] we used single cell RNA sequencing (scRNA-seq) to define the cellular landscape from where EVs are harvested in combination with transgenic mice expressing CD9 with a C-terminal GFP tag to determine the relative contributions of specific cell types in the donor site microenvironment.[24] These reporter approaches address key questions regarding the relative contributions of EVs released from different cell types, as well as being useful tools to assess the uptake of GFP^+ EV populations in recipient cells. In addition to GFP-based reporter models, we used single vFC to quantify the size, number, and expression of specific endogenous proteins presents on the surface of EVs, as well as tracking of epitope-tagged EV proteins.

In the field of cutaneous injury, intercellular communication can regulate differentiation and tissue injury responses between adipose, dermal, and epidermal cell layers. Directional movement of cells can also be controlled by persistent release of EVs that conditions the microenvironment, promotes adhesion, and regulates cell polarization.[25] Therefore, we focused on testing the pro-reparative activity of EVs isolated from wildtype (WT) vs. db/db animals implanted with PVA sponges that were used to collect donor site EVs. We identified EV-mediated differences in EVs from WT vs. db/db donors that regulated wound repair kinetics, changes in microRNA (miRNA) payloads and tested the activity of specific miRNAs by loading EVs and testing their capacity to restore tissue repair in the impaired wound healing model db/db recipient mice.[13]

Together, these studies take advantage of recent technological advances in vFC,[27] EV payload profiling by miRNA sequencing (miRNA-seq),[28] transgenic reporters to identify EV source,[29] and uptake in cell types relevant to wound repair.[6] Based on the distribution of macrophages interspersed in subcutaneous adipose tissue,[30] we propose that macrophage-derived EVs can be internalized by overlying fibroblasts, leading to the production of adipokines such as adiponectin that are pro-reparative and associated with increased cell proliferation of basal keratinocytes. Our data support a model in which EV-mediated acceleration of wound closure is regulated by specific miRNA payloads released by donor cells such as macrophages to affect the activity of overlying cell layers of the skin.

RESULTS

Diabetic obese mice drive a transcriptional reprogramming of immune cell subsets recruited into sites of cutaneous injury

To determine the source of EVs and identify biologically active payloads in impaired models of wound healing in the analysis of EVs from biological fluids, it was essential to have model systems with defined cell profiles. Therefore, we used scRNA-seq to identify the activation state of cell types infiltrating sterile subcutaneous PVA sponges implanted in the dorsum of WT mice (Figure 1A), a model that we and others have shown reflects the recruitment of macrophages and neutrophils observed in wound healing.[8–10,31] scRNA-seq was performed at 7 days after implantation, a time point that we previously showed was associated with peak of EV release,[8] to identify cells recruited to the PVA sponge. These cells were primarily macrophages and neutrophils, along with lower levels of dendritic cells (DCs) and lymphocytes, as seen on the UMAP projection (Figures 1B, S1A, and S1B). Expression of canonical genes associated with each cell type (Log2Max visualization of multiple genes based on Loupe Browser) (Figure 1C and Table S1) were used as the basis to identify cell type-specific changes in gene expression mediated by the loss of the leptin receptor in the db/db mouse model (Figure 1D). Cell type designations were based on canonical genes for each cell type (i.e., *Trem2* for macrophages, *S100a8* for neutrophils, *Ccr7* and *Zbtb46* for DCs, and *CD3* for lymphocytes).[32–42] Additional genes (i.e., *H2-dmb1* and *Ms4a4c*) were also identified as highly expressed in macrophages infiltrating PVA sponges (Table S1).[43,44] We identified changes in gene expression that were common among several cell types, such as increased levels of apolipoprotein E (*ApoE*), cathepsin L (*Ctsl*), prostaglandin synthase (*Ptgs2*), and cystatin domain proteins (*Cstdc4*). Cell type-specific changes in gene expression of the top 10 genes up-regulated vs. down-regulated genes of WT vs. db/db PVA sponges were observed in each major cell type. For example, we noted changes of specific genes in macrophages (*Stfa2l1* and *Trf*), neutrophils (*Egr*), DCs (*Lyz2* and *Ccl17*), and lymphocytes (*Mmp12* and *Il1r2*). scRNA-seq analysis identified additional changes in gene expression associated with the db/db mouse model that were primarily composed of metabolic factors associated with diabetic obesity (Figure S1D). Importantly, regardless of the genetic background of

Figure 1. Diabetic obese mice drive a transcriptional reprogramming of immune cell subsets recruited into sites of cutaneous injury

(A) Schematic of PVA implant model for the harvest of EVs from cutaneous site. (B) scRNA-seq of cells from PVA sponge implants in WT vs. db/db mice. (C) Expression of genesets mapping to macrophages, neutrophils, DCs, and lymphocytes based on supporting references in Table S1. (D) Analysis of changes in gene expression of top 10 up-regulated vs. down-regulated genes in cells from WT vs. db/db donors (GSE242496).

the donor mice, similar numbers of macrophages, neutrophils, DCs, and lymphocytes were recruited to the PVA sponge in db/db and WT mice (Figures S1E and S1F), and were consistent with analyses of cell types recruited to the PVA sponge based on antibody-dependent flow cytometry studies.[8] These findings identified macrophages and neutrophils as the predominant cell types in the PVA sponge model used as *in vivo* EV donors. In addition, these data showed that, although there were differences in the gene profile of cells harvested from WT vs. db/db mice, these changes were related to their physiology rather than affecting pathways directly related to EV release.

Characterization of EVs released into the biological fluid of a cutaneous injury site

Our previous studies established the efficacy of PVA sponge implants as an *in vivo* source of highly concentrated EVs relevant to wound healing.[8] We used these established standard parameters to analyze EVs purified from PVA implants in WT and db/db mice. EVs harvested from the PVA sponge implants in the wound fluid were subjected to serial centrifugation followed by size exclusion chromatography (SEC) (Figure 2A). We have previously shown that the most numerous EVs in subcutaneous implants of PVA sponges were 100–120 nm in diameter and comprised the vast majority of all EVs observed in this biological fluid, with relatively few larger EVs being observed.[8] Each fraction of the SEC was analyzed for EV concentration using vFC as assessed by staining with the fluorescent lipophilic membrane dye, vFRed (Figure 2B, column), and compared in parallel with the protein concentration in each fraction (Figure 2B, line) as detailed in the materials and methods. Each fraction of the SEC was further validated for EV content by immunoblotting for a canonical EV marker like the tetraspanin CD9. We identified high levels of CD9 protein in the EV containing fraction 7 by immunoblot (Figure 2C) and by vFC with a fluorescently labeled anti-CD9 antibody (Figure S2). Low levels of CD9 protein were detected in later fractions that lacked significant numbers of small EVs (i.e., fractions 15–20) (Figure 2C). Immunoblotting of whole cell lysates (WCLs) compared with purified EVs from the same WT PVA sponges demonstrated that CD9, CD63, CD81, and Alix were all expressed in mouse PVA sponge EVs (Figure 2D). Enrichment of CD9 and Alix in EVs vs. WCLs was noted in the analyses of mouse PVA sponge EVs, suggesting that these proteins may be more EV specific. For the characterization of EVs isolated from db/db mice, we established cohorts of 12- to 16-week-old WT and db/db mice, where db/db mice used for the collection of EVs were significantly more hyperglycemic (Figure 2E) and obese (Figure 2F) compared with WT mice. These two parameters were the hallmarks of the diabetic obese phenotype that are characteristic of the db/db mouse model. The concentration of EVs isolated from PVA sponges was in the range of $5–7 \times 10^6$ PVA EVs/μL (Figure 2G). EVs isolated from WT and db/db mice had similar size distributions, with the mean diameter of EVs detected being 116.7 ± 8.79 nm (n = 6) from db/db donors and 119.8 ± 6.8 nm (n = 6) from WT donors (Figure 2H). Similar sizing analysis of each of the other SEC fractions did not reveal any substantial numbers of larger EVs in later fractions (Figure 2B and data not shown). Transmission electron microscopy established that the EVs purified from WT and db/db donors had a similar size and shape (Figure S3A). To monitor for the potential of lipoprotein contamination of EV fractions collected by SEC, we performed immunoblotting of SEC fractions with an antibody to detect lipoproteins such ApoE that could be present in EV fractions. We confirmed that the EVs collected in early fractions of the SEC (i.e., fractions 6–9) (see Figure 2B) were well separated from lipoproteins observed collected in late fractions (i.e., fractions 21–23) (Figures S3B and S3C). We also observed that the levels of ApoE expression were unchanged between WT and db/db EVs (Figure S3D).[45,46] Together, these analyses established the purification, expression of canonical protein markers, size, and concentration from an *in vivo* EV donor model that is known to exhibit a well defined phenotype of impaired wound healing.[8]

Identification of changes in EV proteins isolated from diabetic obese donor model

To determine the profile of proteins expressed on the EV surface we used a combination of batch and single EV analysis (i.e., vFC) (Figure 3A). WT EVs were purified from cutaneous implants and subjected to a multiplex analysis (Figure 3B) that identified proteins associated with leukocytes (i.e., CD45, major histocompatibility complex MHC II, and CD20), leukocyte activation (CD44 and CD66a), and cell adhesion (CD49e, CD11b, and CD61). To address the heterogeneity of EVs in this biological fluid, we performed vFC to determine the expression of individual tetraspanins that are generally used as EV markers. We observed high levels of CD9 and CD63 expression on the surface of WT EVs (Figure 3C). Further, vFC analysis identified several immune cell-relevant proteins expressed on the surface of WT EVs such as MHC I, CD29 (ITGB1), CD274 (PD-L1), and CD39 (ENTPD1) (Figure 3D). These assays on WT EVs formed the basis for the vFC analysis of proteins expressed on biological replicates of WT vs. db/db EVs (n = 5 for each genotype). For example, we observed that expression levels of tetraspanins CD9 and CD63 measured by vFC were unchanged between WT and db/db EVs, thus providing a control for the levels of EVs collected from each genotype using canonical tetraspanin markers (Figure 3E). Based on the importance of integrins in mediating binding to the extracellular matrix, we next measured proteins levels of integrins by vFC. We noted reductions of in the number of EVs expressing detectable CD11b (ITGAM) (0.6-fold decrease; $p < 0.0026$) and CD49e (ITGA5) (0.69-fold decrease; $p < 0.0074$) in db/db vs. WT EVs, but no significant change for CD29 (ITGB1) (Figure 3F). We observed significant decreases in the number of EVs expressing detectable immune-related proteins CD45 (0.77-fold decrease; $p < 0.0285$), CD44 (hyaluronic acid receptor) (0.83-fold decrease; $p < 0.0246$), and CD54 (ICAM1) (0.75-fold decrease; $p < 0.0221$). In contrast, levels of CD274 (programed cell death ligand 1) were increased in db/db vs. WT EVs (1.47-fold increase; $p < 0.0345$). Levels of MHC I-positive EVs were unchanged (Figure 3G). We observed no significant changes in the number of EVs positive for other EV markers implicated in injury models, including CD326 (EPCAM), CD39 (ENTPD1), CD66a (CEACAM1), CD24 (HAS), and CD126 (IL6R) (Figure 3H).[47–51] Taken together, these findings suggested that the quantitative differences in the expression of select integrins and other

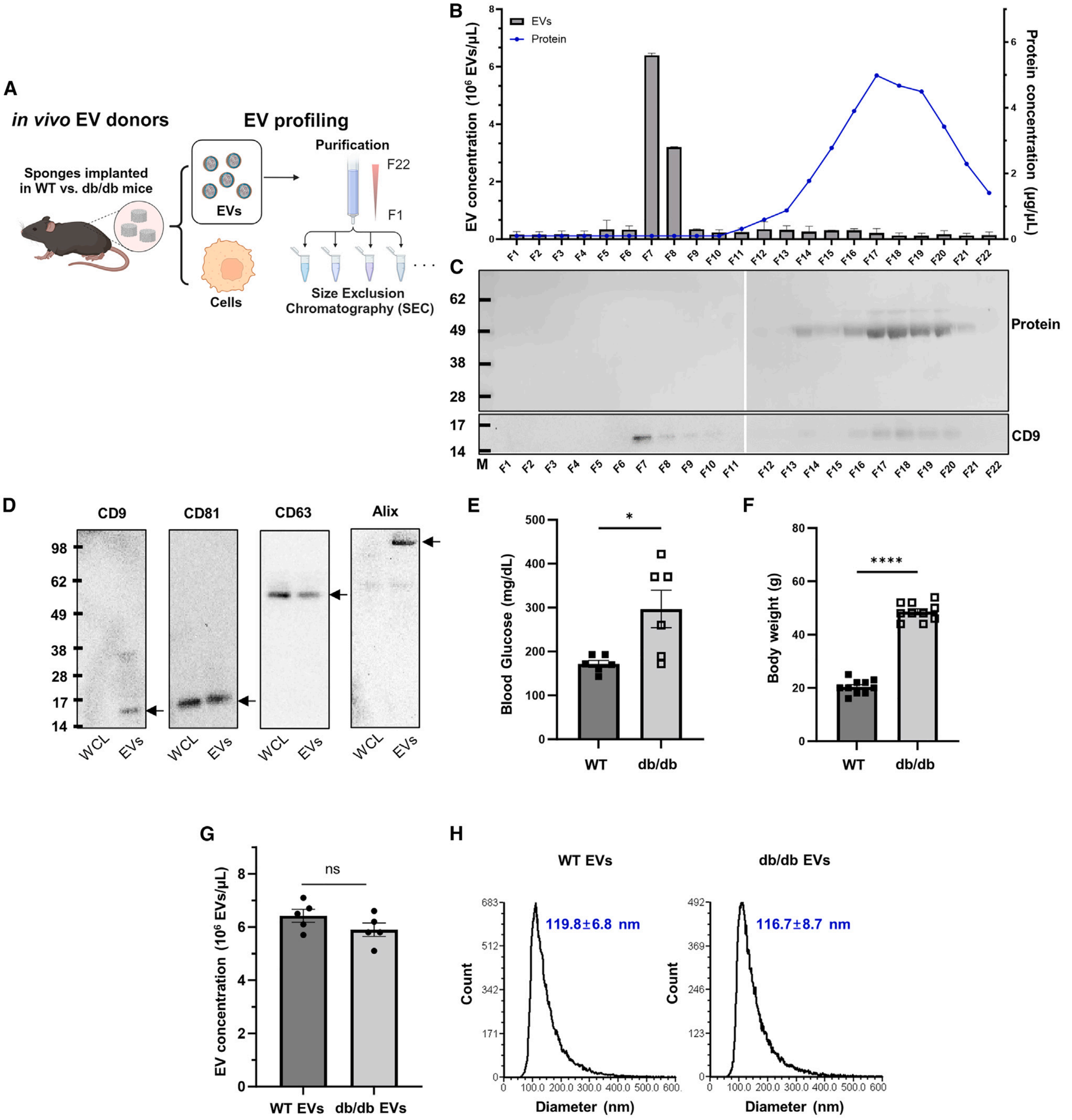

Figure 2. Identification and characterization of EVs derived from the PVA sponges

(A) Schematic of EV purification and profiling using SEC. (B) Quantification of SEC fractions by determining EV concentration using vFRed staining as detailed in the materials and methods (bars) and soluble protein using a BCA assay (line) from a representative WT EV sample. EV concentration of WT vs. db/db EVs from each SEC fraction is quantified in replicates in Figure S2A. (C) Immunoblotting of SEC fractions to detect CD9 (bottom) and a Memcode protein stain (top) for total protein from each SEC fraction. (D) Immunoblotting of EV markers of WCLs and EVs from a representative WT PVA donor. (E and F) (E) Blood glucose and (F) body weight measurements that define the pathophysiology of the db/db mouse model. (G and H) (G) EV concentration and (H) size distributions of WT vs. db/db EVs in fraction 7 (n = 10). *$p < 0.05$, ****$p < 0.0001$.

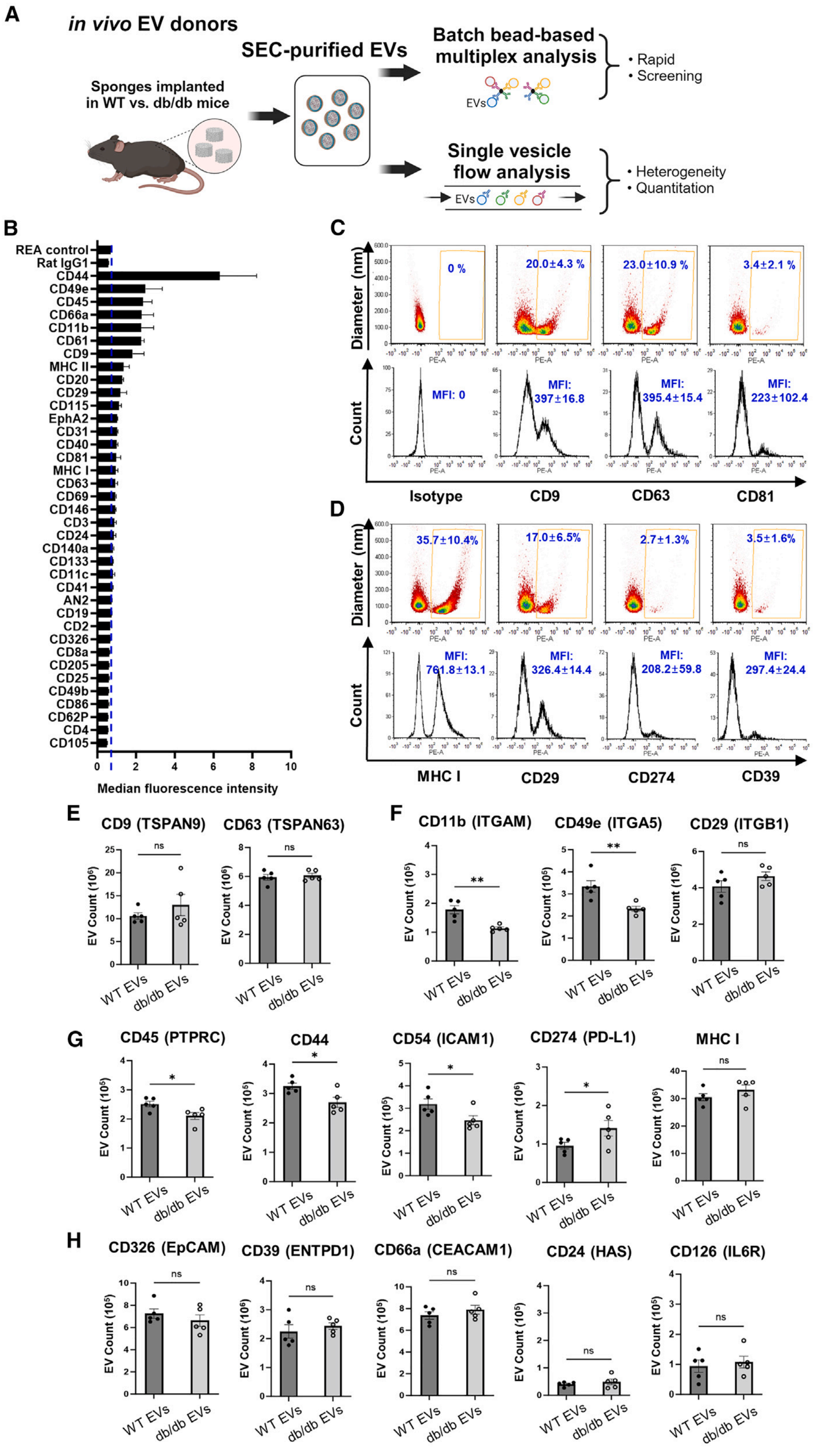

(legend on next page)

immune-related factors between WT and db/db EV donors may affect EV binding and activity in the wound bed.

EVs from diabetic obese donors have impaired wound healing activity

We previously reported that the pro-reparative phenotype of EVs in wound healing can be uncoupled by interference with specific EV biogenesis pathways,[9] and that the EV profile (i.e., expression of proteins on the surface of EVs) is regulated by the genetic background of immune-deficient and db/db donor models.[10] To define the activity of EVs from db/db vs. WT donors, we purified EVs from PVA sponge implants from WT and db/db mice as described above and applied these EVs to naive wounds (Figure 4A). Specifically, EVs were applied topically in a single dose (5–10 $\times$ 10^6 EV/50 μL/wound) to freshly prepared splinted full-thickness wounds into naive recipient db/db mice, the standard mouse model for impaired wound healing (Figure 4B). We observed that db/db donor EVs had a significant decrease in pro-reparative activity in wound healing compared with the treatment with WT control EVs at days 5, 7, 10, and 13 (n = 10) (Figure 4C) (**$p < 0.005$, *$p < 0.05$). Analysis of the kinetics of db/db EV-mediated wound closure was comparable to saline-treated controls (Figure 4C). Histological analysis revealed decreases in wound closure (Figure 4D) and statistically significant reductions in epidermal thickness (Figure 4E) ($p < 0.0001$), and dermal cellularity in the margins (Figure 4F; refer to brackets in Figure 4D for regions of analysis) ($p < 0.0001$) of wounds treated with db/db vs. WT EVs. To determine whether there were differences in epithelial cell proliferation in EV-treated wounds, a hallmark of the wound repair process,[52,53] we performed immunostaining with an anti-Ki67 antibody to localize the effects of EVs on proliferation. We observed that treatment with WT EVs promoted the proliferation of basal keratinocytes as detected by the increase in Ki-67$^+$ cells, a molecular endpoint that is physiologically relevant for wound closure, whereas there was an absence of Ki67$^+$ cells in basal keratinocytes of db/db EV-treated wounds was observed (Figure 4G). Given this effect of WT EV treatment stimulating the proliferation of basal keratinocytes, we focused on an EV-tagging strategy to assess the distribution of EVs in the wound bed to better understand what cells may uptake EVs based on localization of the EV tag. We designed an FLAG-tagged tetraspanin CD63 that would express the FLAG tag on the outside of the EV that took advantage of the transient transfection properties of a cell line like HEK293 to rapidly prepare high-purity EVs for biological testing. Since HEK293 cells are also widely used in the EV field for engineering and production,[54–56] we could purify EVs from the conditioned media of cultured HEK293 cells that either over-expressed human CD63 or CD63-FLAG (Figure 4H, top). We determined that the FLAG epitope was displayed on 29% of all EVs collected from the conditioned media (Figure 4H, bottom). FLAG expression was confirmed by immunoblotting of EVs from CD63-FLAG transfected cells vs. control CD63-transfected cells (Figure 4I). Next, CD63-FLAG- or CD63-expressing EVs were added to full-thickness wounds and incubated for 24 h. Upon harvest and immunohistochemical staining to detect the FLAG tag, we observed uptake of FLAG-tagged EVs in cells of the dermis, especially in the higher magnifications. FLAG-positive cells were observed primarily in the dermis, a cell layer characterized by an abundance of fibroblasts (Figure 4J). Wounds treated with untagged CD63 or saline-treated wounds were used as negative immunohistochemical controls (Figure 4J). Based on the substantial localization of FLAG-tagged EVs in the dermis, we further assessed the uptake of CD63-FLAG-expressing EVs into cultured primary fibroblasts using mouse embryonic fibroblasts (MEFs). MEFs were treated with CD63-FLAG-tagged EVs or control CD63 EVs and immune-stained with an anti-FLAG antibody and imaged by immunofluorescence (IF) (Figure 4K). We observed dose-dependent EV uptake into MEFs (Figure 4L). These findings showed that the activity of EVs administered to full-thickness wounds can be monitored by assessing cell proliferation as a molecular endpoint for the activity of pro-reparative EVs and that the uptake of EVs can be localized using molecular tags such as the FLAG tag.

Regulation of miRNA EV payloads isolated from diabetic obese donor model

Studies of miRNAs in EVs in diabetic wounds have established their translational relevance in wound healing.[13] Therefore, we analyzed changes in miRNA payloads from WT and db/db mice in our model. We performed miRNA-seq on EVs purified from WT and db/db donors harvested from the PVA sponge model and identified statistically significant changes in miRNAs from three biological replicates from WT and db/db EVs (data have been archived at NCBI #GSE242496) (Figure 5A). Many EV miRNAs were similar between WT and db/db EVs (Figure 5B), consistent with the concept many miRNAs could be considered housekeeping miRNAs.[57,58] However, of the miRNAs that were down-regulated more than 2-fold in db/db vs. WT EVs, changes in the following miRNAs were statistically significant: miR-425-5p (2.68-fold decrease), 361-3p (3.15-fold decrease), 3068-3p (3.15-fold decrease), and 186-5p (2.02-fold decrease). The only miRNA that was significantly up-regulated in EVs from db/db vs. WT donors more than 2-fold was miR-409-5p (2.38-fold increase) (Figure 5C). Kyoto Encyclopedia of Genes and Genomes (KEGG) pathway analysis (Figure 5D) of the overall changes of these db/db-regulated miRNAs were associated with signaling pathways relevant in diabetic wound healing and complications such as stem cell regulation and advanced glycation endproduct/receptor for advanced glycation endproduct signaling in diabetic complications that are relevant in impaired wound healing in a diabetic obese model.[59] To better understand the potential targets of individual miRNAs identified, we analyzed potential targets using miRPathDB v2.0 and performed a literature search as summarized

Figure 3. vFC of EVs isolated from PVA sponge implants

(A) Schematic of EV analyses by batch vs. vFC. (B) Quantification of a bead-based EV protein screen of inflammation-related proteins of WT EVs. (C–H) (C) Representative vFC analysis of isotype and tetraspanin levels on WT PVA EVs, and (D) immune-related proteins. vFC analysis of WT vs. db/db EVs for (E) tetraspanins, (F) integrins, (G) immune-related proteins, and (H) other EV-related inflammation proteins. (n = 5 for each group; **$p < 0.005$, *$p < 0.05$). MFI, mean fluorescent intensity.

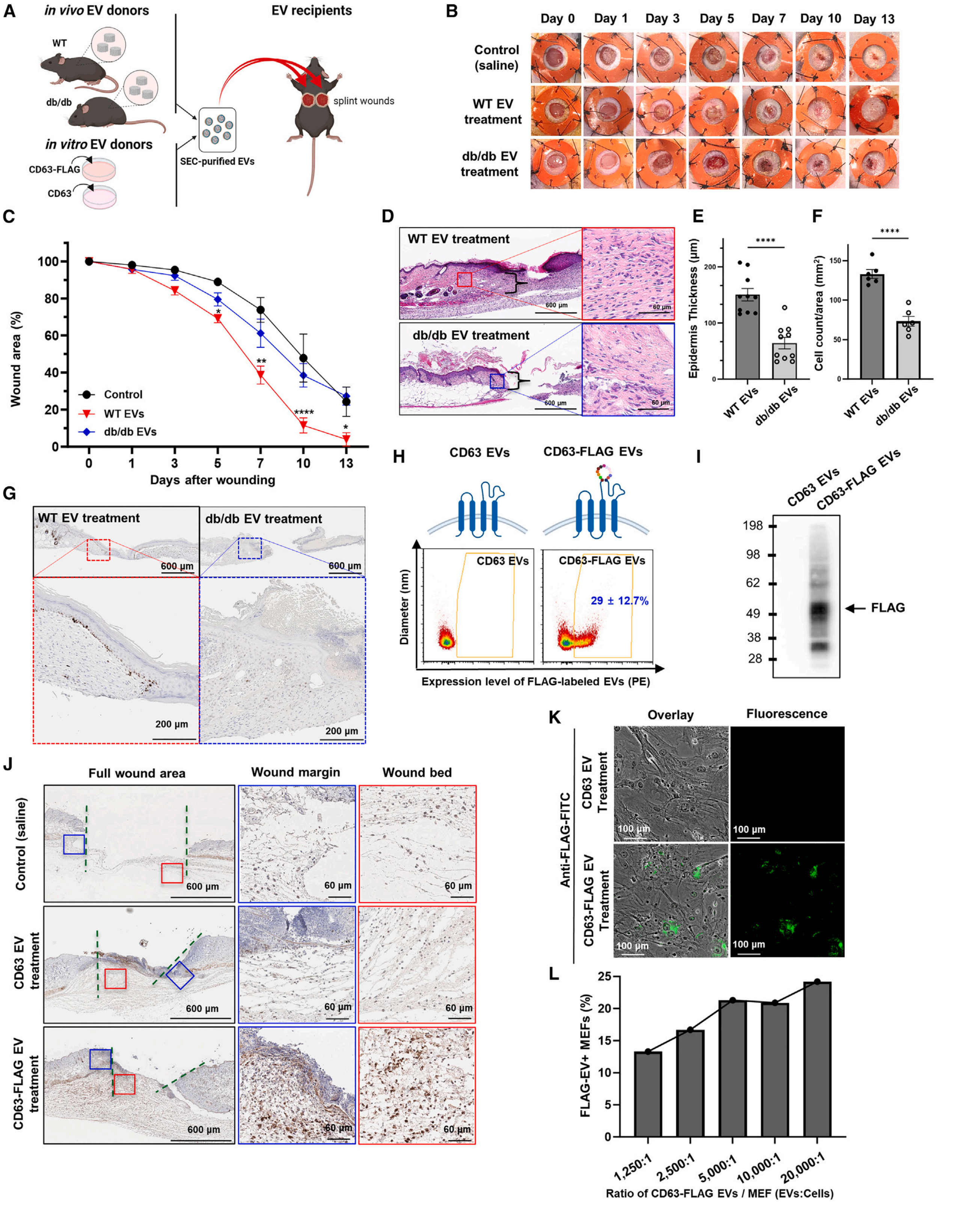

(legend on next page)

in Table S2. With this approach, we identified miR-425-5p as the lead candidate, as it had also been recently reported to mediate endothelial survival relevant to EV action in a streptozocin-induced diabetic mouse model,[60] stimulate cell proliferation relevant to the Ki-67 readouts of the wound healing model,[60,61] and that miR-425-5p was the only miRNA predicted from miRPathDB to mediate dysregulation of insulin signaling,[62,63] all of which highly relevant to the db/db model used here. Gene Ontology (GO) term analysis also provided additional candidate targets for miR-425-5p action, suggesting roles in regulating hypoxia inducible factor, cyclin-dependent kinase, and CD44 (Figure S4). Therefore, to better understand the relevance of a specific miRNA, we focused on a relevant *in vitro* model. Since we observed that myeloid cell types comprised the vast majority of cells in PVA sponges as identified by scRNA-seq (Figure 1), and that the collection of EVs from cultured primary macrophages yields biologically active EVs,[8] we used macrophage colony stimulating factor (M-CSF) to differentiate cells isolated from PVA sponge implants from WT vs. db/db mice from which EVs were then collected for further analysis (Figure 5E). Equivalent numbers of EVs were released into the conditioned media from db/db and WT macrophages (Figure 5F). These EVs were subjected to RT-qPCR to measure changes in the levels of miR-425-5p in EVs from db/db vs. WT macrophages and identified a significant decrease in miR-425-5p levels (15-fold decrease, $p < 0.0051$) in db/db EVs compared with WT mouse EVs (Figure 5G). These *in vitro* findings focused on EVs collected from cultured macrophages were consistent with *in vivo* miRNA-seq studies of PVA sponge-derived EVs, where both showed a down-regulation of miR-425-5p levels in db/db vs. WT EVs. Therefore, we next focused on identifying a biological activity of miR-425-5p-loaded EVs in the db/db wound model.

Functional activity of miR-425-5p-loaded EVs in diabetic wounds

Having shown that db/db EVs isolated from cultured macrophages have decreased levels of miR-425-5p compared with WT EVs (Figure 5G), we tested the *in vivo* biological activity of miR-425-5p-loaded EVs on wound healing compared with a negative control miRNA. We used cel-miR-67 as a negative control; it is derived from *C. elegans* and associated with minimal effects on eukaryotic cell signaling.[57,63,64] In addition, several control studies using fluorescently labeled miRNA controls were performed to optimize the concentrations for loading EVs with specific miRNAs and validating by vFC that the surface profile of canonical tetraspanins CD9 and CD63 were unchanged by treatment with Exofect reagent (Figure S5). We focused on using WT EVs for miRNA loading since, although WT EVs are known to be pro-reparative, our goal was to identify an miRNA payload that would substantially improve on the known pro-reparative activity of WT EVs from naive mice. Therefore, to determine whether treatment with miR-425-5p-loaded EVs would stimulate a pro-reparative phenotype in a wound healing assay, we treated full-thickness wounds in recipient db/db mice, observed the wounds over 14 days, and performed image analysis over 14 days (Figure 6A). We observed that treatment with miR-425-5p-loaded EVs significantly accelerated wound closure on day 7 compared with standard WT EVs from naive mice (no loading with any miRNA) (Figure 6B) ($p < 0.005$), consistent with the hypothesis that pro-reparative activity of WT EVs was increased by loading EVs with miR-425-5p. Additional controls comparing miR-425-5p-loaded EVs with negative control miRNA, mock-loaded EVs (i.e., treated with the Exofect reagent but lacking miRNA), or saline alone demonstrated the pro-reparative activity of miR-425-5p-loaded EVs in wound healing on days 7, 10, and 14 (Figure 6C) ($p < 0.0001$). Based on our observation that treatment with WT EVs led to an increased number of Ki67$^+$ basal keratinocytes in wound healing (Figure 4), we analyzed the effect of miR-425-5p-loaded EVs on cell proliferation in treated wounds as a molecular endpoint for the accelerated wound healing. We observed an increase in Ki67$^+$ basal keratinocytes (Figure 6D) that was quantified and statistically significant (5.2-fold increase; $p < 0.0024$) compared with a negative control miRNA mimic-loaded EVs (Figure 6E). Analysis of hematoxylin and eosin (H&E) histology after treatment with miR-425-5p-loaded EVs, negative control EVs, or saline controls further defined the effects of EV treatment (Figure 6F). Treatment with miR-425-5p-loaded EVs led to a statistically significant increase in the number of cells (Figure 6G, based on blue box on margins of Figure 6F; a 1.6-fold increase; $p < 0.0001$), but no significant changes in epidermal thickness (Figure 6H, centered on the wound bed), or collagen staining based on a Masson's trichrome stain (Figure 6I; ImageJ analysis of blue staining). We did observe an overall increase in the differentiation of the underlying dermis that was associated with increased vascularity based on H&E staining (Figures S6A and S6B). However, the density of CD31$^+$ blood vessels per high-powered field was similar between treatment with miR-425-5p-loaded EVs vs. Neg-miR-loaded EVs (Figures S6C and S6D). Therefore, the effects of treatment with miR-425-5p-loaded EVs on blood vessels in this model may be indirect, because it was associated with a robust pro-reparative phenotype or may be direct by promoting endothelial survival, as recently proposed.[60] Based on the translational relevance of our studies of miR-loaded EVs on wound healing, we next determined whether loading of EVs with miRNA using Exofect would lead to a significant amount of miRNA attached to the surface or would miRNAs be internalized and protected by the lipid membrane.

Figure 4. Adoptive transfer of EVs from diabetic obese donors have impaired wound healing activity

(A) Schematic of EV adoptive transfer strategy. (B and C) (B) Representative images of recipient wound beds on each day after treatment with saline control, WT EVs, or db/db EVs (a volume of 50 μL of EVs at 5–7 $\times$ 10^6 PVA EVs/μL) and (C) quantification of wound closure kinetics (n = 10 per group; ****$p < 0.0001$, **$p < 0.005$, *$p < 0.05$). (D) Representative H&E-stained wounds on day 14 after treatment with WT vs. db/db EVs (inset on right = high magnification). (E) Quantification of epidermis thickness (μm) based on H&E images indicated with brackets in (D). (F) Quantification of cell count per area (mm^2) based on H&E-stained images. (G) Localization of Ki67$^+$ cells by immunohistochemistry on day 14 after treatment with WT or db/db EVs. (H) Schematic of WT CD63 and CD63-FLAG tag (top) and vFC analysis of surface levels FLAG tag (bottom). (I) Immunoblotting to detect FLAG tag expression. (J) Localization of FLAG-tagged EVs in wound bed after a 24 h treatment with saline (top), CD63-expressing EVs (middle), or CD63-FLAG expressing EVs. (green line, edge; blue box, wound margin; red box, wound bed). High-magnification images of wound margin (middle) and wound bed (red) are shown. (K) Uptake of control CD63 (top) vs. CD63-FLAG-tagged (bottom) EVs into MEFs. (L) Quantification of FLAG-tagged EV uptake into MEFs.

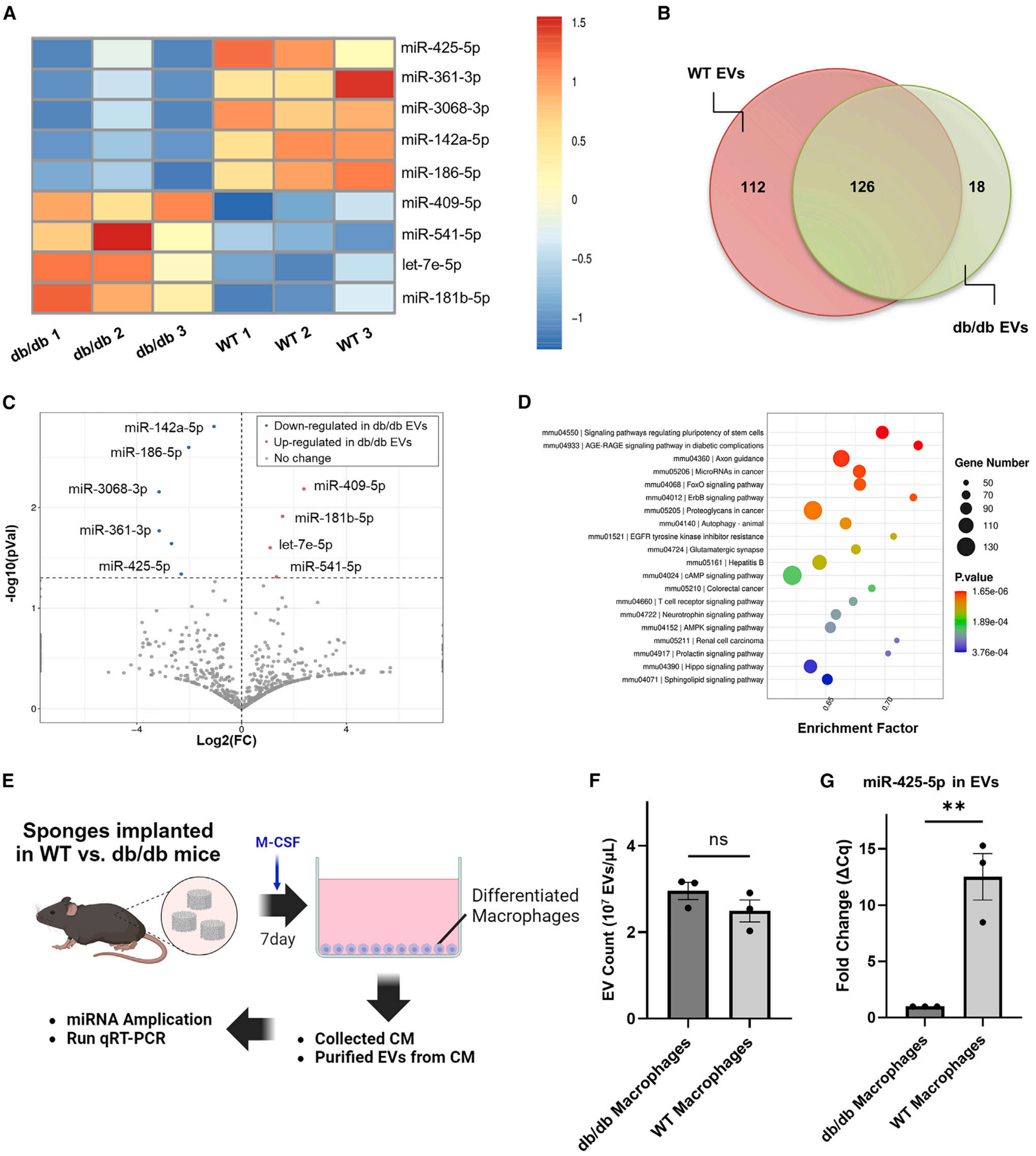

Figure 5. Regulation of miRNA EV payloads isolated in diabetic obese mouse model

(A) miRNA-seq analysis of fold changes in WT vs. db/db EVs. (B) Distribution of miRNA profile between WT and db/db EVs. (C) Analysis of fold change (FC) vs. significance (P) of miRNAs identified as described in the materials and methods. (D) KEGG pathway analysis of miRNAs identified. (E) Schematic of EV collection from cultured sponge-derived macrophages. (F) Quantification of EV yield from cultured macrophages. (G) RT-qPCR analysis of miR-425-5p in EVs isolated from the conditioned media from db/db vs. WT macrophages ($n = 3$ from each group. $p < 0.005$). ns, not significant.

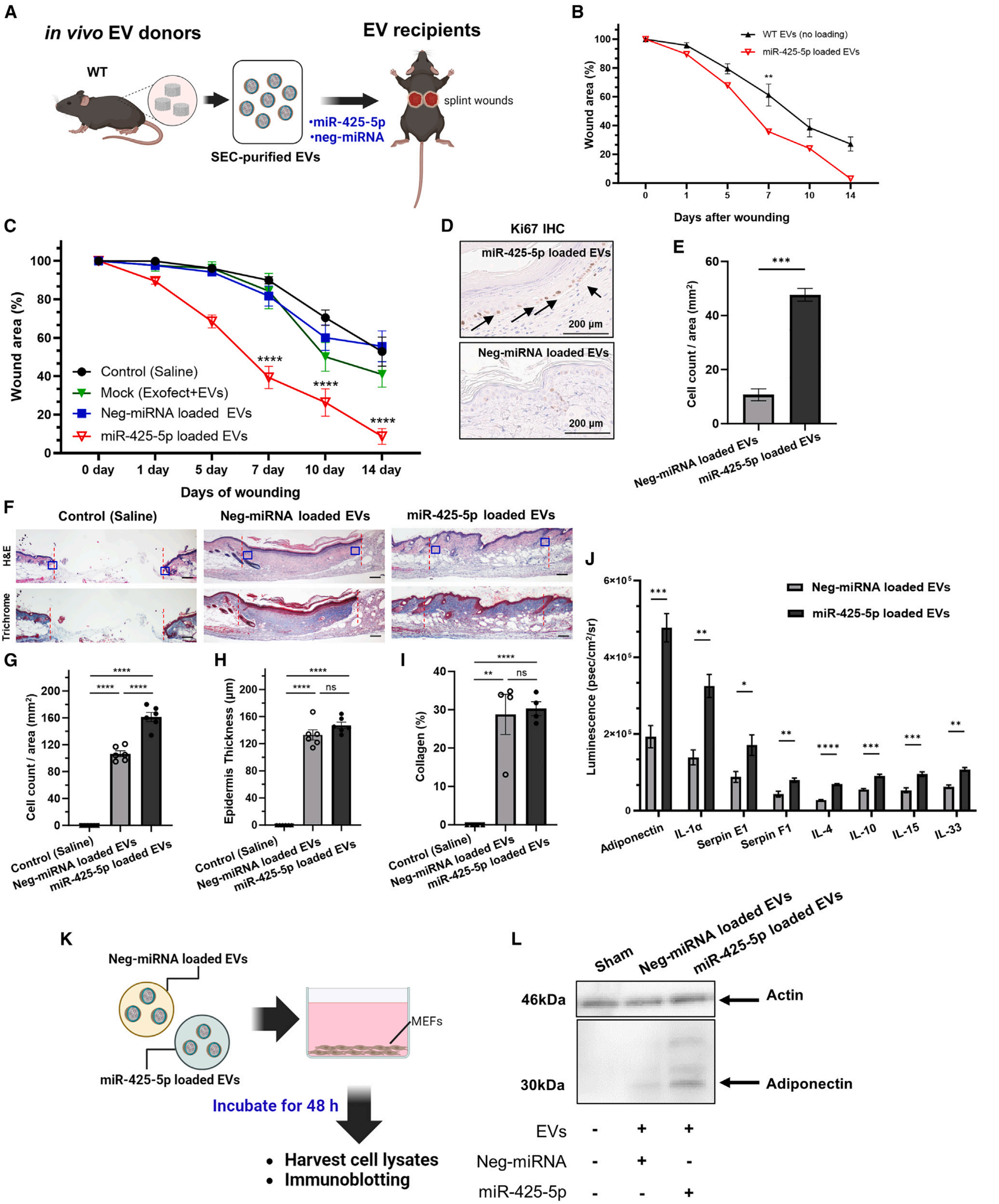

(legend on next page)

Therefore, we tested the effect of an *in vitro* nuclease treatment on isolated EVs using benzonase.[65] qRT-PCR was performed to measure changes in levels of miR-425-5p from vehicle vs. benzonase-treated EVs. We observed no substantial differences, consistent with miRNAs being present within the EV and thus protected from benzonase activity (Figure S7).

Based on the pro-reparative effect miR-425-5p-loaded EVs on wound healing (Figure 6C) associated with the increased proliferation of basal keratinocytes (Figures 6D and 6E), we focused on the identification of soluble mediators such as cytokines and adipokines that could mediate signaling between cell layers in the skin. Therefore, we performed a cytokine analysis testing a panel of 24 cytokines and adipokines to quantify relevant changes in inflammation mediators.[66] WCLs of wound tissue treated with miR-425-5p-loaded EVs were compared with tissues treated with negative control miRNA-loaded EVs as described above. We observed significant increases in several factors, including adiponectin (2.4-fold increase; $p < 0.0009$), IL-1α (2.3-fold increase; $p < 0.002$), and Serpin E1 (1.9-fold increase; $p < 0.037$), along with many other factors that were unchanged (Figure S8). Based on the relevance of adiponectin as an important mediator of glucose metabolism in diabetic obese models,[67,68] the uptake of FLAG-tagged EVs into fibroblasts (Figure 4), and the general abundance of fibroblasts in the wound bed, we focused on testing whether macrophage-derived EVs containing miR-425-5p (Figure 5G) could stimulate fibroblasts *in vitro*. Therefore, we assessed whether the treatment of MEFs with miR-425-5p-loaded EVs would stimulate adiponectin expression *in vitro* (Figure 6K), as predicted from *in vivo* treatments with miR-425-5p-loaded EVs stimulating adiponectin expression (Figure 6J). We subjected cultures of MEFs to 48 h treatment with miR-425-5p-loaded EVs compared with negative control miRNA-loaded EVs as prepared for the *in vivo* studies above. We observed by immunoblotting of WCLs that miR-425-5p-loaded EVs led to a 1.4-fold increase in adiponectin expression compared with treatment of MEFs with control EVs (Figure 6L), using actin levels as a loading control. Although it remains unclear whether miR-425-5p has a direct effect on the adiponectin mRNA based on predicted binding sites of miR-425-5p, these findings suggest that treatment of wounds *in vivo* or MEFs *in vitro* with miR-425-5p-loaded EVs leads to increases in adiponectin expression. In a model where macrophage-derived EVs promote intercellular signaling such as the release of EVs from macrophages that stimulate fibroblasts, defining cell type-specific EV release with a genetic tool would provide important insights.

Cell type-specific tracking of EV release and uptake

To address the question of cell type-specific EV release, we used a Cre-Lox system for the regulated expression of a fluorescent EV reporter, the tetraspanin CD9 as a fusion to GFP. Cell profiling of PVA sponge donor site using scRNA-seq showed high numbers of macrophages and related cell types (Figure 1), while immunohistochemistry of full-thickness wounds showed high numbers of F4/80$^+$ macrophages distributed in the adipose layer (Figure S9). Therefore, we selected transgenic mice that expressed the Cre recombinase in macrophages for comparison with transgenic mice expressing Cre in other skin-relevant cell types such as endothelium (TEK) and keratinocytes (KRT14). Each tissue-specific transgenic Cre mouse line was crossed with mice expressing the CD9:GFP, termed the TIGER reporter (transgenic inducible GFP EV reporter) (Figure 7A).[24] Tissue-specific expression of CD9-GFP was under the control of an upstream lox-STOP-lox cassette and crossed with transgenic mice expressing Cre under the control of the LysM promoter (LysM-Cre) to assess the expression and release of GFP$^+$ EVs from myeloid immune cells like macrophages and monocytes. Crosses with TEK-Cre and CD9-GFP mice were performed in parallel with crosses with TEK-Cre × CD9:GFP and KRT14-Cre × CD9-GFP. We first established the expression of GFP in each of the transgenic mouse lines by analyzing cells recruited into the PVA sponge implants in parallel with the collection of donor EVs as described above (Figure 7B). In addition, laser scanning confocal microscopy of PVA sponge implants was performed for each genotype to establish positive controls for each of the mouse models (i.e., TEK-CD9-GFP, LysM-CD9-GFP, and KRT14-CD9-GFP) (Figure S10). For each donor genotype, cells (Figure 7B) and EVs (Figure 7C) were purified from the PVA sponge implants as described above. Standard cell flow cytometry was performed for cells collected where we observed that LysM-CD9-GFP and TEK-CD9-GFP mice expressed GFP. Since the surgical placement of PVA sponge implants was between the adipose and dermal layers, few KRT14$^+$ cells migrated or infiltrated the PVA sponge implant in KRT14-CD9-GFP mice (Figures 7B and S10), although KRT14$^+$ keratinocytes were present in intact overlying skin (data not shown). Analysis of EVs purified from PVA sponge implants of each genotype focused on LysM-CD9-GFP mice that released high numbers of bright GFP$^+$ EVs. In contrast, there were few TEK-CD9-GFP$^+$ or KRT14-CD9-GFP EVs (Figure 7C).

Figure 6. Biological testing of miR-425-5p-loaded EVs

(A) Schematic of EV collection, loading with specific miRNAs *in vitro*, followed by treatment of splinted wounds *in vivo* to assess miR-425-5p biological activity. (B) Comparison of wound closure kinetics of normal WT EVs (i.e., no miRNA loading) vs. WT EVs loaded with miR-425-5p to show that EVs loaded with miR-425-5p are more pro-reparative than WT EVs. (C) Analysis of wound closure kinetics with controls, including saline (black), mock-treated EVs (i.e., EVs treated with Exofect reagent without miRNA; green), negative control miRNA-loaded EVs (i.e., cel-miR-67; blue), or miR-425-5p-loaded EVs (red) (a volume of 50 μL of EVs at 5–7 × 10^6 PVA EVs/μL). (D and E) (D) Localization and (E) quantification of Ki67$^+$ cells in the basal epidermal skin layer to assess the general pro-reparative effects of miR-425-5p-loaded EV treatment. (F–I) (F) Representative H&E (top) and Masson's trichrome stained images (bottom) on day 14 after treatment with saline, neg-miRNA-loaded EVs or miR-425-5p-loaded EVs (red dotted line = wound margin; blue box = area used for cell counts and epidermis thickness measurements). Quantification of the effects of treatment with neg-miRNA-loaded EVs vs. miR-425-5p-loaded EVs on (G) cell count, (H) epidermis thickness, and (I) collagen as a percentage of the dermis area (mm^2) based on Masson's trichrome staining. (J) Protein panel quantifying changes in adipokine/cytokine expression in wound beds treated with miR-425-5p-loaded EVs vs. negative control miRNA-loaded EVs at 14 days (n = 2 for each treatment group). Figure S8 has the complete profile. (K) Schematic of *in vitro* of PVA sponge-derived EVs loaded with negative control miRNA vs. miR-425-5p used to treat MEFs. (L) Immunoblotting of Adiponectin protein of MEFs treated with control vs. miR-425-5p-loaded EVs. (****$p < 0.0001$, ***$p < 0.001$, **$p < 0.005$, *$p < 0.05$).

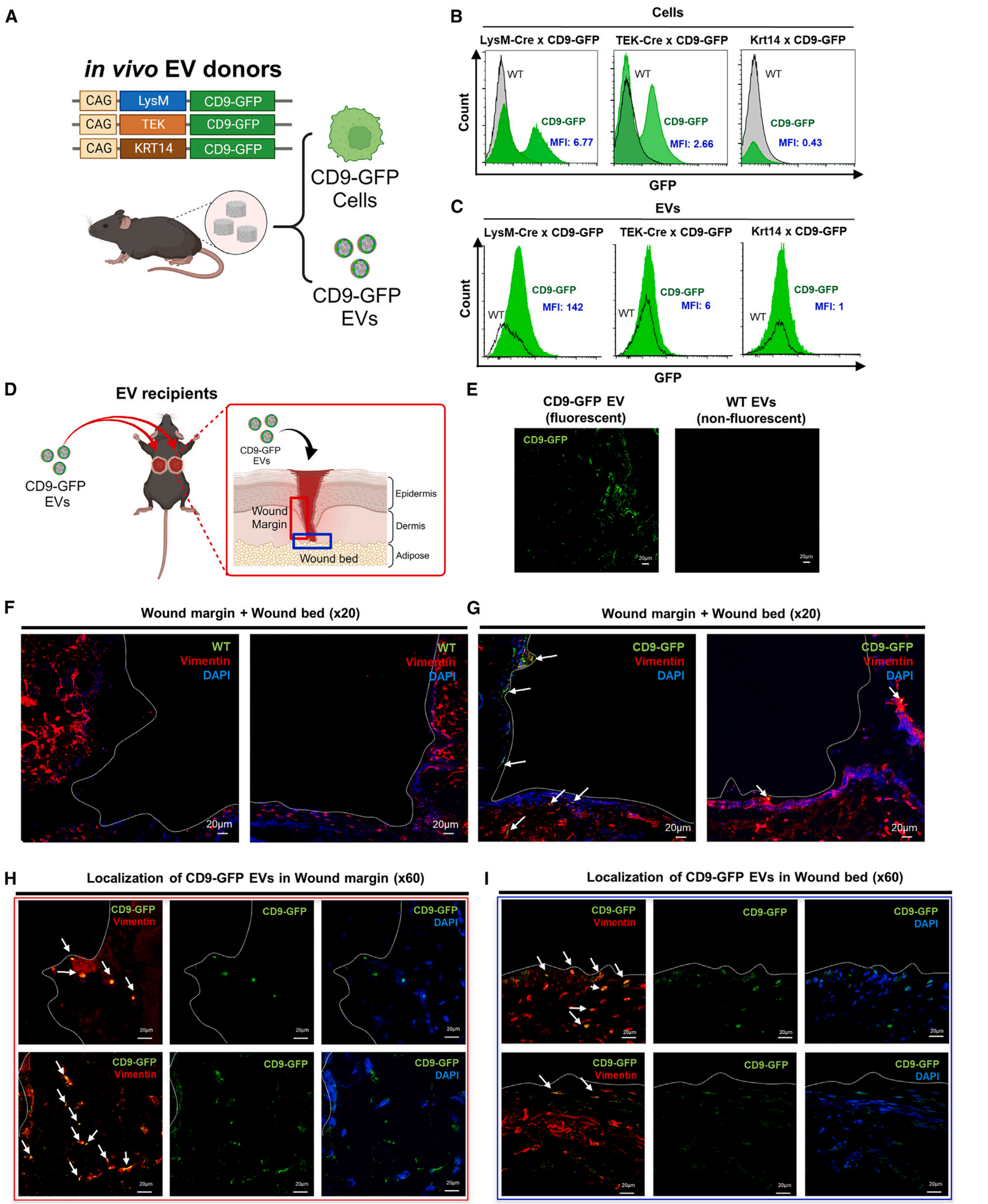

(legend on next page)

We proceeded to further assess the distribution of macrophage-derived EVs (i.e., LysM-CD9-GFP EVs) based on their abundance relative to other cell type-specific promoters tested (Figure 7B) by treatment of full-thickness wounds of db/db mice (Figure 7D). We observed that GFP$^+$ EVs or accumulations of GFP$^+$ EVs could be observed by confocal microscopy in wounds treated with LysM-CD9-GFP EVs compared with the lack of signal in images of wounds treated with non-fluorescent control EVs isolated from sibling-matched controls lacking the Cre driver genes (Figure 7E). To determine whether LysM-CD9-GFP EVs could be localized in fibroblasts based on a model of EV uptake by fibroblasts in the wound bed, we first established the distribution of fibroblasts in the wound site by immunostaining full-thickness wound sites with an anti-vimentin antibody (Figure 7F). Imaging of both the left and right sides of the wound show the distribution of vimentin$^+$ fibroblasts on the wound margin (Figure 7F, sides of the images on the left and right) and the wound bed (Figure 7F, bottoms of the images on the left and right). These low-magnification images provide landmarks for the wound margins (i.e., sides of the wound) vs. the wound bed (i.e., bottom of the wound), and the negative control images for wounds treated with WT non-fluorescent EVs (Figure 7F) vs. wounds treated with LysM-CD9-GFP EVs (Figure 7G). These representative low-magnification images (Figures 7F and 7G) were further analyzed at higher magnification to localize the accumulation of LysM-CD9-GFP EVs in replicate high power fields of the wound margin (Figure 7H, based on red box corresponding with Figure 7D), and in the wound bed (Figure 7I, based on blue boxes corresponding with Figure 7D). These imaging studies established that populations of LysM-CD9-GFP EVs were co-localized with vimentin$^+$ fibroblasts in the wound margin (Figure 7H) and in the wound bed (Figure 7I). While these co-localization analyses did not exclude the possibility of CD9-GFP uptake into other cell types, these studies provided insights into the distribution of EV uptake in a complex microenvironment. Furthermore, EVs purified from a specific cell type such as macrophages express surface proteins that may be relevant to their tropism and activity.

DISCUSSION

The healthy wound healing response is characterized by coordinated phases of hemostasis, inflammation, proliferation, and remodeling; however, many aspects of the molecular and cellular basis of this response remain poorly understood. The dysregulation of the coordinated response that is associated with impaired wound healing has led us to identify defects in intercellular signaling between cell layers in the skin with a focus on EVs and their payloads as mediators of these processes. We propose that macrophage-derived EVs from resident macrophages have biologically active payloads that are internalized by skin fibroblasts to stimulate signaling, and specific adipokine expression that leads to a pro-reparative response that includes proliferation of overlying keratinocytes.

The composition and function of EVs released into biological fluids and cultured media, which are heterogeneous in origin, depends on the cells that produce them. There are few *in vivo* models that have mapped the landscape of cells and EVs in a well-defined microenvironment with genetic tools. Here, we focused on wound healing in db/db mice as a genetic model of impaired wound healing to show that EVs enriched from db/db donors have impaired wound healing activity and a decreased capacity to signal specific molecular endpoints in fibroblasts that was associated with stimulating the proliferation of basal layer epithelial cells. miRNA-seq of EVs isolated from db/db vs. WT mouse donors revealed a decrease in the miR-425-5p. miR-425-5p, especially when loaded into EVs, has been associated with dysregulated insulin signaling in some models[61] or as a pro-survival endothelial factor in db/db EVs in other models.[60] We show that miR-425-5p was differentially expressed in macrophage-derived EVs isolated from db/db vs. WT mice and that wounds treated with miR-425-5p-loaded EVs promoted wound closure; we also identified a miR-425-5p-mediated upregulation of adiponectin in the wound bed *in vivo* and in cultured fibroblasts *in vitro*. Based on our findings that macrophage-derived EVs signal to other cell types in the wound, we used a cell type-specific CD9-GFP reporter model to define the distribution and map uptake of macrophage-derived EVs into fibroblasts. Together, these studies defined a population of macrophage-derived EVs that are internalized by dermal fibroblasts to regulate adiponectin expression associated with promoting wound healing and cell proliferation.

Recent work from our lab has focused on the identification of quantitative changes in protein payloads in EVs isolated from db/db vs. WT donor mouse models with a goal of engineering EVs to deliver these pro-reparative payloads to the wound bed. For example, we identified a down-regulation of proteins associated with extracellular matrix remodeling and innate immunity[8] and re-expressed select serine protease inhibitors to reverse the impaired wound healing phenotype of db/db recipients. Here we focused on miRNA analyses of db/db vs. WT EV payloads and identified several miRNAs that were down-regulated in db/db EVs and others that were up-regulated. To establish the function of a specific miRNA as an example of how to validate the activity of candidate miRNA, we selected miR-425-5p for testing, along with negative controls for activity and positive controls for EV loading. While several miRNA profiling studies have identified

Figure 7. Transgenic mice expressing the tetraspanin CD9-GFP to assess cell type-specific EV release and track EV uptake

(A) Schematic of lineage mapping mouse lines using CD9-GFP TIGER model and cell type-specific expression of Cre. (B and C) (B) Analysis of GFP expression in cells and (C) EVs from PVA sponge implants from transgenic mice expressing CD9-GFP under the control of LysM, TEK, or Krt14 promoters, as detailed in the materials and methods. (D) Schematic of adoptive transfer of CD9-GFP EVs into splinted wounds of db/db mice. (A volume of 50 μL EVs at 5–7 × 10^6 PVA EVs/μL). (E–G) (E) Detection of CD9-GFP$^+$ EVs (left; green fluorescence) vs. WT EVs (right; non-fluorescent EVs from CD9-GFP$^-$ mice) in the wound bed. Representative low magnification images of (F) WT non-fluorescent EVs vs. (G) CD9-GFP$^+$ EVs (white arrowheads) in the splinted wound (left and right images comprise the full wound site), counterstained with vimentin for fibroblasts (red) and DAPI for nuclei (blue).

(H and I) (H) Representative high magnification images of CD9-GFP$^+$ EVs from two different fields (top and bottom) detected in the wound margin (based on red box from D) and (I) wound bed (based on blue box from D). CD9-GFP$^+$ EVs colocalized with vimentin indicated with white arrows.

miRNAs that are relevant in impaired wound healing, comparatively fewer have assessed the activity of specific EV-loaded miRNAs in wound healing.[69] Therefore, we used a combination of target pathway analysis databases and literature review to prioritize specific miRNAs for functional testing. While our studies were limited to the identification of miRNAs dysregulated in the db/db PVA sponge model, further studies with antagomirs, miRNA knockout mice, and miRNA activity reporter tools will be important to better understand loss-of-function phenotypes for specific miRNAs.[70–73] One of the limitations of functional testing of endogenous miRNA-loaded EVs in the field is the poorly understood nature of miRNA abundance and distribution in a population of EVs.[74,75] For our analysis of miRNA activity, we focused on testing an miRNA that was down-regulated in db/db EVs that could then be delivered in an EV to restore a pathway(s) in a wound bed treated with EVs loaded with that down-regulated miRNA. To this end, we identified miR-425-5p as one of the most relevant miRNAs based on the obese hyperglycemic phenotype of the db/db mice from which EVs were collected and analyzed by miRNA-seq, and reports that linked miR-425-5p action to the regulation of insulin responsiveness.[62,63] We suggest that miR-425-5p regulated insulin signaling may be linked to the miR-425-5p-mediated changes in the expression of an adipokine-like adiponectin in the regulation of glucose sensitivity.[76] We proposed that miR-425-5p-loaded EVs may have a role in stimulating secreted factors in adjacent cell types that promote wound healing. In addition to adiponectin, future studies may focus on other candidates such as IL-1α and Serpin E1, also known as plasminogen activating inhibitor, that are relevant in inflammation and angiogenesis, respectively. Therefore, with recent studies identifying an activity for miR-425-5p in endothelial cells as a pro-survival regulator of endothelial cells that promotes wound healing in a streptozotocin model of impaired diabetic wound healing,[60] we examined the effects of miR-425-5p-loaded EVs in a genetic db/db model. Although we did not observe a significant difference in CD31$^+$ blood vessel density, systematic approaches that test miRNA action in complex tissues will be necessary to better define cell type-specific effects of miRNAs.

With a significant interest of the EV field focused on how to define EV source and uptake, we used an *in vivo* model where the EV source is defined by cell types relevant to cutaneous wound healing, like macrophages. We show that macrophage-derived EVs can be tracked and purified for adoptive transfer studies using cell type-specific promoters to follow GFP fluorescence by flow cytometry and microscopy. Fluorescent reporter systems utilizing CD9-GFP fusions,[24] as we have done for cutaneous injury models, along with recent studies using fusion reporters with CD81 for EV tracking from blood, brain, liver, and ovary,[26] provide important insights into the relevant cell types and biodistribution *in vivo*. The development of pH-dependent fluorescent EV reporters that distinguish between EVs in acidic late endosomal MVBs vs. the release of EVs in neutral extracellular space and for tracking EV uptake provides further support for the utility of tetraspanin:fluorescent reporters in the understanding of EV release and uptake.[77] These tetraspanin:fluorescent reporters are important tools to understand the biodistribution and activity of nucleic acid payloads like functional miRNAs and guide RNAs that direct CRISPR-Cas9 machinery for gene editing target cells.[78] We have also shown here that direct tagging of tetraspanins, like the FLAG tagging of CD63 on EVs takes advantage of a well-established molecular tool that also has utility in purification strategies.[79]

The overall novelty of our studies is that we establish an *in vivo* system for the efficient and high-yield purification of EVs that can be applied to various animal models to define the biological activity and assess the molecular endpoints of engineered EVs. These engineered EVs can deliver pro-reparative payloads identified by -omic approaches that are most relevant in accelerating the resolution of inflammation and promoting the proliferation of specific cell types relevant in tissue repair. With the application of fluorescent, genetic, and other advanced EV tracking technologies, lineage mapping of the source of EV release and uptake of EVs into recipient cells can lead to a molecular understanding of intercellular signaling mediated by EVs between skin layers in wound healing.

MATERIALS AND METHODS

Mouse model for EV collection from PVA sponges

All mouse studies were conducted in accordance with the Institutional Animal Care and Use Committee of the University of California San Diego. We used 12- to 16-week-old WT and db/db mice (B6.BKS(D)-Lepr$^{db/db}$/J; The Jackson Laboratory #000697, Bar Harbor, ME, USA), where db/db mice had a blood glucose level of >300 mg/dL and body weight >45 g, the criteria for the diabetic obese model.[80,81] Mice were prepared for the subcutaneous implantation of three PVA sponges (Cat# SQ5000, PVA Unlimited Inc., Warsaw, IN, USA) by shaving and topical treatment with depilatory cream of dorsal skin. After PVA sponge implantation, skin was closed with nylon monofilament sutures and incubated for 7 days. PVA sponges were then harvested by direct transfer of all three sponges into 1 mL PBS for the recovery of cells infiltrating the sponges and associated fluid flushed from the sponges that contained EVs. Centrifugation at 3,000$\times g$ for 5 min separated cells into a pellet that was used for scRNA-seq, while the supernatant of the PVA sponge fluid contained EVs for further analysis.

For the goal of identifying cell type-specific sources of EVs, we used transgenic mice expressing a CD9-TurboGFP reporter targeted to EVs under the control of a lox-STOP-lox cassette to facilitate cell type-specific expression based on promoters driving the expression of Cre in specific cell types, termed TIGER knock-in mice.[24] The following three crosses were performed with these CD9-GFP mice (B6; 129S1-Gt(ROSA)26Sor$^{tm1(CAG-CD9/GFP)Dmfel}$/J; Jackson Laboratories #033361). For expression of the CD9-GFP reporter in macrophages/monocytes mice were crossed with a LysM-Cre mice (B6.129P2-Lyz2$^{tm1(cre)Ifo}$/J; Jackson Laboratories #004781). For the expression of the CD9-GFP reporter in endothelial cells, mice were crossed with TEK-Cre mice (B6.Cg-Tg(TEK-cre)12Flv/J; Jackson Laboratories #004128). For the expression of CD9-GFP reporter in keratinocytes, mice were crossed with KRT14-Cre mice (B6N.Cg-Tg(KRT14-cre)1Amc/J; Jackson Laboratories #018964) (Table S2).

scRNA-seq

scRNA-seq was performed on cells recovered from PVA sponges implanted into each of 3 different WT mice that were then pooled and compared with similarly pooled cells from each of three different db/db mice. scRNA-seq data were archived at NCBI (GSE242497). In brief, 1×10^5 cells were collected from the PVA sponges from each mouse, pooled and then 1×10^4 cells were loaded on the 10× Chromium Next GEM using the Single Cell 3′ Reagent (v3.1) with gel beads and master mix for cell capture and GEM generation (Cat # 1000147, 10× Genomics, San Francisco, CA, USA). Subsequently, samples underwent GEM reverse transcription cleanup, cDNA amplification, and 3′ gene expression library construction according to the manufacturer's instructions (10× Genomics). Constructed libraries were then sequenced on HiSeq sequencers (Illumina, San Diego, CA, USA) using paired end reads at the University of California, San Diego Institute for Genomic Medicine (Tables S3 and S7). scRNA-seq data were demultiplexed, giving rise to 2 FASTQ files per sample (4 FASTQ files in total), and aligned to the reference murine genome GRCm38 (mm10, v2020-A) into single cells using the Cell Ranger Count pipeline (10× Genomics, v7.0.0) with the following settings for each sample, independently: library type, single cell 3′ gene expression; check library compatibility, true; chemistry, auto; include introns, true; no_bam, false; and no secondary analysis, false. Cell Ranger Count outputs for each sample were then aggregated and normalized into a single gene expression matrix using the Cell Ranger Aggr pipeline (10× Genomics, v7.0.0) with the follow settings: no secondary analysis, false; and normalization mode, mapped. Running Cell Ranger Aggr yielded approximately 18,000 post-normalization mean reads per cell. Further data filtering and analysis were conducted using Loupe Browser (10× Genomics, v6.1.0). Quality control included omitting cells with >15% mitochondrial UMIs per barcode (linear) or <9.185 Genes per Barcode (Log2); cells that passed these quality control filters were included in downstream analysis. The top 10 principal components were used for graph-based clustering, and the following settings were applied for dimensionality reduction via uniform manifold approximation and projection (UMAP) analysis: minimum distance, 0.1; and number of neighbors, 15.

EV isolation and analysis

EV studies addressed the methodological recommendations of the Minimal Information for Studies of Extracellular Vesicles 2018,[82] including nomenclature, collection/pre-processing, EV separation/concentration, EV characterization, functional studies, and reporting that are all archived at EV-TRACK (evtrack.org; #EV230979). For the isolation of EVs from PVA sponge implants, the cell-free supernatant was subjected to two 10,000×*g* spins for 30 min at 4°C followed by SEC (Cat # ICI-70, IZON, Medford, MA, USA) and the collection of 22 fractions of 700 μL each. EVs from cultured cell media were enriched using Exoquick reagent (Cat # EQULTRA-20A-1, System Biosciences, Palo Alto, CA, USA) following the manufacturer's protocol.

Single vFC

EV concentration, size, and analysis of surface proteins and fluorescent proteins were measured by single vFC using a commercial assay based on a fluorescent lipophilic membrane dye, vFRed (vFC Assay kit, Cat # CBS4HP-1PE, Cellarcus Biosciences, San Diego, CA, USA), using a CytoFLEX flow cytometer (Model S, V4-B2-Y4-R3, Beckman Coulter, Indianapolis, IN, USA) (Table S7). The flow cytometer was calibrated for vesicle size and IF using fluorescent intensity standard beads (nanoRainbow, Cellarcus) and antibody capture beads (nanoCal, Cellarcus),[83–85] and showed a size (diameter) limit of detection (LOD) of ∼80 nm and an IF LOD of ∼25 PE (phycoerythrin) molecules of equivalent soluble fluorochrome. Samples were diluted (optimal dilution determined in preliminary experiments), stained with vFRed and PE-conjugated antibodies (Tables S5 and S6), subjected to a 1,000-fold post-stain dilution, and 100 μL measured on the flow cytometer at a flow rate of 60 μL/min. Data were analyzed using FCS Express (Dotmatics/De Novo Software, Pasadena, CA, USA) and a standardized layout used to apply gating, compensation, and calibration (Cellarcus). Single vFC data were archived at flowcytometry.org (ID: FR-FCM-Z749) with a MIFlowCyt score of 95%.

EV characterizations by immunoblotting, electron microscopy, and multiplex analysis

Lysates of EVs isolated from PVA sponges, paired along with WCLs from the sponge implants, were prepared in RIPA lysis buffer. Loading of WCLs were normalized by protein quantification with a BCA assay (Cat# 23225, Thermo Fisher Scientific, Carlsbad, CA, USA) while loading of EVs was normalized to EV counts based on vFC analysis as described above. Nonfat Dry Milk (Cell Signaling Technology, Denver, MA, USA) was used for blocking in Tris-buffered saline with 0.05% Tween 20 and primary antibodies incubated overnight at 4 °C. Table S4 details primary and secondary antibodies used for immunoblotting. Immunoblots were detected with horseradish peroxidase-conjugated secondary, incubated with enhanced chemiluminescent reagent (Cat# 32209, Thermo Fisher Scientific) and detected with an IVIS-Lumina Imager (PerkinElmer, Shelton, CT, USA). For imaging of EVs by transmission electronic microscopy, EV samples were applied onto electronic microscopy grids, washed, and stained with uranyl acetate and images obtained with a Jeol 1400 plus transmission electron microscope at 80 keV. For the multiplex analysis of EVs present on EVs, a bead-based screen for 37 EV surface proteins was used (MACSPlex Exosome Kit, Cat#130-122-211, Miltenyi-Biotec, San Diego, CA, USA), and followed the manufacturer's recommendations. Data were analyzed with data analysis template using MACSQuant (ver. 2.12.2) (Miltenyi-Biotec).

EVs studies to assess wound healing, signaling, and uptake *in vivo*

To assess the activity of EVs upon the kinetics of wound closure, EVs were purified from PVA sponge implants as described above and used to treat full-thickness, splinted, 4-mm wounds as previously described.[86,87] Briefly, a silicone ring (Cat# GBLRD476687, Grace Bio-labs, Bend, OR, USA) was immobilized with 4-0 nylon sutures (Cat # 50-118-0628, Thermo Fisher Scientific) around each wound and immediately treated with 5–7 × 10^6 EV in a volume of 50 μL PBS per wound and covered with 3M Tegaderm (Cat #, 264435,

Mckesson, Irving, TX, USA).[86] Wounds were imaged with a Galaxy S10e (1200 pixels, AF, F1.5/F2.4 super speed dual pixel, Samsung, Suwon, South Korea) and analyzed by ImageJ (1.53e version). Tissues were harvested for histology analysis by fixation of skin wound samples in paraformaldehyde into paraffin at the University of California San Diego Tissue Technology Shared Resource (TTSR) that prepared slides stained with hematoxylin and eosin and Masson's trichrome stains, to detect collagen staining in blue. Immunohistochemical staining to localize Ki-67 (1:50; Cat #,16667, Genetex, Irvine, CA, USA) immunohistochemical staining was performed with a Intellipath Automated IHC Stainer (Biocare, Pacheco, CA, USA) by the TTSR, while immunostaining with anti-FLAG antibody (1:100; Cat # 14793, Cell Signaling Technologies) and anti-F4/80 (1:100, Cat# 70076, Cell Signaling Technologies) was performed with HRP detection SignalBoost reagents (Cat# 8114 and 8059, Cell Signaling Technologies). Tissues harvest for analysis for cytokines/adipokines was performed with a Proteome Profiler (Cat # ARY028, R&D Systems, Minneapolis, MN, USA) and followed manufacturers recommendations for detection and quantification with an IVIS-Lumina imaging system. Analysis of wounds treated with CD9-GFP^{+} EVs was performed by cryosectioning treated wounds with a cryostat (Model CM1850, Leica, Davisburg, MI, USA), imaged with a Nikon Confocal microscope (Model AXR, Tokyo, Japan). Counterstaining of CD9-GFP^{+} EVs was performed with an anti-vimentin antibody (1:200, Cat # 5741, Cell Signaling Technologies) to localize fibroblasts and detected with an Alexa 546 secondary antibody (1:1,000, Cat #A11010, Thermo Fisher Scientific). All H&E, Masson's trichrome and IF images were analyzed with ImageJ software.

Generation and testing of FLAG-tagged EVs

An epitope-tagged variant of CD63 was created by cloning the FLAG sequence (DYKDDDK) at amino acid 1397 on the second extracellular loop of CD63. Following transient transfection of HEK293T cells (Cat # CRL-1573, ATCC, Manassas, VA, USA) with Lipofectamine 3000 (Thermo Fisher Scientific) per the manufacturer's directions, the expression on the cell surface was validated by immunoblot with an anti-FLAG antibody (Cat# F-1804, Sigma, St. Louis, MO, USA). vFC was performed to detect expression of FLAG-tagged CD63 on EVs isolated from the conditioned media of transfected cells using a PE-conjugated anti-FLAG antibody (Cat# CBS18-PE-100T, Cellarcus Biosciences, San Diego, CA, USA). Uptake of FLAG-tagged EVs into primary MEFs was assessed by treatment of 1e^{6} cultured primary MEFs (Cat# SCRC-1008, ATCC) with 2×10^{9} EV in a volume of 100 μL, incubated for 48 h, fixed, and stained with an anti-FLAG tag antibody (1:100, Cat#14793, Cell Signaling Technologies) and a fluorescent anti-Rabbit Alex Fluor 488 (Cat# A-11008, Thermo Fisher Scientific).

In vitro analysis of macrophage-derived EV miRNAs and adiponectin expression

Primary macrophages prepared from PVA sponges were cultured for the collection of macrophage-derived EVs to measure levels of miR-425-5p. Briefly, PVA sponges were implanted into mice, incubated for 7 days, and cells were harvested as described above. Cells were cultured in RPMI media supplemented with 10% fetal bovine serum and 25 ng/mL M-CSF (Cat # 14-8983-80, Life Technologies, Carlsbad, CA, USA) for 7 days, with a media change at 3 days. Flow cytometry was performed on cells incubated for 7 days in M-CSF with an anti-F4/80 antibody, which was used to verify >90% F4/80^{+} cells. After the 7-day incubation, cells were transferred to RPMI media supplemented with 10% exosome-depleted FBS (SBI) and conditioned media collected after an additional 7 days of incubation. From this conditioned media, EVs were harvested using Exoquick per manufacturer's recommendation, and EVs analyzed for qPCR for miR-425-5p, as described below. The activity of miR-425-5p-loaded EVs upon the stimulation of MEFs was determined by incubation of EVs for 48 h, then measured by immunoblotting for adiponectin with an anti-adiponectin antibody (Cat #MA1-054, Thermo Fisher Scientific) and followed by detection with an IVIS-Lumina Imaging system.

miRNA sequencing, analysis, EV loading, and RT-PCR

For the analysis of miRNA EV payloads, EVs were purified by SEC from 7-day PVA sponge implants of WT vs. db/db mice. To obtain a more concentrated sample for RNA extraction, purified EVs were subjected to ultracentrifugation (259,000×*g*, for 70 min, Beckman Optima, Rotor TLA 120.2, k-factor 8), the supernatant discarded, and the pellet extracted to obtain RNA using Trizol (Thermo Fisher Scientific). RNA quality and quantity were analyzed with a Bioanalyzer 2100 (Agilent, Santa Clara, CA, USA), and 1 μg RNA used to prepare a small RNA library using a TruSeq Small RNA Sample Prep Kit (Illumina, San Diego, CA, USA). Single-end 50-bp sequencing was performed on an Illumina HiSeq 4000 (LC Sciences, Houston, TX, USA) with initial processing of raw reads using proprietary software ACGT101-miR (LC Sciences) to remove adapter dimers, foreign sequences, low-complexity fragments, common RNA families (e.g., rRNA, tRNA, small nuclear RNA, and small nucleolar RNA), and repetitive sequences. Sequences were mapped against species-specific miRNA precursor sequences available in miRBase 21.0 using NCBI BLAST to identify known and potentially novel miRNAs, with the alignment process allowing for length variation at both the 3′ and 5′ ends of the sequence and a tolerance for one mismatch within the sequence. Identification of known miRNAs involved recognition of unique sequences aligned with the mature miRNAs of a specific species located on the hairpin arm. At the same time, sequences aligning with the opposite arm of a known species-specific precursor hairpin without an annotated mature miRNA were classified as candidates for new 5p- or 3p-derived miRNAs. Unmapped sequences were aligned against the precursor sequences of selected species (excluding certain species) within miRBase 21.0 and subjected to further analysis. These mapped pre-miRNAs were then cross-referenced with the genome of a specific species to confirm their genomic location and classified as known miRNAs. The remaining unmapped sequences were subjected to BLAST searches against the genomes of specific species. Hairpin RNA structures containing these sequences were predicted using RNA fold software (http://rna.tbi.univie.ac.at/cgi-bin/RNAWebSuite/RNAfold.cgi) using the 80 nucleotides flanking the sequence.[88–90]
For the analysis of differentially expressed miRNAs, a normalization based on deep sequencing counts. To predict the genes targeted by most abundant miRNAs, two computational target prediction

algorithms (TargetScan 50 and Miranda 3.3a) were used to identify miRNA binding sites. Finally, the data predicted by both algorithms were combined and the overlaps were calculated. The GO terms and KEGG Pathway of these most abundant miRNAs, miRNA targets were also annotated. For loading miRNAs into EVs isolated from WT PVA sponge donors, 100 μL EVs at a concentration of 6×10^6 PVA EVs/μL were mixed with 200 pmol miR-425-5p (Cat# C-310988-01-0050, Horizon, San Diego, CA, USA) or a negative control cel-miR-67 (Cat# CN-001000-01-50, Horizon) in a volume of 200 μL following the manufacturer's recommendations for Exofect kit (Cat# EXFT20A-1, System Biosciences, SBI, Palo Alto, CA, USA). Conditions for EV loading with miRNAs were optimized with a Cy3-labeled miR control (SBI). miR-loaded EVs and followed manufacturer's recommendations for purification. RT-qPCR was performed on CFX96 (Bio-Rad) using the TaqMan Fast Advanced Master Mix for qPCR (Cat# 4444556, Thermo Fisher Scientific) and TaqMan Advanced miRNA Assay (mmu481161_mir) (Cat# A25576, Thermo Fisher Scientific). To confirm the expression of miR-425-5p in EVs collected from M-CSF-differentiated PVA macrophages, we extracted RNA using a mirVana miRNA Isolation Kit (Cat#AM1560, Thermo Fisher Scientific) following the manufacturer's protocol. cDNA was synthesized for each sample using the TaqMan Advanced miRNA cDNA Synthesis Kit (Cat# A28007, Thermo Fisher Scientific) and qPCR performed with a TaqMan Fast Advanced Master Mix (Cat# 4444556, Thermo Fisher Scientific) and the TaqMan Advanced miRNA Assay specific for mouse miR-425-5p (mmu481161_mir; ′3-AAUGACACGAUCA CUCCCGUUGA-5′) (Cat# A25576, Thermo Fisher Scientific).

Statistical analysis

All statistical analyses were performed with Prism 6.0 (Graphpad Software). Data were expressed as the mean ± SD. Differences between different groups were compared by Student t test (i.e., vFC) and two-way ANOVA with multiple comparisons (i.e., wound healing assays), with statistically significant p values indicated as ****$p < 0.0001$, ***$p < 0.001$, **$p < 0.005$, and *$p < 0.05$. All statistical analyzes and representative images presented and observed in at least three independent experiments.

DATA AND CODE AVAILABILITY

scRNA-seq (#GSE242496) and miRNA-seq data (#GSE242497) produced in this study are accessible via the GEO archives maintained by the NCBI. vFC data are deposited into flowrepository.org (#FR-FCM-Z749).

SUPPLEMENTAL INFORMATION

Supplemental information can be found online at https://doi.org/10.1016/j.ymthe.2024.02.019.

ACKNOWLEDGMENTS

Ann-Marie Hageny, Emelie Amburn, Katie Pool, and Michelle Bagood provided expert technical support. The UCSD Tissue Technology Shared Resources (TTSR) core at the Moores Cancer Center provided histology services, the UCSD Nikon Imaging Center confocal microscopy imaging services, and the UCSD Institute for Genomic Medicine for scRNA sequencing that utilized an Illumina NovaSeq 6000 purchased with funding from a National Institutes of Health SIF grant (#S10 OD026929). Schematics and the graphical abstract were created in Biorender.com under license agreement. This work was supported by National Institutes of Health grants (1 R01 GM140137 and 1 R35 GM149245) (B.P.E.) and KL2TR001444 of CTSA funding through the UCSD CTRI (R.D.).

AUTHOR CONTRIBUTIONS

Conceptualization, D.J.P. and B.P.E.; methodology, D.J.P., W.C., K.H., J.K., and J.N.; software D.J.P., W.C., and S.S.; validation, D.J.P. and W.C.; investigation, D.J.P., W.C., S.S., K.H., J.K., J.R., P.M., T.C., R.D., J.N., and B.P.E.; resources, D.J.P. and S.S.; data curation, D.J.P. and S.S.; reagents, J.R. and P.M.; writing – original draft, D.J.P. and B.P.E.; visualization, D.J.P., S.S., and B.P.E.; funding acquisition B.P.E., P.M., and T.C.

DECLARATION OF INTERESTS

The laboratory receives funding support for EV tropism screening research that is unrelated to the studies described herein from Ionis Pharmaceuticals (B.P.E., P.M., and J.P.N.). J.P.N. is the CEO of Cellarcus Biosciences.

REFERENCES

1. Koh, T.J., and DiPietro, L.A. (2011). Inflammation and wound healing: the role of the macrophage. Expert Rev. Mol. Med. *13*, e23. https://doi.org/10.1017/S1462399411001943.
2. Minutti, C.M., Knipper, J.A., Allen, J.E., and Zaiss, D.M.W. (2017). Tissue-specific contribution of macrophages to wound healing. Semin. Cell Dev. Biol. *61*, 3–11. https://doi.org/10.1016/j.semcdb.2016.08.006.
3. Novak, M.L., and Koh, T.J. (2013). Phenotypic transitions of macrophages orchestrate tissue repair. Am. J. Pathol. *183*, 1352–1363. https://doi.org/10.1016/j.ajpath.2013.06.034.
4. Larouche, J., Sheoran, S., Maruyama, K., and Martino, M.M. (2018). Immune Regulation of Skin Wound Healing: Mechanisms and Novel Therapeutic Targets. Adv. Wound Care *7*, 209–231. https://doi.org/10.1089/wound.2017.0761.
5. Hoshino, A., Costa-Silva, B., Shen, T.L., Rodrigues, G., Hashimoto, A., Tesic Mark, M., Molina, H., Kohsaka, S., Di Giannatale, A., Ceder, S., et al. (2015). Tumour exosome integrins determine organotropic metastasis. Nature *527*, 329–335. https://doi.org/10.1038/nature15756.
6. Pluchino, S., and Smith, J.A. (2019). Explicating Exosomes: Reclassifying the Rising Stars of Intercellular Communication. Cell *177*, 225–227. https://doi.org/10.1016/j.cell.2019.03.020.
7. Jeppesen, D.K., Fenix, A.M., Franklin, J.L., Higginbotham, J.N., Zhang, Q., Zimmerman, L.J., Liebler, D.C., Ping, J., Liu, Q., Evans, R., et al. (2019). Reassessment of Exosome Composition. Cell *177*, 428–445.e18. https://doi.org/10.1016/j.cell.2019.02.029.
8. Park, D.J., Duggan, E., Ho, K., Dorschner, R.A., Dobke, M., Nolan, J.P., and Eliceiri, B.P. (2022). Serpin-loaded extracellular vesicles promote tissue repair in a mouse model of impaired wound healing. J. Nanobiotechnology *20*, 474. https://doi.org/10.1186/s12951-022-01656-7.
9. Qin, S., Dorschner, R.A., Masini, I., Lavoie-Gagne, O., Stahl, P.D., Costantini, T.W., Baird, A., and Eliceiri, B.P. (2019). TBC1D3 regulates the payload and biological activity of extracellular vesicles that mediate tissue repair. FASEB J. *33*, 6129–6139. https://doi.org/10.1096/fj.201802388R.
10. Qian, J., Park, D.J., Perrott, S., Patel, P., and Eliceiri, B.P. (2021). Genetic Background and Kinetics Define Wound Bed Extracellular Vesicles in a Mouse Model of Cutaneous Injury. Int. J. Mol. Sci. *22*, 3551. https://doi.org/10.3390/ijms22073551.

11. Silva, A.M., Teixeira, J.H., Almeida, M.I., Gonçalves, R.M., Barbosa, M.A., and Santos, S.G. (2017). Extracellular Vesicles: Immunomodulatory messengers in the context of tissue repair/regeneration. Eur. J. Pharm. Sci. *98*, 86–95. https://doi.org/10.1016/j.ejps.2016.09.017.

12. Veerman, R.E., Teeuwen, L., Czarnewski, P., Güclüler Akpinar, G., Sandberg, A., Cao, X., Pernemalm, M., Orre, L.M., Gabrielsson, S., and Eldh, M. (2021). Molecular evaluation of five different isolation methods for extracellular vesicles reveals different clinical applicability and subcellular origin. J. Extracell. Vesicles *10*, e12128. https://doi.org/10.1002/jev2.12128.

13. Prasai, A., Jay, J.W., Jupiter, D., Wolf, S.E., and El Ayadi, A. (2022). Role of Exosomes in Dermal Wound Healing: A Systematic Review. J. Invest. Dermatol. *142*, 662–678.e8. https://doi.org/10.1016/j.jid.2021.07.167.

14. Sarcinella, A., Femminò, S., and Brizzi, M.F. (2023). Extracellular Vesicles: Emergent and Multiple Sources in Wound Healing Treatment. Int. J. Mol. Sci. *24*, 15709. https://doi.org/10.3390/ijms242115709.

15. Pitt, J.M., Kroemer, G., and Zitvogel, L. (2016). Extracellular vesicles: masters of intercellular communication and potential clinical interventions. J. Clin. Invest. *126*, 1139–1143. https://doi.org/10.1172/JCI87316.

16. Deskins, D.L., Ardestani, S., and Young, P.P. (2012). The polyvinyl alcohol sponge model implantation. J. Vis. Exp. https://doi.org/10.3791/3885.

17. Baird, A., Deng, C., Eliceiri, M.H., Haghi, F., Dang, X., Coimbra, R., Costantini, T.W., Torbett, B.E., and Eliceiri, B.P. (2016). Mice engrafted with human hematopoietic stem cells support a human myeloid cell inflammatory response in vivo. Wound Repair Regen. *24*, 1004–1014. https://doi.org/10.1111/wrr.12471.

18. Crane, M.J., Daley, J.M., van Houtte, O., Brancato, S.K., Henry, W.L., Jr., and Albina, J.E. (2014). The monocyte to macrophage transition in the murine sterile wound. PLoS One *9*, e86660. https://doi.org/10.1371/journal.pone.0086660.

19. Michaels, J., 5th, Churgin, S.S., Blechman, K.M., Greives, M.R., Aarabi, S., Galiano, R.D., and Gurtner, G.C. (2007). db/db mice exhibit severe wound-healing impairments compared with other murine diabetic strains in a silicone-splinted excisional wound model. Wound Repair Regen. *15*, 665–670. https://doi.org/10.1111/j.1524-475X.2007.00273.x.

20. Zhao, G., Hochwalt, P.C., Usui, M.L., Underwood, R.A., Singh, P.K., James, G.A., Stewart, P.S., Fleckman, P., and Olerud, J.E. (2010). Delayed wound healing in diabetic (db/db) mice with Pseudomonas aeruginosa biofilm challenge: a model for the study of chronic wounds. Wound Repair Regen. *18*, 467–477. https://doi.org/10.1111/j.1524-475X.2010.00608.x.

21. Park, S.A., Teixeira, L.B.C., Raghunathan, V.K., Covert, J., Dubielzig, R.R., Isseroff, R.R., Schurr, M., Abbott, N.L., McAnulty, J., and Murphy, C.J. (2014). Full-thickness splinted skin wound healing models in db/db and heterozygous mice: implications for wound healing impairment. Wound Repair Regen. *22*, 368–380. https://doi.org/10.1111/wrr.12172.

22. Dhall, S., Do, D., Garcia, M., Wijesinghe, D.S., Brandon, A., Kim, J., Sanchez, A., Lyubovitsky, J., Gallagher, S., Nothnagel, E.A., et al. (2014). A novel model of chronic wounds: importance of redox imbalance and biofilm-forming bacteria for establishment of chronicity. PLoS One *9*, e109848. https://doi.org/10.1371/journal.pone.0109848.

23. McCann, J.V., Bischoff, S.R., Zhang, Y., Cowley, D.O., Sanchez-Gonzalez, V., Daaboul, G.D., and Dudley, A.C. (2020). Reporter mice for isolating and auditing cell type-specific extracellular vesicles in vivo. Genesis *58*, e23369. https://doi.org/10.1002/dvg.23369.

24. Neckles, V.N., Morton, M.C., Holmberg, J.C., Sokolov, A.M., Nottoli, T., Liu, D., and Feliciano, D.M. (2019). A transgenic inducible GFP extracellular-vesicle reporter (TIGER) mouse illuminates neonatal cortical astrocytes as a source of immunomodulatory extracellular vesicles. Sci. Rep. *9*, 3094. https://doi.org/10.1038/s41598-019-39679-0.

25. Sung, B.H., Ketova, T., Hoshino, D., Zijlstra, A., and Weaver, A.M. (2015). Directional cell movement through tissues is controlled by exosome secretion. Nat. Commun. *6*, 7164. https://doi.org/10.1038/ncomms8164.

26. Fordjour, F.K., Abuelreich, S., Hong, X., Chatterjee, E., Lallai, V., Ng, M., Saftics, A., Deng, F., Carnel-Amar, N., Wakimoto, H., et al. (2023). Exomap1 mouse: a transgenic model for in vivo studies of exosome biology. Preprint at bioRxiv. https://doi.org/10.1101/2023.05.29.542707.

27. Nikoloff, J.M., Saucedo-Espinosa, M.A., Kling, A., and Dittrich, P.S. (2021). Identifying extracellular vesicle populations from single cells. Proc. Natl. Acad. Sci. USA *118*, e2106630118. https://doi.org/10.1073/pnas.2106630118.

28. Giraldez, M.D., Spengler, R.M., Etheridge, A., Godoy, P.M., Barczak, A.J., Srinivasan, S., De Hoff, P.L., Tanriverdi, K., Courtright, A., Lu, S., et al. (2018). Comprehensive multi-center assessment of small RNA-seq methods for quantitative miRNA profiling. Nat. Biotechnol. *36*, 746–757. https://doi.org/10.1038/nbt.4183.

29. Sung, B.H., and Weaver, A.M. (2023). Visualization of Exosome Release and Uptake During Cell Migration Using the Live Imaging Reporter pHluorin_M153R-CD63. Methods Mol. Biol. *2608*, 83–96. https://doi.org/10.1007/978-1-0716-2887-4_6.

30. Blandin, A., Amosse, J., Froger, J., Hilairet, G., Durcin, M., Fizanne, L., Ghesquière, V., Prieur, X., Chaigneau, J., Vergori, L., et al. (2023). Extracellular vesicles are carriers of adiponectin with insulin-sensitizing and anti-inflammatory properties. Cell Rep. *42*, 112866. https://doi.org/10.1016/j.celrep.2023.112866.

31. Daley, J.M., Brancato, S.K., Thomay, A.A., Reichner, J.S., and Albina, J.E. (2010). The phenotype of murine wound macrophages. J. Leukoc. Biol. *87*, 59–67. https://doi.org/10.1189/jlb.0409236.

32. Rani, M., and Schwacha, M.G. (2017). The composition of T-cell subsets are altered in the burn wound early after injury. PLoS One *12*, e0179015. https://doi.org/10.1371/journal.pone.0179015.

33. Engin, A.B. (2017). Adipocyte-Macrophage Cross-Talk in Obesity. Adv. Exp. Med. Biol. *960*, 327–343. https://doi.org/10.1007/978-3-319-48382-5_14.

34. Ellett, F., Pazhakh, V., Pase, L., Benard, E.L., Weerasinghe, H., Azabdaftari, D., Alasmari, S., Andrianopoulos, A., and Lieschke, G.J. (2018). Macrophages protect Talaromyces marneffei conidia from myeloperoxidase-dependent neutrophil fungicidal activity during infection establishment in vivo. PLoS Pathog. *14*, e1007063. https://doi.org/10.1371/journal.ppat.1007063.

35. Wang, S., Song, R., Wang, Z., Jing, Z., Wang, S., and Ma, J. (2018). S100A8/A9 in Inflammation. Front. Immunol. *9*, 1298. https://doi.org/10.3389/fimmu.2018.01298.

36. Wei, J., and Gronert, K. (2019). Eicosanoid and Specialized Proresolving Mediator Regulation of Lymphoid Cells. Trends Biochem. Sci. *44*, 214–225. https://doi.org/10.1016/j.tibs.2018.10.007.

37. Pan, R.Y., Chu, M.T., Wang, C.W., Lee, Y.S., Lemonnier, F., Michels, A.W., Schutte, R., Ostrov, D.A., Chen, C.B., Phillips, E.J., et al. (2019). Identification of drug-specific public TCR driving severe cutaneous adverse reactions. Nat. Commun. *10*, 3569. https://doi.org/10.1038/s41467-019-11396-2.

38. Wang, X., Balaji, S., Steen, E.H., Li, H., Rae, M.M., Blum, A.J., Miao, Q., Butte, M.J., Bollyky, P.L., and Keswani, S.G. (2019). T Lymphocytes Attenuate Dermal Scarring by Regulating Inflammation, Neovascularization, and Extracellular Matrix Remodeling. Adv. Wound Care *8*, 527–537. https://doi.org/10.1089/wound.2019.0981.

39. Amon, L., Lehmann, C.H.K., Baranska, A., Schoen, J., Heger, L., and Dudziak, D. (2019). Transcriptional control of dendritic cell development and functions. Int. Rev. Cell Mol. Biol. *349*, 55–151. https://doi.org/10.1016/bs.ircmb.2019.10.001.

40. Johnson, J., Jaggers, R.M., Gopalkrishna, S., Dahdah, A., Murphy, A.J., Hanssen, N.M.J., and Nagareddy, P.R. (2022). Oxidative Stress in Neutrophils: Implications for Diabetic Cardiovascular Complications. Antioxid. Redox Signal. *36*, 652–666. https://doi.org/10.1089/ars.2021.0116.

41. Hong, W., Yang, B., He, Q., Wang, J., and Weng, Q. (2022). New Insights of CCR7 Signaling in Dendritic Cell Migration and Inflammatory Diseases. Front. Pharmacol. *13*, 841687. https://doi.org/10.3389/fphar.2022.841687.

42. Kim, K., Park, S.E., Park, J.S., and Choi, J.H. (2022). Characteristics of plaque lipid-associated macrophages and their possible roles in the pathogenesis of atherosclerosis. Curr. Opin. Lipidol. *33*, 283–288. https://doi.org/10.1097/MOL.0000000000000842.

43. Deming, Y., Filipello, F., Cignarella, F., Cantoni, C., Hsu, S., Mikesell, R., Li, Z., Del-Aguila, J.L., Dube, U., Farias, F.G., et al. (2019). The MS4A gene cluster is a key modulator of soluble TREM2 and Alzheimer's disease risk. Sci. Transl. Med. *11*, eaau2291. https://doi.org/10.1126/scitranslmed.aau2291.

44. Guo, L., Liu, Y., Chen, Y., Xu, J., and Liu, Y. (2023). Liver macrophages show an immunotolerance phenotype in nonalcoholic fatty liver combined with Porphyromonas gingivalis-lipopolysaccharide infection. Hua Xi Kou Qiang Yi Xue Za Zhi *41*, 385–394. https://doi.org/10.7518/hxkq.2023.2023111.

45. Böing, A.N., van der Pol, E., Grootemaat, A.E., Coumans, F.A.W., Sturk, A., and Nieuwland, R. (2014). Single-step isolation of extracellular vesicles by size-exclusion chromatography. J. Extracell. Vesicles *3*. https://doi.org/10.3402/jev.v3.23430.

46. Sódar, B.W., Kittel, Á., Pálóczi, K., Vukman, K.V., Osteikoetxea, X., Szabó-Taylor, K., Németh, A., Sperlágh, B., Baranyai, T., Giricz, Z., et al. (2016). Low-density lipoprotein mimics blood plasma-derived exosomes and microvesicles during isolation and detection. Sci. Rep. *6*, 24316. https://doi.org/10.1038/srep24316.

47. McFarland-Mancini, M.M., Funk, H.M., Paluch, A.M., Zhou, M., Giridhar, P.V., Mercer, C.A., Kozma, S.C., and Drew, A.F. (2010). Differences in wound healing in mice with deficiency of IL-6 versus IL-6 receptor. J. Immunol. *184*, 7219–7228. https://doi.org/10.4049/jimmunol.0901929.

48. LeBlanc, S., Arabzadeh, A., Benlolo, S., Breton, V., Turbide, C., Beauchemin, N., and Nouvion, A.L. (2011). CEACAM1 deficiency delays important wound healing processes. Wound Repair Regen. *19*, 745–752. https://doi.org/10.1111/j.1524-475X.2011.00742.x.

49. Bourke, C.D., Prendergast, C.T., Sanin, D.E., Oulton, T.E., Hall, R.J., and Mountford, A.P. (2015). Epidermal keratinocytes initiate wound healing and pro-inflammatory immune responses following percutaneous schistosome infection. Int. J. Parasitol. *45*, 215–224. https://doi.org/10.1016/j.ijpara.2014.11.002.

50. Shapira, S., Ben-Amotz, O., Sher, O., Kazanov, D., Mashiah, J., Kraus, S., Gur, E., and Arber, N. (2015). Delayed Wound Healing in Heat Stable Antigen (HSA/CD24)-Deficient Mice. PLoS One *10*, e0139787. https://doi.org/10.1371/journal.pone.0139787.

51. Huang, X., Gu, S., Liu, C., Zhang, L., Zhang, Z., Zhao, Y., Khoong, Y., Li, H., Gao, Y., Liu, Y., et al. (2022). CD39(+) Fibroblasts Enhance Myofibroblast Activation by Promoting IL-11 Secretion in Hypertrophic Scars. J. Invest. Dermatol. *142*, 1065–1076.e19. https://doi.org/10.1016/j.jid.2021.07.181.

52. Noguchi, F., Nakajima, T., Inui, S., Reddy, J.K., and Itami, S. (2014). Alteration of skin wound healing in keratinocyte-specific mediator complex subunit 1 null mice. PLoS One *9*, e102271. https://doi.org/10.1371/journal.pone.0102271.

53. Prabhu, V., Rao, B.S.S., Rao, A.C.K., Prasad, K., and Mahato, K.K. (2022). Photobiomodulation invigorating collagen deposition, proliferating cell nuclear antigen and Ki67 expression during dermal wound repair in mice. Lasers Med. Sci. *37*, 171–180. https://doi.org/10.1007/s10103-020-03202-z.

54. Wang, L., Jiang, J., Zhou, T., Xue, X., and Cao, Y. (2021). Improvement of Cerebral Ischemia-Reperfusion Injury via Regulation of Apoptosis by Exosomes Derived from BDNF-Overexpressing HEK293. Biomed. Res. Int. *2021*, 6613510. https://doi.org/10.1155/2021/6613510.

55. Wang, C., Li, N., Li, Y., Hou, S., Zhang, W., Meng, Z., Wang, S., Jia, Q., Tan, J., Wang, R., and Zhang, R. (2022). Engineering a HEK-293T exosome-based delivery platform for efficient tumor-targeting chemotherapy/internal irradiation combination therapy. J. Nanobiotechnology *20*, 247. https://doi.org/10.1186/s12951-022-01462-1.

56. Zhang, J., Song, H., Dong, Y., Li, G., Li, J., Cai, Q., Yuan, S., Wang, Y., and Song, H. (2023). Surface Engineering of HEK293 Cell-Derived Extracellular Vesicles for Improved Pharmacokinetic Profile and Targeted Delivery of IL-12 for the Treatment of Hepatocellular Carcinoma. Int. J. Nanomedicine *18*, 209–223. https://doi.org/10.2147/IJN.S388916.

57. Qu, Y., Zhang, Q., Cai, X., Li, F., Ma, Z., Xu, M., and Lu, L. (2017). Exosomes derived from miR-181-5p-modified adipose-derived mesenchymal stem cells prevent liver fibrosis via autophagy activation. J. Cell. Mol. Med. *21*, 2491–2502. https://doi.org/10.1111/jcmm.13170.

58. Katakowski, M., Buller, B., Zheng, X., Lu, Y., Rogers, T., Osobamiro, O., Shu, W., Jiang, F., and Chopp, M. (2013). Exosomes from marrow stromal cells expressing miR-146b inhibit glioma growth. Cancer Lett. *335*, 201–204. https://doi.org/10.1016/j.canlet.2013.02.019.

59. Wang, Q., Zhu, G., Cao, X., Dong, J., Song, F., and Niu, Y. (2017). Blocking AGE-RAGE Signaling Improved Functional Disorders of Macrophages in Diabetic Wound. J. Diabetes Res. *2017*, 1428537. https://doi.org/10.1155/2017/1428537.

60. Fernandes, H., Zonnari, A., Abreu, R., Aday, S., Barão, M., Albino, I., Lino, M., Branco, A., Seabra, C., Barata, T., et al. (2022). Extracellular vesicles enriched with an endothelial cell pro-survival microRNA affects skin tissue regeneration. Mol. Ther. Nucleic Acids *28*, 307–327. https://doi.org/10.1016/j.omtn.2022.03.018.

61. de Almeida Oliveira, N.C., Neri, E.A., Silva, C.M., Valadão, I.C., Fonseca-Alaniz, M.H., Zogbi, C., Levy, D., Bydlowski, S.P., and Krieger, J.E. (2022). Multicellular regulation of miR-196a-5p and miR-425-5 from adipose stem cell-derived exosomes and cardiac repair. Clin. Sci. *136*, 1281–1301. https://doi.org/10.1042/CS20220216.

62. Kwon, D.N., Chang, B.S., and Kim, J.H. (2014). MicroRNA dysregulation in liver and pancreas of CMP-Neu5Ac hydroxylase null mice disrupts insulin/PI3K-AKT signaling. Biomed. Res. Int. *2014*, 236385. https://doi.org/10.1155/2014/236385.

63. Yuan, J., Wu, Y., Li, L., and Liu, C. (2020). MicroRNA-425-5p promotes tau phosphorylation and cell apoptosis in Alzheimer's disease by targeting heat shock protein B8. J. Neural Transm. *127*, 339–346. https://doi.org/10.1007/s00702-019-02134-5.

64. Lou, G., Song, X., Yang, F., Wu, S., Wang, J., Chen, Z., and Liu, Y. (2015). Exosomes derived from miR-122-modified adipose tissue-derived MSCs increase chemosensitivity of hepatocellular carcinoma. J. Hematol. Oncol. *8*, 122. https://doi.org/10.1186/s13045-015-0220-7.

65. Lee, H.K., Lee, B.R., Lee, T.J., Lee, C.M., Li, C., O'Connor, P.M., Dong, Z., and Kwon, S.H. (2022). Differential release of extracellular vesicle tRNA from oxidative stressed renal cells and ischemic kidneys. Sci. Rep. *12*, 1646. https://doi.org/10.1038/s41598-022-05648-3.

66. Spiegel, A., Brooks, M.W., Houshyar, S., Reinhardt, F., Ardolino, M., Fessler, E., Chen, M.B., Krall, J.A., DeCock, J., Zervantonakis, I.K., et al. (2016). Neutrophils Suppress Intraluminal NK Cell-Mediated Tumor Cell Clearance and Enhance Extravasation of Disseminated Carcinoma Cells. Cancer Discov. *6*, 630–649. https://doi.org/10.1158/2159-8290.CD-15-1157.

67. Achari, A.E., and Jain, S.K. (2017). Adiponectin, a Therapeutic Target for Obesity, Diabetes, and Endothelial Dysfunction. Int. J. Mol. Sci. *18*, 1321. https://doi.org/10.3390/ijms18061321.

68. Yanai, H., and Yoshida, H. (2019). Beneficial Effects of Adiponectin on Glucose and Lipid Metabolism and Atherosclerotic Progression: Mechanisms and Perspectives. Int. J. Mol. Sci. *20*, 1190. https://doi.org/10.3390/ijms20051190.

69. Lou, R., Chen, J., Zhou, F., Wang, C., Leung, C.H., and Lin, L. (2022). Exosome-cargoed microRNAs: Potential therapeutic molecules for diabetic wound healing. Drug Discov. Today *27*, 103323. https://doi.org/10.1016/j.drudis.2022.07.008.

70. Barman, B., Sung, B.H., Krystofiak, E., Ping, J., Ramirez, M., Millis, B., Allen, R., Prasad, N., Chetyrkin, S., Calcutt, M.W., et al. (2022). VAP-A and its binding partner CERT drive biogenesis of RNA-containing extracellular vesicles at ER membrane contact sites. Dev. Cell *57*, 974–994.e8. https://doi.org/10.1016/j.devcel.2022.03.012.

71. Tang, Y., Yang, L.J., Liu, H., Song, Y.J., Yang, Q.Q., Liu, Y., Qian, S.W., and Tang, Q.Q. (2023). Exosomal miR-27b-3p secreted by visceral adipocytes contributes to endothelial inflammation and atherogenesis. Cell Rep. *42*, 111948. https://doi.org/10.1016/j.celrep.2022.111948.

72. Turk, M.A., Chung, C.Z., Manni, E., Zukowski, S.A., Engineer, A., Badakhshi, Y., Bi, Y., and Heinemann, I.U. (2018). MiRAR-miRNA Activity Reporter for Living Cells. Genes (Basel) *9*, 305. https://doi.org/10.3390/genes9060305.

73. Ying, W., Riopel, M., Bandyopadhyay, G., Dong, Y., Birmingham, A., Seo, J.B., Ofrecio, J.M., Wollam, J., Hernandez-Carretero, A., Fu, W., et al. (2017). Adipose Tissue Macrophage-Derived Exosomal miRNAs Can Modulate In Vivo and In Vitro Insulin Sensitivity. Cell *171*, 372–384.e12. https://doi.org/10.1016/j.cell.2017.08.035.

74. Albanese, M., Chen, Y.F.A., Hüls, C., Gärtner, K., Tagawa, T., Mejias-Perez, E., Keppler, O.T., Göbel, C., Zeidler, R., Shein, M., et al. (2021). MicroRNAs are minor constituents of extracellular vesicles that are rarely delivered to target cells. PLoS Genet. *17*, e1009951. https://doi.org/10.1371/journal.pgen.1009951.

75. Chevillet, J.R., Kang, Q., Ruf, I.K., Briggs, H.A., Vojtech, L.N., Hughes, S.M., Cheng, H.H., Arroyo, J.D., Meredith, E.K., Gallichotte, E.N., et al. (2014). Quantitative and stoichiometric analysis of the microRNA content of exosomes. Proc. Natl. Acad. Sci. USA *111*, 14888–14893. https://doi.org/10.1073/pnas.1408301111.

76. Qi, R., Wang, J., Wang, Q., Qiu, X., Yang, F., Liu, Z., and Huang, J. (2019). MicroRNA-425 controls lipogenesis and lipolysis in adipocytes. Biochim. Biophys. Acta Mol. Cell Biol. Lipids *1864*, 744–755. https://doi.org/10.1016/j.bbalip.2019.02.007.

77. Sung, B.H., von Lersner, A., Guerrero, J., Krystofiak, E.S., Inman, D., Pelletier, R., Zijlstra, A., Ponik, S.M., and Weaver, A.M. (2020). A live cell reporter of exosome secretion and uptake reveals pathfinding behavior of migrating cells. Nat. Commun. *11*, 2092. https://doi.org/10.1038/s41467-020-15747-2.

78. de Jong, O.G., Murphy, D.E., Mäger, I., Willms, E., Garcia-Guerra, A., Gitz-Francois, J.J., Lefferts, J., Gupta, D., Steenbeek, S.C., van Rheenen, J., et al. (2020). A CRISPR-Cas9-

based reporter system for single-cell detection of extracellular vesicle-mediated functional transfer of RNA. Nat. Commun. *11*, 1113. https://doi.org/10.1038/s41467-020-14977-8.

79. Einhauer, A., and Jungbauer, A. (2001). The FLAG peptide, a versatile fusion tag for the purification of recombinant proteins. J. Biochem. Biophys. Methods *49*, 455–465. https://doi.org/10.1016/s0165-022x(01)00213-5.

80. Fajardo, R.J., Karim, L., Calley, V.I., and Bouxsein, M.L. (2014). A review of rodent models of type 2 diabetic skeletal fragility. J. Bone Miner. Res. *29*, 1025–1040. https://doi.org/10.1002/jbmr.2210.

81. Lee, Y., Berglund, E.D., Yu, X., Wang, M.Y., Evans, M.R., Scherer, P.E., Holland, W.L., Charron, M.J., Roth, M.G., and Unger, R.H. (2014). Hyperglycemia in rodent models of type 2 diabetes requires insulin-resistant alpha cells. Proc. Natl. Acad. Sci. USA *111*, 13217–13222. https://doi.org/10.1073/pnas.1409638111.

82. Théry, C., Witwer, K.W., Aikawa, E., Alcaraz, M.J., Anderson, J.D., Andriantsitohaina, R., Antoniou, A., Arab, T., Archer, F., Atkin-Smith, G.K., et al. (2018). Minimal information for studies of extracellular vesicles 2018 (MISEV2018): a position statement of the International Society for Extracellular Vesicles and update of the MISEV2014 guidelines. J. Extracell. Vesicles *7*, 1535750. https://doi.org/10.1080/20013078.2018.1535750.

83. Welsh, J.A., Van Der Pol, E., Arkesteijn, G.J.A., Bremer, M., Brisson, A., Coumans, F., Dignat-George, F., Duggan, E., Ghiran, I., Giebel, B., et al. (2020). MIFlowCyt-EV: a framework for standardized reporting of extracellular vesicle flow cytometry experiments. J. Extracell. Vesicles *9*, 1713526. https://doi.org/10.1080/20013078.2020.1713526.

84. Shpigelman, J., Lao, F.S., Yao, S., Li, C., Saito, T., Sato-Kaneko, F., Nolan, J.P., Shukla, N.M., Pu, M., Messer, K., et al. (2021). Generation and Application of a Reporter Cell Line for the Quantitative Screen of Extracellular Vesicle Release. Front. Pharmacol. *12*, 668609. https://doi.org/10.3389/fphar.2021.668609.

85. Welsh, J.A., Arkesteijn, G.J.A., Bremer, M., Cimorelli, M., Dignat-George, F., Giebel, B., Görgens, A., Hendrix, A., Kuiper, M., Lacroix, R., et al. (2023). A compendium of single extracellular vesicle flow cytometry. J. Extracell. Vesicles *12*, e12299. https://doi.org/10.1002/jev2.12299.

86. Li, M., Wang, T., Tian, H., Wei, G., Zhao, L., and Shi, Y. (2019). Macrophage-derived exosomes accelerate wound healing through their anti-inflammation effects in a diabetic rat model. Artif. Cells Nanomed. Biotechnol. *47*, 3793–3803. https://doi.org/10.1080/21691401.2019.1669617.

87. Hsu, H.H., Wang, A.Y.L., Loh, C.Y.Y., Pai, A.A., and Kao, H.K. (2022). Therapeutic Potential of Exosomes Derived from Diabetic Adipose Stem Cells in Cutaneous Wound Healing of db/db Mice. Pharmaceutics *14*, 1206. https://doi.org/10.3390/pharmaceutics14061206.

88. Love, M.I., Huber, W., and Anders, S. (2014). Moderated estimation of fold change and dispersion for RNA-seq data with DESeq2. Genome Biol. *15*, 550. https://doi.org/10.1186/s13059-014-0550-8.

89. Sahraeian, S.M.E., Mohiyuddin, M., Sebra, R., Tilgner, H., Afshar, P.T., Au, K.F., Bani Asadi, N., Gerstein, M.B., Wong, W.H., Snyder, M.P., et al. (2017). Gaining comprehensive biological insight into the transcriptome by performing a broad-spectrum RNA-seq analysis. Nat. Commun. *8*, 59. https://doi.org/10.1038/s41467-017-00050-4.

90. Ye, Z., Wang, S., Huang, X., Chen, P., Deng, L., Li, S., Lin, S., Wang, Z., and Liu, B. (2022). Plasma Exosomal miRNAs Associated With Metabolism as Early Predictor of Gestational Diabetes Mellitus. Diabetes *71*, 2272–2283. https://doi.org/10.2337/db21-0909.

YMTHE, Volume *32*

Supplemental Information

Defining the activity of pro-reparative extracellular vesicles in wound healing based on miRNA payloads and cell type-specific lineage mapping

Dong Jun Park, Wooil Choi, Sakeef Sayeed, Robert A. Dorschner, Joseph Rainaldi, Kayla Ho, Jenny Kezios, John P. Nolan, Prashant Mali, Todd Costantini, and Brian P. Eliceiri

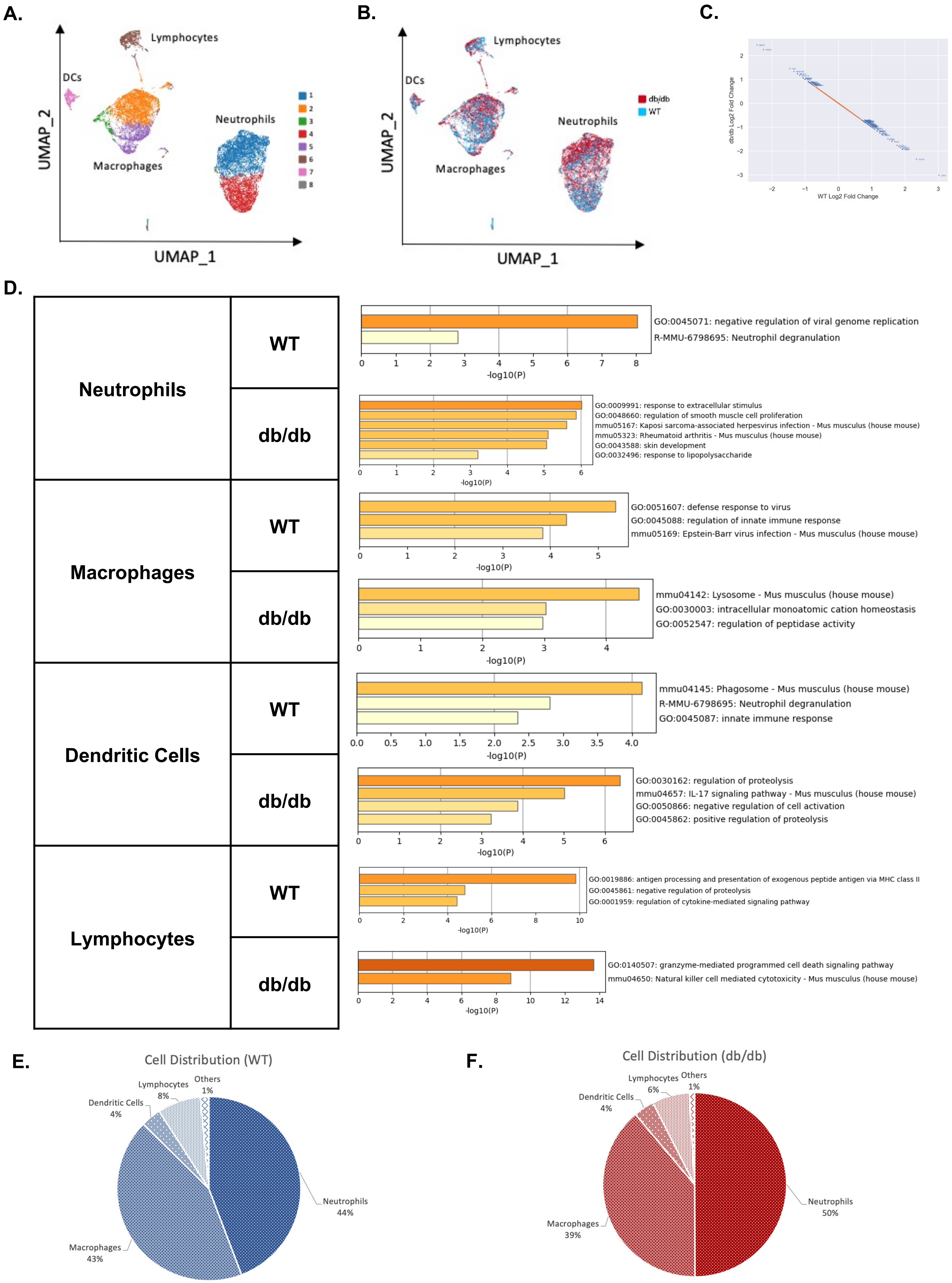

Figure S1. Supporting data for scRNAseq of cells isolated from WT vs db/db donor PVA sponges. (A) Distribution of cell types based on clustering of WT and db/db cells merged. (B) Comparison of cell type clusters based on WT (Blue) vs db/db (red). (C) Statistical analysis of changes in scRNAseq transcript levels in WT vs. db/db mice (n=3 pooled mice from each genotype). (D) GO term analysis comparing changes in gene expression focusing on neutrophils, macrophages, dendritic cells, and lymphocytes. (E and F) Distribution of cell types based on UMAP clustering of canonical genes for each cell type (See Supplementary Table 1 for gene list).

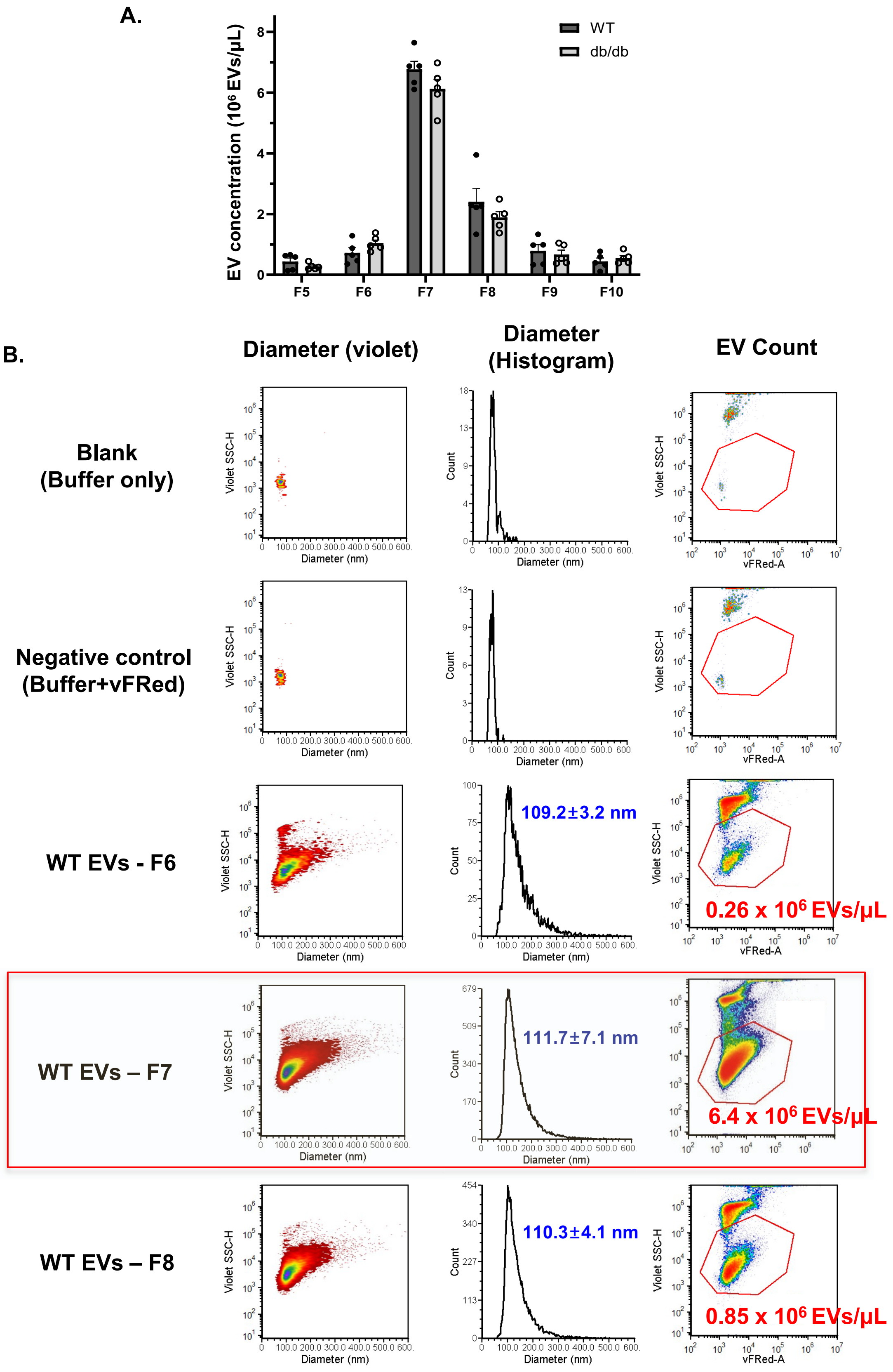

Figure S2. Controls for single vesicle flow cytometry (vFC) based on purification of samples by size exclusion chromatography (SEC). (A) Quantification of EV concentration in each fraction of the SEC from replicate WT vs db/db biological replicates (n=5 independent samples from each genotype) to demonstrate that similar concentrations of EVs recovered in each fraction. (B) Representative controls showing event distribution in a running buffer alone (Blank), Negative Control that has buffer with the fluorescent lipophilic membrane dye vFRed (Cellarcus, San Diego, CA) and no sample, and WT EVs which are samples from a SEC run where the three primary fractions that are recovered in F6-F8 are analyzed with the vFRed dye. In each case, the left column contains plots of size based on calibration with Nanorainbow beads (Cellarcus) vs. Violet SSC-H. The middle column shows diameter vs. count to obtain a size distribution and further supporting the ~110 nm population obtained from PVA sponges. The right column is a distribution of vFred^{+} events vs. Violet SSC-H with concentrated calculated based on events, rate, and volume.

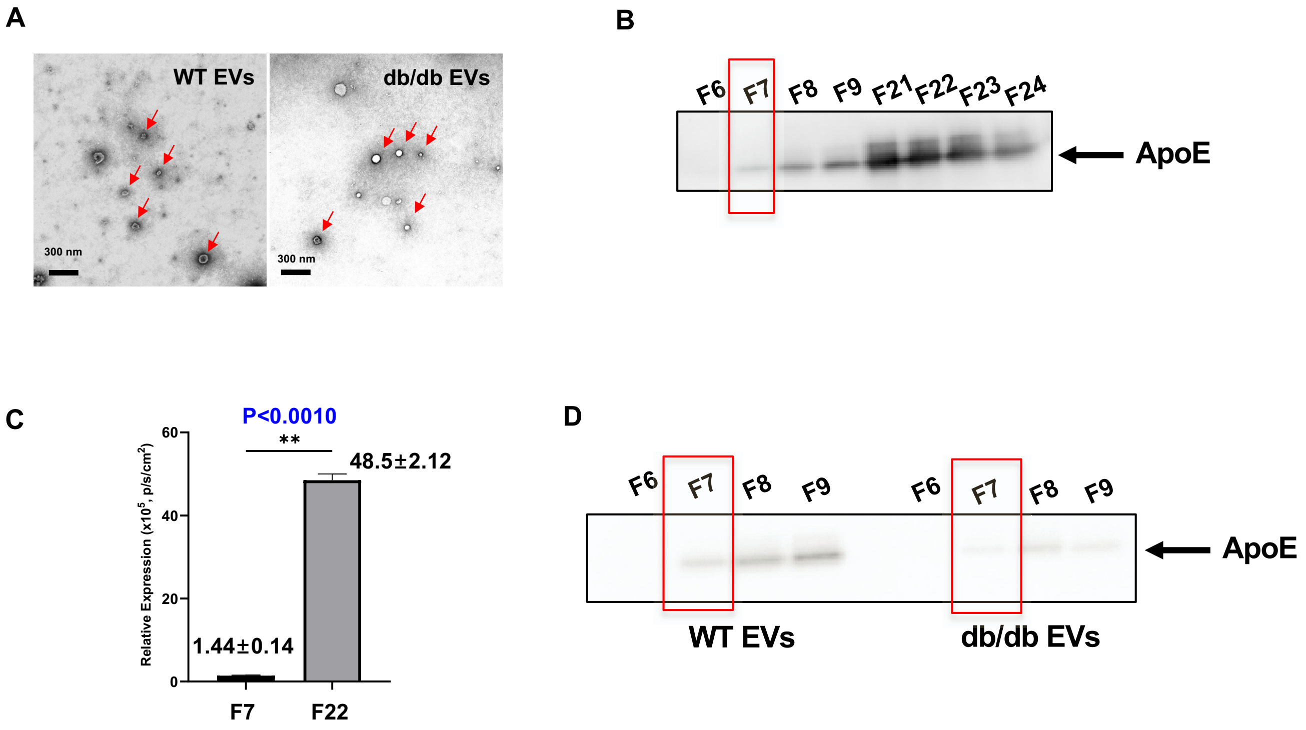

Figure S3. Characterization of EV fractions via electron microscopy (EM) and immunoblotting to monitor levels of ApoE lipoprotein in EVs. (A) Representative EM images of WT vs. db/db EVs. (B) Immunoblots to assess the extent to which size exclusion chromatography separates EVs from lipoproteins. Fractions F7 to F9, are the EV-enriched fractions while fractions and F21 to F23 are the ApoE-enriched fractions. Blots were normalized to have the same volume per fraction since the number of EVs was highest in F7 to F9 based on the data provided in Supplementary Figure 2. (C) Quantification of ApoE expression of a peak fraction from the EV-enriched range (F7) vs. the ApoE-enriched range (F22), showing there was 33.6 times lower ApoE in the EV fractions compared to F22 where ApoE containing lipoproteins would be expected to be detected in SEC. (D) For Panel D, the amount of protein added to each lane was normalized to have the same number of EVs for each fraction (i.e., WT vs db/db) based on the data provided in Supplementary Figure 2.

A.

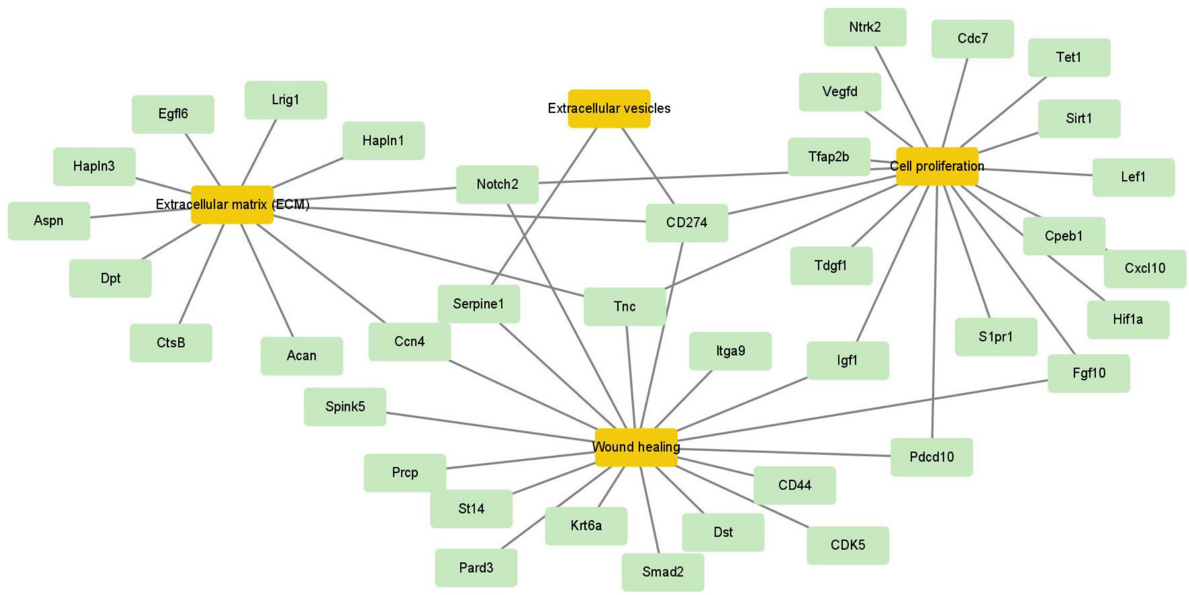

B.

GO ID	Transcript ID	Gene ID	Symbol	Gene_Annoation	GO Function	Target scan Score
GO:0016477	ENSMUST00000154617	ENSMUSG00000020516	Rps6kb1	ribosomal protein S6 kinase, polypeptide 1 [Source:MGI Symbol;Acc:MGI:1270849]	cell migration	99
GO:0010634	ENSMUST00000021530	ENSMUSG00000021109	Hif1a	hypoxia inducible factor 1, alpha subunit [Source:MGI Symbol;Acc:MGI:106918]	cell migration	98
GO:0030335	ENSMUST00000130310	ENSMUSG00000025586	Cpeb1	cytoplasmic polyadenylation element binding protein 1 [Source:MGI Symbol;Acc:MGI:108442]	cell migration	98
GO:0016477	ENSMUST00000030814	ENSMUSG00000028969	CDK5	cyclin-dependent kinase 5 [Source:MGI Symbol;Acc:MGI:101765]	cell migration	98
GO:0005518	ENSMUST00000021820	ENSMUSG00000021388	Aspn	asporin [Source:MGI Symbol;Acc:MGI:1913945]	Collagen binding	98
GO:0008284	ENSMUST00000146028	ENSMUSG00000020063	Sirt1	sirtuin 1 [Source:MGI Symbol;Acc:MGI:2135607]	Cell proliferation	97
GO:0044319	ENSMUST00000160272	ENSMUSG00000025812	Pard3	par-3 family cell polarity regulator [Source:MGI Symbol;Acc:MGI:2135608]	Wound healing	96
GO:0030335	ENSMUST00000029611	ENSMUSG00000027985	Lef1	lymphoid enhancer binding factor 1 [Source:MGI Symbol;Acc:MGI:96770]	cell migration	96
GO:0098633	ENSMUST00000173598	ENSMUSG00000063765	Chadl	chondroadherin-like [Source:MGI Symbol;Acc:MGI:3036284]	Collagen binding	95
GO:0016477	ENSMUST00000005218	ENSMUSG00000005087	CD44	CD44 antigen [Source:MGI Symbol;Acc:MGI:88338]	cell migration	94
GO:0031012	ENSMUST00000101126	ENSMUSG00000030029	Lrig1	leucine-rich repeats and immunoglobulin-like domains 1 [Source:MGI Symbol;Acc:MGI:107935]	Extracellular matrix (ECM)	93
GO:0030335	ENSMUST00000161137	ENSMUSG00000027835	Pdcd10	programmed cell death 10 [Source:MGI Symbol;Acc:MGI:1928396]	cell migration	92
GO:0008284	ENSMUST00000038816	ENSMUSG00000034855	Cxcl10	chemokine (C-X-C motif) ligand 10 [Source:MGI Symbol;Acc:MGI:1352450]	Cell proliferation	92
GO:0031012	ENSMUST00000005255	ENSMUSG00000005124	Ccn4	cellular communication network factor 4 [Source:MGI Symbol;Acc:MGI:1197008]	Extracellular matrix (ECM)	92
GO:0008284	ENSMUST00000079812	ENSMUSG00000027878	Notch2	notch 2 [Source:MGI Symbol;Acc:MGI:97364]	Cell proliferation	90
GO:0010634	ENSMUST00000022246	ENSMUSG00000021732	Fgf10	fibroblast growth factor 10 [Source:MGI Symbol;Acc:MGI:1099809]	cell migration	90
GO:0030335	ENSMUST00000038816	ENSMUSG00000034855	Cxcl10	chemokine (C-X-C motif) ligand 10 [Source:MGI Symbol;Acc:MGI:1352450]	cell migration	86
GO:0008284	ENSMUST00000129938	ENSMUSG00000029283	Cdc7	cell division cycle 7 (S. cerevisiae) [Source:MGI Symbol;Acc:MGI:1309511]	Cell proliferation	84
GO:0030335	ENSMUST00000205460	ENSMUSG00000030789	Itgax	integrin alpha X [Source:MGI Symbol;Acc:MGI:96609]	cell migration	83
GO:0070062	ENSMUST00000041388	ENSMUSG00000037411	Serpine1	serine (or cysteine) peptidase inhibitor, clade E, member 1 [Source:MGI Symbol;Acc:MGI:97608]	Extracellular vesicles	82
GO:0030335	ENSMUST00000197460	ENSMUSG00000032494	Tdgf1	teratocarcinoma-derived growth factor 1 [Source:MGI Symbol;Acc:MGI:98658]	cell migration	81
GO:0016477	ENSMUST00000146179	ENSMUSG00000020282	Rhbdf1	rhomboid 5 homolog 1 [Source:MGI Symbol;Acc:MGI:104328]	cell migration	78
GO:0008284	ENSMUST00000033751	ENSMUSG00000031380	Vegfd	vascular endothelial growth factor D [Source:MGI Symbol;Acc:MGI:108037]	Cell proliferation	76
GO:0008284	ENSMUST00000030056	ENSMUSG00000028364	Tnc	tenascin C [Source:MGI Symbol;Acc:MGI:101922]	Cell proliferation	76
GO:0030335	ENSMUST00000095360	ENSMUSG00000020053	Igf1	insulin-like growth factor 1 [Source:MGI Symbol;Acc:MGI:96432]	cell migration	73
GO:0008284	ENSMUST00000224259	ENSMUSG00000055254	Ntrk2	neurotrophic tyrosine kinase, receptor, type 2 [Source:MGI Symbol;Acc:MGI:97384]	Cell proliferation	71
GO:0005581	ENSMUST00000036737	ENSMUSG00000038591	Colec10	collectin sub-family member 10 [Source:MGI Symbol;Acc:MGI:3606482]	Collagen binding	71
GO:0008284	ENSMUST00000050826	ENSMUSG00000047146	Tet1	tet methylcytosine dioxygenase 1 [Source:MGI Symbol;Acc:MGI:1098693]	Cell proliferation	70
GO:0004867	ENSMUST00000069245	ENSMUSG00000055561	Spink5	serine peptidase inhibitor, Kazal type 5 [Source:MGI Symbol;Acc:MGI:1919682]	serine-type inhibitor activity	70
GO:0008284	ENSMUST00000027059	ENSMUSG00000025927	Tfap2b	transcription factor AP-2 beta [Source:MGI Symbol;Acc:MGI:104672]	Cell proliferation	69
GO:0042060	ENSMUST00000168423	ENSMUSG00000024563	Smad2	SMAD family member 2 [Source:MGI Symbol;Acc:MGI:108051]	Wound healing	69
GO:0030335	ENSMUST00000016640	ENSMUSG00000016496	CD274	CD274 antigen [Source:MGI Symbol;Acc:MGI:1926446]	cell migration	67
GO:0016477	ENSMUST00000055676	ENSMUSG00000045092	S1pr1	sphingosine-1-phosphate receptor 1 [Source:MGI Symbol;Acc:MGI:1096355]	cell migration	66
GO:0008236	ENSMUST00000076052	ENSMUSG00000061119	Prcp	prolylcarboxypeptidase (angiotensinase C) [Source:MGI Symbol;Acc:MGI:1919711]	serine-type peptidase	66
GO:0004252	ENSMUST00000234712	ENSMUSG00000033825	Tpsb2	tryptase beta 2 [Source:MGI Symbol;Acc:MGI:96942]	serine-type peptidase	63
GO:0016477	ENSMUST00000021028	ENSMUSG00000020689	Itgb3	integrin beta 3 [Source:MGI Symbol;Acc:MGI:96612]	cell migration	55
GO:0005581	ENSMUST00000170091	ENSMUSG00000025044	Msr1	macrophage scavenger receptor 1 [Source:MGI Symbol;Acc:MGI:98257]	Collagen binding	54
GO:0016477	ENSMUST00000123557	ENSMUSG00000031995	St14	suppression of tumorigenicity 14 (colon carcinoma) [Source:MGI Symbol;Acc:MGI:1338881]	cell migration	50
GO:0042060	ENSMUST00000183302	ENSMUSG00000026131	Dst	dystonin [Source:MGI Symbol;Acc:MGI:104627]	Wound healing	50

Figure S4. GO term analysis of candidate miR-425-5p targets.

(A) Interaction map highlighting potential relevance of miR-425-5p on cell proliferation, cell migration, and wound healing. (B) Table listing GO term-based target scan scores.

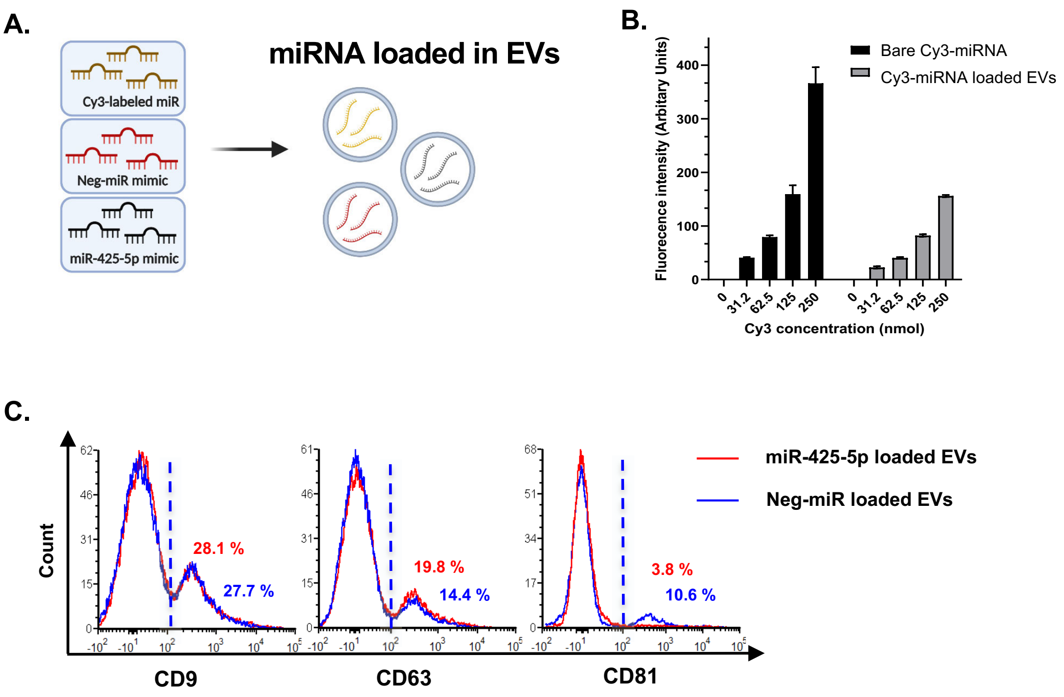

Figure S5. Optimization and encapsulation efficiency of miR-425-5p in EVs.

(A) Schematic of miRNA loading of EVs isolated from WT PVA sponge implants using Exofect per manufacturer's recommendation (Systems Biosciences, SBI). Neg-miR (cel-miR-67), miR-425-5p, and Cy3-labeled miRNA were compared, the Cy3-miRNA was provided as an internal control from SBI for titrating EV loading with respect to the amount of miRNA added. (B) Optimization of conditions for the encapsulation Cy-labeled miRNAs EVs using miRNAs in a range of 31-250 nmoles in a volume of 100 µL using a fluorescence plate reader. (C) Measurement of encapsulation efficiency based on Cy3-miRNA levels in EVs. (E) vFC using antibodies directed to the tetraspanins showing that the levels of CD9, CD63 and CD81 were unchanged between loading with miR-425-5p vs. Neg-miR loaded EVs before use for in vivo studies. Red line: miR-425-5p loaded EVs. Blue line: Neg-miR loaded EVs.

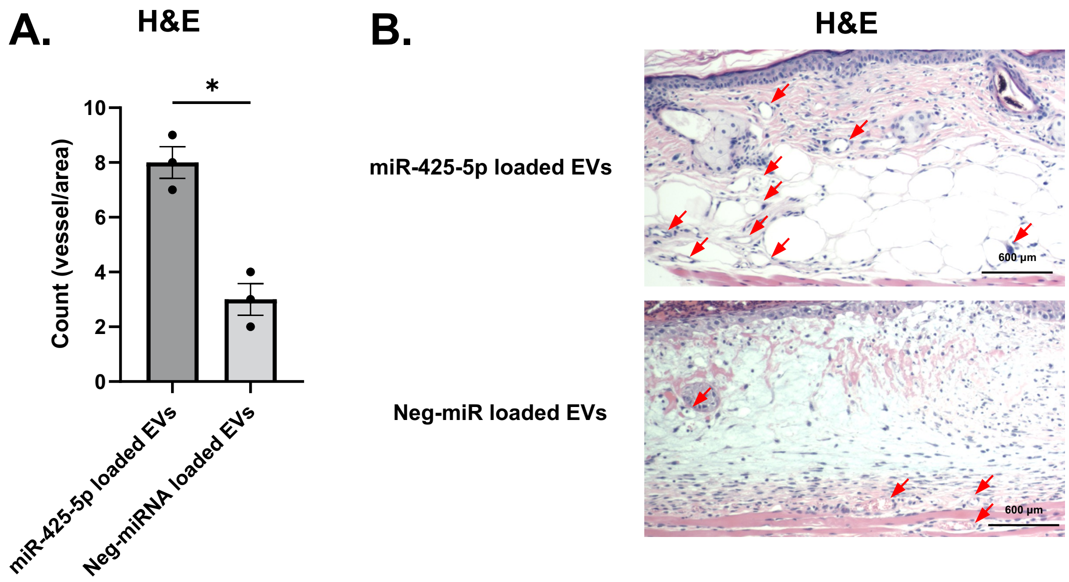

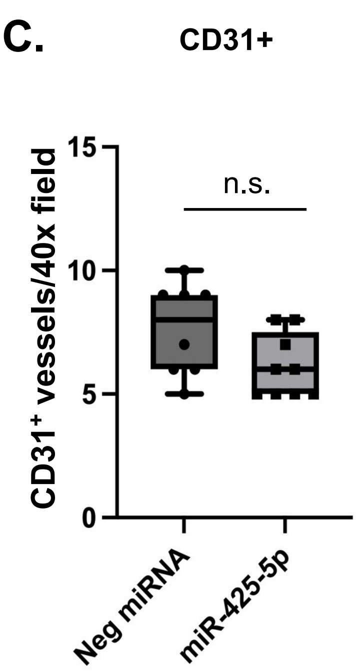

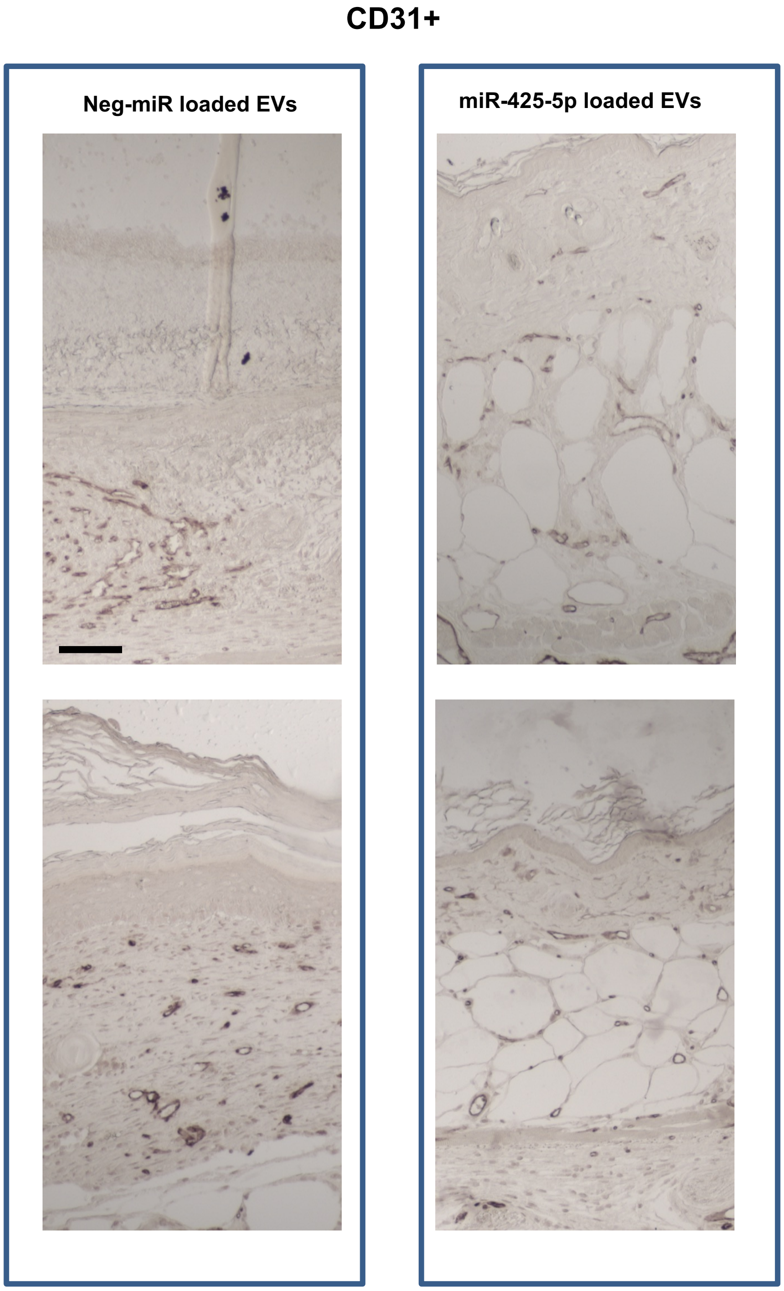

Figure S6. Analysis of blood vessel density in wound bed following treatment with miR-425-5p-loaded EVs vs. Neg control miRNA. (A) Enumeration of blood vessels based on hematoxylin/eosin-stained sections of wound bed showing a significant increase in vessel density ($P < 0.05$). (B) Analysis of CD31-stained tissue sections of bed (sum of 4 different 40x fields from 2 different mice for each group). (C) Representative images from each group showing similar levels of $CD31^{+}$ positive. Note in Neg miRNA EV treatment had $CD31^{+}$ vessels primarily in the dermis, while miR-425-5p EV treatment led to vessels widely distributed in both dermis and a more prominent adipose layer.

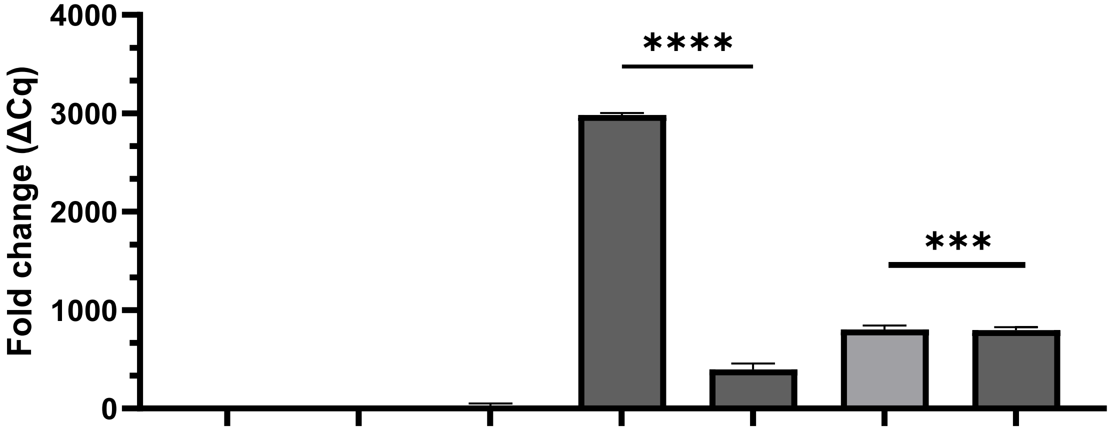

	1	2	3	4	5	6	7
EVs	-	+	+	-	-	+	+
miR-425-5p	-	-	-	+	+	+	+
Benzonase	+	-	+	-	+	-	+

Figure S7. Demonstration that EVs loaded with miR-425-5p were resistant to treatment with the nuclease benzonase. Incubation of nanoparticles such as EVs with benzonase can be used to determine whether nucleic acid payloads are on the surface or contained within a particle. Here we show that while treatment with bezonase degraded free miR-425-5p (Groups 4 and 5), treatment of EVs loaded with miR-425-5p did not show a significant reduction (Groups 6 and 7). We treated miR-loaded EVs, prepared using Exofect as described in the materials and methods (2.8 x 10^8 EVs), with benzonase nuclease (10 Unit/µL, 3 min). RNA was extracted with a mirVana™ miRNA Isolation Kit (Cat#AM1560, Thermo Fisher, Carlsbad, CA), and cDNA synthesized with the TaqMan™ Advanced miRNA cDNA Synthesis Kit (Cat# A28007, Thermo Fisher). qRT-PCR was performed on CFX96 (Bio-Rad) using the TaqMan™ Fast Advanced Master Mix for qPCR (Cat# 4444556, Thermo Fisher) and TaqMan™ Advanced miRNA Assay (mmu481161_mir) (Cat# A25576, Thermo Fisher). PCR reaction followed manufacturer's recommendations using the FAM fluorophore (Cycles: 95°C for 20 sec followed by 40 cycles of 95°C for 3 sec and 60°C 30 sec).

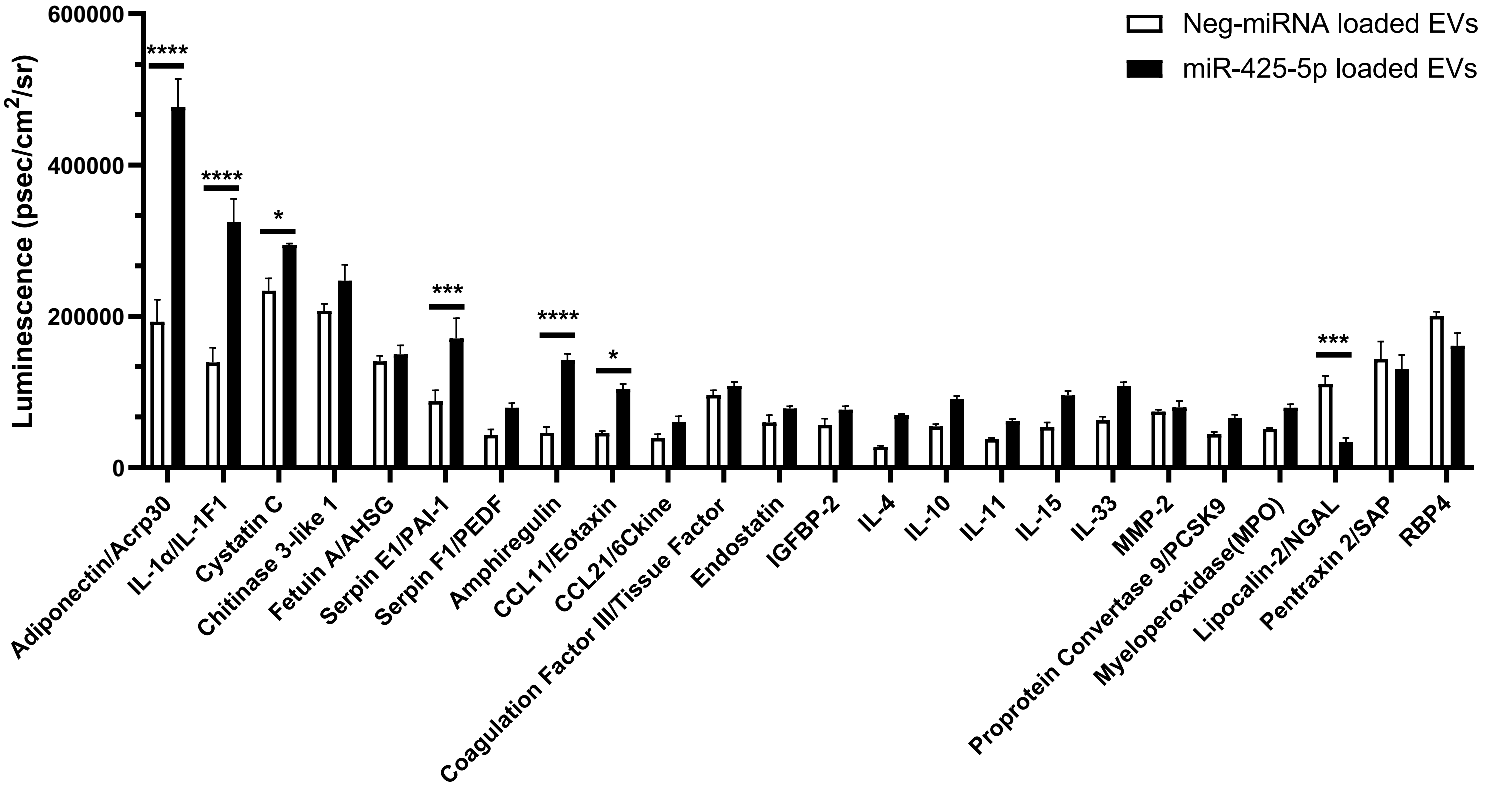

Figure S8. Bar graph of cytokine changes identified after treatment of miR-425-5p-loaded EVs vs. Neg-miRNA loaded EVs to the diabetic wounds. Adiponectin, IL-1α, Cystatin C, Serpin E1, Amphiregulin, and CCL11 were significantly increased when miR-425-5p-loaded EVs were treated to diabetic wounds. Conversely, Lipocalin-2 was significantly reduced when miR-425-5p-loaded EVs were treated to diabetic wounds. (p-value: ****<0.0001, ***<0.001, *<0.05 by Student t-test).

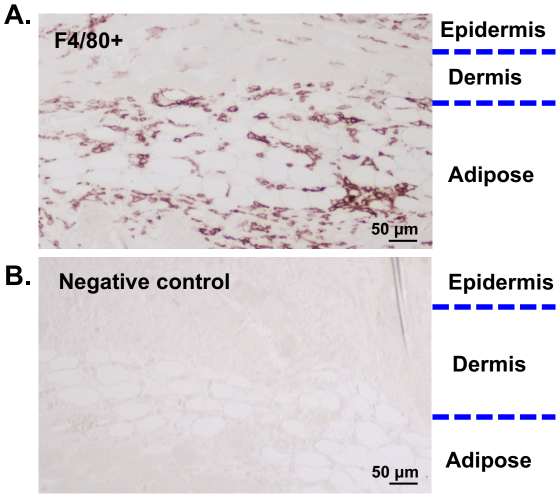

Figure S9. Expression of F4/80 positive macrophages generated in naïve adipose tissue.
(A) Localization of F4/80-positive cells in full thickness sections of mouse skin. (B) Negative control for the above using a secondary and detection reagent without the primary antibody.

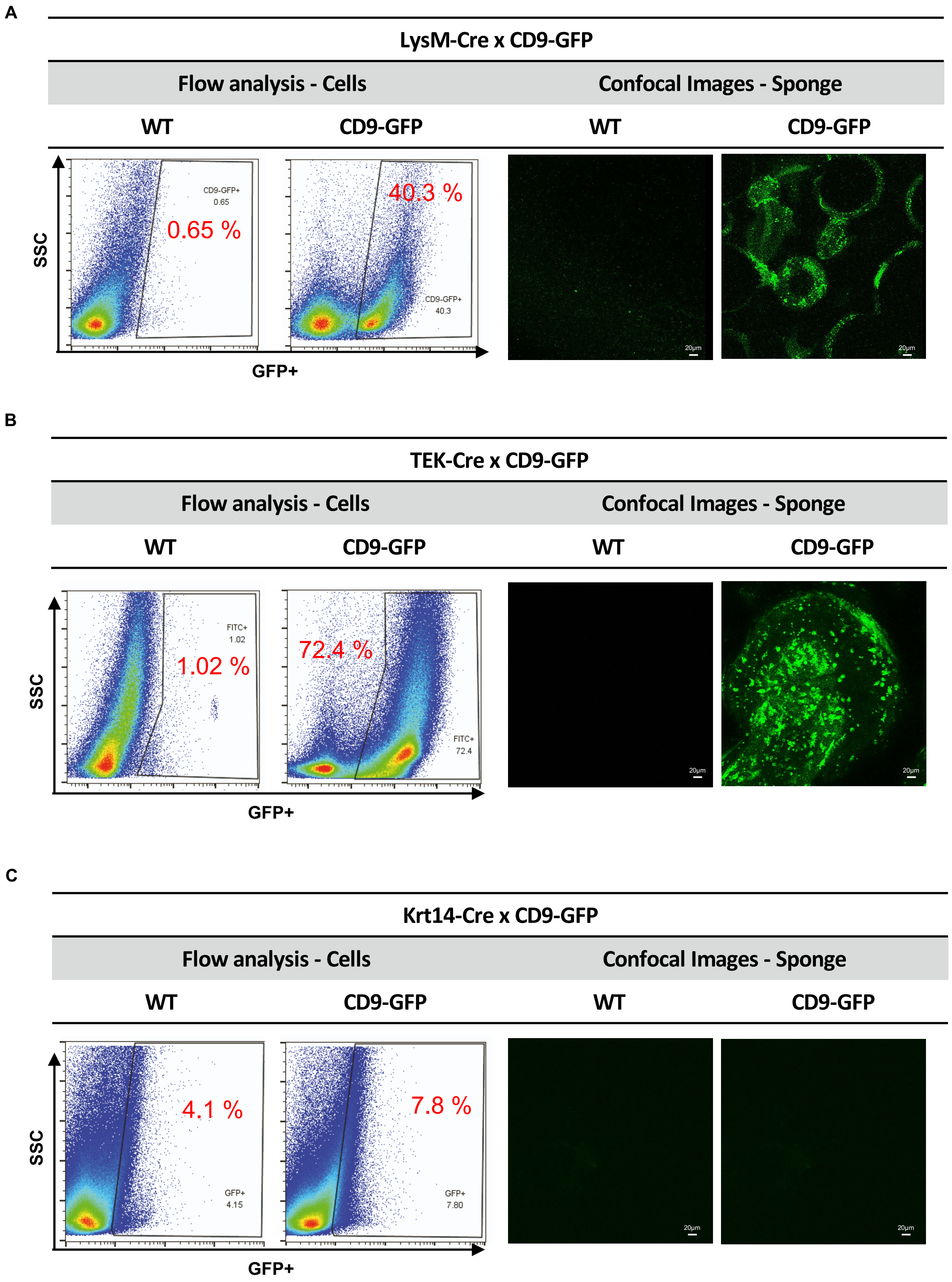

Figure S10. Analysis of GFP$^+$ cells by flow cytometry and confocal microscopy to support EV analyses using cell-type specific promoter models. (A) Analysis of cells PVA sponge implants harvested from macrophage LysM-Cre x CD9-GFP vs. WT control mice. (B) Cells from endothelial TEK-Cre x CD9-GFP mice. (C) Cells from keratinocyte Krt14-Cre x CD9-GFP mice.

Table S1. The list of genes associated with each cell type identified by scRNAseq.

Cell Type	***Gene***	***References***
Macrophages	TREM2	Macrophage marker, inflammation and, atherosclerosis (PMID 35942822)
	IGF1	Adipocyte Macrophage crosstalk in obesity (PMID 28585206)
	H2-DMB1	Macrophages in a fatty liver disease (PMID 37474470)
	MS4A4C	Member of MS4A tetraspan family that modulates TREM2 (31413141)
Neutrophils	S100A8	S100A8/A9 in inflammation (PMID 29942307)
	S100A9	Oxidative stress in neutrophils in diabetes (PMID 34148367)
	CSF3	Neutrophil-mediated inflammation responses in infection (PMID 29883484)
Dendritic cells	CCR7	Regulation of DCs in migration and inflammation (PMID 35281921)
	ZBTB46	Transcriptional control of DCs (PMID 31759434)
Lymphocytes	CD3D	T lymphocytes regulating scarring in wound healing (PMID 31637099)
	CD4E	T cells in adverse cutaneous reactions (PMID 31395875)
	CD3G	T cell subsets in burn wounds (PMID 28575063)
	LTB	Leukotrienes in regulation of lymphoid cells (PMID 30477730)

Table S2. The list of up- and down- regulated miRNAs with target pathway and genes.

	miRNA	*Candidate Target pathway (miRPathDB v.2.0)*	*Gene Targets Observed in Proteome Profiler (Fig 6J) (TargetScanMouse V.7.1)*	*References*
Down-regulation	mmu-miR-425-5p	Dysregulated in Insulin/PI3K-AKT Signaling	SERPIN E1(ENSMUST00000041388.11)	Endothelial pro-survival, Angiogenesis, wound repair (PMID 35474734) Adipose stem cell exosomes, regulate macrophage polarization, cardiac repair (PMID 35894060)
	mmu-miR-186-5p	Hippo signaling pathway	IL-15 (ENSMUST00000034148.7)	Wound healing, infection, immunoregulation (PMID 33017084) Wound healing, macrophage migration, proliferation (PMID 37565786)
	mmu-miR-142a-5p	Focal adhesion	-	Promotes dendritic cell maturation (PMID 35668915) Control profibrogenic macrophage (PMID 26436920)
	mmu-miR-361-3p	Proteoglycans in cancer	-	Wound healing, Migration, proliferation (PMID 34475974) Wound healing, proliferation, inflammatory cell recruitment (PMID 30487794)
	mmu-miR-3068-3p	Metabolic pathways	-	Ischemic stroke, neuronal injury, excitotoxicity (PMID 31792968)
Up-regulation	mmu-let-7e-5p	Inflammatory response pathway	IL-10 (ENSMUST00000016673.6)	Inflammation, Oxidative stress, insulin signaling (PMID 33445738) Inflammation, endotoxemia responses (PMID 33993048)
	mmu-miR-409-5p	Bacterial invasion of epithelial cells	-	Epithelial-mesenchymal transition, Tumorigenesis (PMID 25065597) Regulates Wnt/beta catenin signaling (PMID 31165485)
	mmu-miR-181b-5p	MAPK signaling pathway	SERPIN E1(ENSMUST00000041388.11)	Wound healing, proliferation, inhibit apoptosis (PMID 30783419) Diabetic wounds, senescence, angiogenesis, wound healing (PMID 35528840)
	mmu-miR-541-5p	ErbB signaling pathway	-	Proliferation, hyperglycemia-induced migration, angiogenesis dysfunction (PMID 36291965) Cell differentiation, proliferation, innate immune response, anticoagulation mechanisms (PMID 37996554)

Table S3. The list of animals and cell line.

NAME	SOURCE	CATALOG #
C57B6/J	Jackson lab	JAX000664
$Lepr^{db/db}$	Jackson lab	JAX000697
B6.129P2-$Lyz2^{tm1(cre)Ifo}$/J	Jackson lab	JAX004781
B6.Cg-Tg(Tek-cre)12Flv/J	Jackson lab	JAX004128
B6N.Cg-Tg(KRT14-cre)1Amc/J	Jackson lab	JAX018964
B6;129S1-Gt(ROSA)$26Sort^{m1(CAG-CD9/GFP)Dmfel}$/J	Jackson lab	JAX033361
HEK293T	ATCC	CRL-1573
Mouse embryonic fibroblasts (MEFs)	ATCC	SCRC-1008

Table S4. The list of critical commercial assays kit.

NAME	SOURCE	CATALOG #
Chromium Next GEM Single Cell 3' Kit	10X Genomics	Cat# 1000269 (ver 3.1)
vFC analysis assay kit	Cellarcus Biosciences	Cat# CBS4HP-1PE
ExoQuick kit-TC	System Biosciences	Cat# EQULTRA-20A-1
MACSPlex Exosome Kit, mouse	Miltenyi Biotec	Cat# 130-122-211
BCA assay kit	Thermo Fisher	Cat# 23225
Exo-Fect™ Transfection kit	System Biosciences	Cat# EXFT20A-1
Mouse XL Cytokine Array kit	R & D Systems	Cat# ARY028

Table S5. The list of oligonucleotides for miRNA assay and Taqman qPCR.

NAME	SOURCE	CATALOG #
miR-425-5p mimic	Horizon	Cat# C-310988-01-0050
Negative-miR mimic (cel-miR-67; from C. elegans)	Horizon	Cat# CN-001000-01-50
Cy3-labeled miR (from SBI Kit)	System Biosciences	Cat# EXFT20A-1
mirVana™ miRNA Isolation Kit, with phenol	Thermo Fisher	Cat# AM1560
TaqMan™ Advanced miRNA cDNA Synthesis Kit	Thermo Fisher	Cat# A28007
TaqMan™ Fast Advanced Master Mix for qPCR	Thermo Fisher	Cat# 4444556
TaqMan™ Advanced miRNA Assay (mmu481161_mir)	Thermo Fisher	Cat# A25576

Table S6. Antibodies for immunoblotting and vFC.

Antibodies		
**NAME**	**SOURCE**	**CATALOG #**
CD9-PE (mouse)	Miltenyi Biotec	Cat# 130-123-052
CD81-PE (mouse)	Miltenyi Biotec	Cat# 130-102-632
CD63-PE (mouse)	Miltenyi Biotec	Cat# 130-123-289
MHC class I-PE (mouse)	Biolegend	Cat# 114607
CD29-PE (mouse)	Miltenyi Biotec	Cat# 130-119-165
CD274(PD-L1)-PE (mouse)	Biolegend	Cat# 124307
CD39-PE (mouse)	Miltenyi Biotec	Cat# 130-114-357
CD44-PE (mouse)	Miltenyi Biotec	Cat# 130-118-694
CD45-PE (mouse)	Miltenyi Biotec	Cat# 130-110-797
CD11b-PE (mouse)	Miltenyi Biotec	Cat# 130-113-806
CD54(ICAM-1)-PE (mouse)	Miltenyi Biotec	Cat# 130-104-214
CD49e-PE (mouse)	Miltenyi Biotec	Cat# 130-122-072
CD24-PE (mouse)	Miltenyi Biotec	Cat# 130-110-826
CD126(IL-6R)-PE (mouse)	Biolegend	Cat# 115806
CD66a-PE (mouse)	Miltenyi Biotec	Cat# 130-125-525
CD326(Ep-CAM)-PE (mouse)	Biolegend	Cat# 118205
Isotype-PE	Miltenyi Biotec	Cat# 130-113-450
Anti-Alix (E6P9B) mAb (mouse)	Cell signaling	Cat# 92880
Anti-CD9 (mouse)	Thermo Fisher	Cat# PA5-85955
Anti-CD81 (D5O2Q) (mouse)	Cell signaling	Cat# 10037
Anti-CD63 (mouse)	Thermo Fisher	Cat# PA5-92370
Anti-FLAG antibody	Sigma Aldrich	Cat# F-1804
Anti-mouse IgG, HRP-linked antibody	Cell signaling	Cat# 7076
Anti-rabbit IgG, HRP-linked antibody	Cell signaling	Cat# 7074
Anti-Rabbit HRP Polymer	Cell IDX	Cat# 2RH-100
Ki-67 Rabbit monoclonal Antibody (1:50)	GeneTex	Cat# 16667
Vimentin (D21H3) mAb	Cell signaling	Cat# 5741
Goat anti-Rabbit IgG-Alexa Fluor 546	Thermo Fisher	Cat# A11010
Goat anti-Mouse IgG-Alexa Fluor 546	Thermo Fisher	Cat# A11030
Anti-FLAG-vTag^{TM} Antigody	Cellarcus Biosciences	Cat# CBS18-PE-100T
Goat anti-Rabbit IgG (H+L) Cross-Adsorbed Secondary Antibody, Alexa Fluor™ 488	Thermo Fisher	Cat# A-11008
ApoE	Cell signaling	Cat#49285

Table S7. The list of chemicals and sources.

Antibodies		
**NAME**	**SOURCE**	**CATALOG #**
DMEM-high glucose medium	Thermo Fisher	Cat# 12430054
Fetal Bovine Serum (FBS)	Sigma Aldrich	Cat# F0926
Antibiotic-Antimycotic (100X)	Thermo Fisher	Cat# 15240062
Lipofectamine 3000	Thermo Fisher	Cat# L3000150
RIPA Lysis and Extraction Buffer	Thermo Fisher	Cat# 89901
Tissue-Tek® O.C.T. Compound	Sakura	Cat# 4583
Trizol	Thermo Fisher	Cat# 15596018
RNA 6000 Nano	Agilent	Cat# 5067-1511
NEB Next® Magnesium RNA Fragmentation Module	NEB	Cat# e6150
Super Script™ II Reverse Transcriptase	Invitrogen	Cat# 1896649
DNA polymerase I (E.coli)	NEB	Cat# m0209
RNase H	NEB	Cat# m0297
dUTP Solution	Thermo Fisher	Cat# R0133
UDG enzyme	NEB	Cat# m0280
PVA sponge (7mm x 3mm Disk)	PVA Unlimited Inc.	Cat# SQ5000<br>PVA; PO#PR00440056
Oasis Nylon Suture	Thermo Fisher	Cat# MV-663-V-19mm
Betadine (10% povidone-iodine topical solution)	Thermo Fisher	Cat# NDC 67618-150-01
4 mm punch	Thermo Fisher	Cat# P450
Silicone ring (Thickness: 0.5 mm, outside diameter: 12 mm, inside diameter: 6 mm)	MCS	Cat# 33350174
Surgical glue	Thermo Fisher	Cat# 1469SB
Tegaderm	3M	Cat# 1622w
qEV1-column	IZON	Cat# ICI-70
MACSQuant Running Buffer	Miltenyi Biotec	Cat# 130-092-747
10% BSA stock solution	Miltenyi Biotec	Cat# 130-091-376
vCalTM nano rainbow beads	Cellarcus Biosciences	Cat# CBS6M
vFRed dye	Cellarcus Biosciences	Cat# CBS4A
vFC staining buffer	Cellarcus Biosciences	Cat# CBS0
Liposome control	Cellarcus Biosciences	Cat# CBS1
mouse platelet derived EVs	Cellarcus Biosciences	Cat# CBS2
96-well v bottom plate	Sarstedt	Cat# NC0068972
NuPAGE™ LDS Sample Buffer	Thermo Fisher	Cat# NP0008
1 mm pre-cast 12% Bis-Tris Mini Gel	Thermo Fisher	Cat# NP0342BOX
PVDF membrane	Thermo Fisher	Cat# LC2005
Memcode stain	Thermo Fisher	Cat# 24585
Pierce™ ECL Reagent	Thermo Fisher	Cat# 32209
1X Tris-buffered saline	CST	Cat# 9997
EM grids	Ted Pella, Inc.	Cat# 01754-F
Benzonase	Sigma Aldrich	Cat# E1014-5kU

Table S8. The list of analysis software and equipment.

NAME	SOURCE	CATALOG #
HiSeq sequencers	Illumina	https://www.illumina.com/systems/sequencing-platforms/hiseq-x.html
MACSQuant 10 (ver 2.13.2)	Miltenyi Biotec	https://www.miltenyibiotec.com/US-en/products/macsquant-analyzer-10.html
AXR confocal microscopy	Nikon	http://www.microscope.healthcare.nikon.com
Jeol 1400 plus TEM	Jeol USA	https://www.jeol.com/product/scientific/tem
Illumina Novaseq™ 6000 platform	Illumina	https://www.illumina.com/systems/sequencing-platforms/
GraphPad Prism9 software	GraphPad Software	https://www.graphpad.com/
CytoFLEX S (Ver. 2.10)	Beckman Coulter	https://www.beckman.com/flow-cytometry/research-flow-cytometers/cytoflex
Xenogen IVIS-Lumina	Caliper life science Inc.	http://www.caliperls.com/assets/014/7156.pdf
Flowjo software (ver 10.7.1)	Flowjo, LLC	https://www.flowjo.com/
Cytoscape	NIGMS	https://cytoscape.org/
Image J	NIH	https://imagej.nih.gov/ij/download.html
OMERO	OME	https://www.openmicroscopy.org/omero/
FCS express	De Novo	https://denovosoftware.com/
R statistical language (ver. 3.3.3)	R foundation	https://www.r-project.org/
